# Automated whole genome sequencing platform for bacterial strain typing in clinical microbiology laboratories

**DOI:** 10.1128/jcm.00178-25

**Published:** 2025-04-22

**Authors:** Rachel L. Siddall, Jordan C. Starkey, Robin Patel

**Affiliations:** 1 Division of Clinical Microbiology, Department of Laboratory Medicine and Pathology, Mayo Clinic 315212, Rochester, Minnesota, USA; 2 Department of Medicine, Division of Public Health, Infectious Diseases, and Occupational Medicine, Mayo Clinic 6915, Rochester, Minnesota, USA; Maine Medical Center Department of Medicine, Portland, Maine, USA

**Keywords:** whole genome sequencing, laboratory automation, core genome multilocus sequence typing

## Abstract

Nosocomial outbreaks impact patient safety and place an economic burden on healthcare facilities. Laboratory testing plays a crucial role in outbreak investigation and guides containment efforts. In recent years, whole genome sequencing (WGS) methods have replaced traditional typing methods due to higher resolution and simplified workflows. Though an improvement from previous methods, bacterial WGS is time-consuming with manual DNA extraction and library preparation and long sequencing times for paired-end sequence data. Here, a fully automated library preparation and sequencing solution, the Clear Dx^TM^ WGS platform (Clear Labs, San Carlos, CA), was compared to a more manual library preparation and sequencing approach using 226 isolates representing 18 bacterial species. Sequence data were analyzed using SeqSphere+ (Ridom, Münster, Germany), and the results of the two methods were compared. Of the 224 isolate sequences analyzed, 222 (99%) showed concordant isolate groupings, and, overall, the results of the two approaches were statistically similar by comparison of distance matrices. The automated workflow reduced turnaround time by 16–19 h and eliminated 3 h of manual labor while decreasing costs by an estimated 34%–57% depending on the number of isolates run. This study demonstrates the advantages of integrating automation into bacterial WGS workflows.

**IMPORTANCE:**

An automated platform for bacterial nucleic acid extraction and whole genome sequencing was compared to a manual method for bacterial strain typing. The two approaches yielded nearly equivalent results, with the automated approach providing improvement in turnaround time and cost, with less manual pipetting.

## INTRODUCTION

Nosocomial transmission in healthcare settings puts vulnerable patients at risk for infectious diseases. Immunosuppressed patients, infants, the elderly, and those requiring catheters or mechanical ventilation are especially susceptible to nosocomial infection. Suspected outbreaks may require costly and time-consuming investigations while negatively impacting patient outcomes (1). The goals of a suspected outbreak investigation are twofold: eliminate the disease outbreak and understand outbreak origination and transmission to prevent further occurrences (2). It must be determined if the situation is truly an outbreak (i.e., involving a single bacterial strain) or a cluster of infections caused by multiple unrelated strains. Single-strain outbreaks may prompt increased screening and decolonization, isolation of carriers to prevent continued transmission, and/or enhanced sterilization and decontamination efforts (3).

Recently, whole genome sequencing (WGS) has become a widely used tool for bacterial strain typing (4), replacing serotyping, pulsed-field gel electrophoresis (PFGE), restriction fragment length polymorphism (RFLP), and multi-locus sequence typing (MLST) methods (5, 6). The older methods varied in cost, manual labor requirements, performance time, discriminatory power, and reproducibility of results (5). WGS methods provide high-resolution results, offering idealized differentiation of closely related isolates, and, with suitable sequencing approaches and bioinformatics tools, can provide rapid results (4). Genomic analysis of the derived sequences offers the added potential advantage of predicting antimicrobial resistance, defining underlying resistance mechanisms, and identifying virulence factors (7).

With the increasing use of WGS in clinical and public health laboratories, a need for process improvements has emerged. The Clear Dx^TM^ instrument (Clear Labs, Inc., San Carlos, CA) is a fully automated platform, comprised of a modified Microlab STAR liquid handler (Hamilton Company, Reno, NV), thermocyclers, and onboard sequencing capabilities with either Illumina iSeq100 (Illumina, Inc., San Diego, CA) or MinION sequencers (Oxford Nanopore Technologies, UK). During the SARS-CoV-2 pandemic, the City of Milwaukee Health Department Laboratory implemented the Clear Dx ^TM^ SARS-CoV-2 WGS assay (Clear Labs, Inc.) for genomic surveillance and variant tracking and demonstrated a reduction in turnaround time from two weeks with manual preparation to five days (8). The SARS-CoV-2 WGS assay was also employed to sequence the virus from wastewater for epidemiological investigation of disease prevalence and variant detection (9, 10). The platform’s assay for WGS of *Mycobacterium tuberculosis* and non-tuberculous mycobacterial isolates provides early identification and antimicrobial resistance prediction (11).

Since 2017, Mayo Clinic has clinically employed a WGS method with core genome multilocus sequence typing (cgMLST) analysis to determine the relatedness of bacterial isolates of *Staphylococcus aureus, Acinetobacter baumannii*, *Klebsiella pneumoniae*, *Legionella pneumophila, Clostridioides difficile*, *Escherichia coli*, *Enterobacter cloacae* complex, *Campylobacter jejuni/coli*, *Enterococcus faecium*, *Enterococcus faecalis*, *Streptococcus pyogenes*, *Serratia marcescens*, *Pseudomonas aeruginosa*, *Streptococcus agalactiae*, *Staphylococcus lugdunensis*, *Staphylococcus epidermidis*, and *Cutibacterium acnes*. The method has been used to investigate the epidemiology of methicillin-resistant *S. aureus* (MRSA) and methicillin-susceptible *S. aureus* (MSSA) in the neonatal intensive care unit (NICU), assess for potential nosocomial transmission of *S. aureus* bacteremia isolates, and track *C. difficile* (12–17). *K. pneumoniae*, multidrug-resistant *P. aeruginosa*, *A. baumannii*, and *E. coli* isolates have also undergone typing with cgMLST analysis with results described in the literature (18–21).

Despite the advantages of WGS, it requires several hours of hands-on technologist time, highly trained staff, extensive manual pipetting, and long sequencing times, all of which contribute to lengthy turnaround times and negatively impact cost-effectiveness. Integrating automation into the workflow provides opportunities for standardization, quality improvement, ergonomic benefits, increased efficiency, and decreased costs. To address the limitations of a manual WGS workflow, the Clear Dx^TM^ instrument using the Clear Dx^TM^ Microbial Surveillance WGS v2.0 protocol was compared to a manual WGS process for bacterial strain typing to evaluate the alignment of results, turnaround time, cost, and workflow simplification.

## MATERIALS AND METHODS

### Bacterial isolates

Two hundred twenty-six bacterial isolates were studied, including 72 *S*. *aureus,* 8 *A. baumannii*, 8 *K. pneumoniae*, 8 *L. pneumophila,* 8 *C. difficile*, 8 *E. coli*, 4 *E. cloacae* complex, 16 *Campylobacter* species (14 *C*. *jejuni* and 2 *C. coli*), 12 *E. faecium*, 7 *E. faecalis*, 12 *S*. *pyogenes*, 8 *S*. *marcescens*, 10 *P. aeruginosa*, 9 *S*. *agalactiae*, 12 *S*. *lugdunensis*, 12 *S*. *epidermidis*, and 12 *C*. *acnes*. Isolates were sourced from the Minnesota Department of Health (MDH), the Antibacterial Resistance Leadership Group (ARLG), Mayo Clinic clinical and research laboratories, or a French hospital microbiology laboratory (Table S1).

### Culture methods

Isolates were subcultured from freezer storage at −80°C. *Campylobacter* isolates were cultured on chocolate agar (Remel, Lenexa, KS) and incubated for 18–24 h at 42°C in a microaerophilic atmosphere. *L. pneumophila* isolates were cultured on buffered charcoal yeast extract agar (Remel, Lenexa, KS) and incubated for 48 h at 35–37°C in an ambient atmosphere. *C. difficile* and *C. acnes* isolates were cultured on CDC anaerobic 5% sheep blood agar (Becton, Dickinson and Company, Franklin Lakes, NJ) and incubated for 48 h at 35–37°C in an anaerobic atmosphere. All other isolates were cultured on tryptic soy agar with 5% sheep blood (Remel, Lenexa, KS) and incubated for 18–24 h at 35–37°C in 5% CO_2_.

### Manual WGS method

Bacterial DNA was extracted with the *Quick*-DNA^TM^ Fungal/Bacterial MiniPrep Kit (Zymo Research, Irvine, CA). DNA was quantified with a Quantus^TM^ Fluorometer using the QuantiFluor dsDNA System dye (Promega, Madison, WI) and diluted to 0.2 ng/µL. Isolates underwent library preparation with the Nextera XT DNA Library Preparation Kit (Illumina, Inc., San Diego, CA) and were sequenced on a MiSeq instrument (Illumina, Inc.) with a 2 × 250 bps cycle kit. Target coverage for all species was 100× apart from *C. difficile,* which had a target coverage of 200×. This will be hereafter referred to as the manual WGS method.

### Automated WGS method

One 1 µL loopful of Gram-negative bacterial isolates or four 1 µL loopfuls of Gram-positive bacterial isolates were resuspended in 400 µL of resuspension buffer (Clear Labs, Inc.) in a 2 mL tube containing extraction beads, vortexed for 30 s, and 100 µL of supernatant transferred to a 96-well plate. Isolates were sequenced using the “Isolate” sample input type of the Clear Dx^TM^ Microbial Surveillance WGS v2.0 protocol on the Clear Dx^TM^ platform. Samples, reagents, and consumables were loaded on the instrument per the manufacturer’s instructions. Because it was hypothesized that coverage could be reduced from that of the manual WGS method, the target coverage was reduced to 30–80× depending on the genome size of the organism and the capacity of the iSeq100 sequencers. The actual depth of coverage achieved ranged from 48× to 171× with an average of 88×.

Following run initiation, the fully automated platform performed cell lysis, nucleic acid extraction, and library preparation and loaded the sequencing cartridges onto the two Illumina iSeq100 sequencers (Illumina, Inc.) with 2 × 150 bp cycle kits. Following run completion, the web application provided species-level identification, average Q score, mean coverage, and a downloadable FASTQ file for each sample. The species-level identification provided by the Clear Labs software matched matrix-assisted laser desorption ionization time-of-flight (MALDI-TOF) mass spectrometry identification of all isolates. The run time for the 23 runs performed with the fully automated workflow ranged from 26 to 32 h and averaged 28 h. This will be hereafter referred to as the automated WGS method. This automated WGS platform is for research use only and has not been cleared or approved for clinical diagnostics.

### Data analysis

Sequences from the two WGS methods were analyzed using SeqSphere+ software v10.0.5 (Ridom, Münster, Germany). Briefly, paired-end .fastq.gz files were uploaded to SeqSphere+ and assembled with a SKESA v2.3.0 *de novo* assembler. Assembled sequences were processed with species-specific cgMLST pipelines within SeqSphere+ and allelic differences between isolates were used to construct minimum spanning trees (MSTs) from the cgMLST typing data table for the visualization of isolate relationships.

Previously validated species-specific cgMLST allelic difference cutoffs used in the Mayo Clinic clinical practice were applied to classify isolates as related, possibly related, or unrelated (Table 1). To set allelic difference relatedness thresholds, isolates from outbreaks and contemporaneous isolates from the same location were sequenced using the manual WGS method. The cgMLST type for each isolate determined by SeqSphere+ was then compared to data from other typing methods performed on the isolates, including PFGE, MLST, *Staphylococcus* protein A (*spa*) type, ribotype, *bla*_KPC_ gene type, PCR-electrospray ionization mass spectrometry (PCR/ESI-MS), sequence type (ST)-specific PCR, serogroup, M protein gene (*emm*) type, and/or in-house analytical pipelines. Varying allelic relatedness thresholds were applied to establish cutoffs best correlating with clusters from known outbreaks and results of previous typing methods (3, 12, 16–22).

**TABLE 1:** Thresholds for allelic differences used to classify isolates as “related,” “possibly related,” or “unrelated”^*a*^

	Number of allelic differences		
Organism	Related	Possibly related	Unrelated
*Staphylococcus aureus* (3, 12, 16)	≤8	9–29	≥30
*Acinetobacter baumannii* (20)	≤9	10–200	≥201
*Klebsiella pneumoniae* (18)	≤15	16–50	≥51
*Legionella pneumophila*	≤4	5–30	≥31
*Clostridioides difficile* (17)	≤6	7–50	≥51
*Escherichia coli* (21)	≤10	11–30	≥31
*Enterobacter cloacae* complex	≤15	16–50	≥51
*Campylobacter jejuni/coli*	≤10	11–60	≥61
*Enterococcus faecium*	≤7	8–30	≥31
*Enterococcus faecalis*	≤7	8–30	≥31
*Streptococcus pyogenes*	≤20	21–100	≥101
*Serratia marcescens* (22)	≤12	13–100	≥101
*Pseudomonas aeruginosa* (19)	≤6	7–100	≥101
*Streptococcus agalactiae*	≤20	21–100	≥101
*Staphylococcus lugdunensis*	≤8	9–29	≥30
*Staphylococcus epidermidis*	≤8	9–29	≥30
*Cutibacterium acnes*	≤5	6–50	≥51

^*a*^ These thresholds were developed as described in the text by sequencing bacterial isolates from outbreaks and non-outbreak isolates from the same time and location, with references shown, where data have been published. Sequences were assembled in SeqSphere+ and analyzed with core genome multilocus sequence typing (cgMLST). cgMLST analysis was compared to the results of PFGE and/or other typing methods. Statistical analysis was performed to determine cutoffs for grouping isolates as related, possibly related, or unrelated.

Sequences generated from each WGS method were analyzed in two ways. First, MSTs were generated for the manual and automated WGS methods using the validated allelic difference cutoffs. MSTs for each organism were compared to determine if isolates were grouped the same way by the two methods (i.e., related, possibly related, and unrelated). Next, distance matrices showing allelic differences between isolates were created for each organism and each sequencing method in SeqSphere+. Two *S*. *aureus* isolates were excluded from the final analysis (BTP 246 and USA200). One was contaminated with another organism on the agar plate following subculture, while the other generated no sequence data due to a Clear Dx^TM^ instrument pipetting error.

To compare turnaround time and manual labor between processes, both overall and hands-on time required were calculated for each step of the manual and automated processes (Table 2). The processes were broken down into sample preparation, instrument setup (automated process only), library preparation, sequencing, and analysis, with the sum of all steps resulting in the total time.

**TABLE 2:** Average time per step for manual and automated whole genome sequencing (WGS) methods

Step	Manual WGS method		Automated WGS method	
	Hands-on time	Additional automated analytical time	Hands-on time	Additional automated analytical time
Sample preparation	30 min	Not applicable	30 min	Not applicable
Instrument setup	Not applicable	Not applicable	30 min	Not applicable
Library preparation	3 hours	Not applicable	Not applicable	7 h
Sequencing	30 min	39–42 h	Not applicable	19 h
Bioinformatics analysis	1 h	Not applicable	1 h	Not applicable
Total	5 h	39–42 h	2 h	26 h
Total time	44–47 h		28 h	

Cost differences were compared by calculating labor, reagents, consumables, and standard overhead for each method (Table 3). Costs per test for runs of 1–12 isolates were calculated to determine cost differences between manual and automated WGS processes. The cost of new equipment, equipment depreciation, and service contracts were excluded from the analysis.

**TABLE 3:** Cost per test by number of isolates per run for manual and automated whole genome sequencing (WGS) methods

Batch size (number of isolates)		Cost of components per sample based on batch size											
		1	2	3	4	5	6	7	8	9	10	11	12
Manual WGS method	Technologist labor	$550	$275	$183	$138	$110	$92	$79	$69	$61	$55	$50	$46
	Materials	$2,555	$1,360	$962	$762	$643	$563	$506	$463	$430	$404	$382	$364
	Laboratory director review	$11	$11	$11	$11	$11	$11	$11	$11	$11	$11	$11	$11
	Overhead	$35	$35	$35	$35	$35	$35	$35	$35	$35	$35	$35	$35
	Total cost per test	$3,151	$1,681	$1,191	$946	$799	$701	$631	$578	$537	$505	$478	$456
Automated WGS method	Technologist labor	$41	$21	$14	$10	$8	$7	$6	$5	$5	$4	$4	$3
	Materials	$2,000	$1,000	$667	$500	$400	$333	$286	$250	$222	$200	$182	$167
	Laboratory director review	$11	$11	$11	$11	$11	$11	$11	$11	$11	$11	$11	$11
	Overhead	$35	$35	$35	$35	$35	$35	$35	$35	$35	$35	$35	$35
	Total cost per test	$2,087	$1,067	$727	$556	$454	$386	$338	$301	$273	$250	$232	$216
Difference between manual and automated WGS methods		$1064	$614	$464	$390	$345	$315	$293	$277	$264	$255	$246	$240
Percent cost reduction		34%	37%	39%	41%	43%	45%	46%	48%	49%	50%	51%	53%

## RESULTS

Overall, species-level identities of isolates generated using the automated WGS method were in full concordance (100%) with identities reported by MALDI-TOF mass spectrometry. The results of cgMLST analysis from the two methods were also similar. Of the 224 isolates analyzed, 222 (99%) showed concordant results between the two methods when comparing relatedness groupings (i.e., related, possibly related, and unrelated), with two *S*. *epidermidis* isolates (MBRL2787 and MBRL2788) differing by a single allelic difference between the two methods, moving them from “possibly related” with the manual WGS method to “related” with the automated WGS method. Detailed MSTs are provided in Fig. S1–S34 in the supplementary material.

**Fig 1:**
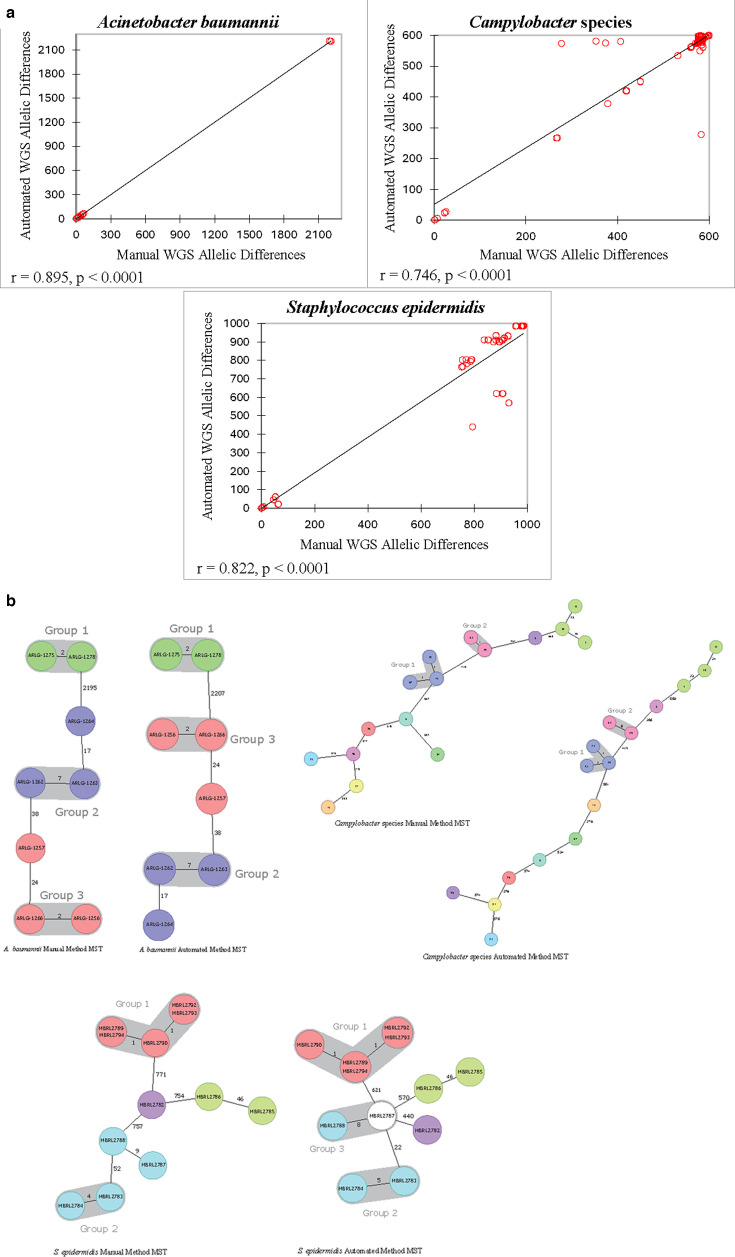
(a) Mantel correlations between manual and automated whole genome sequencing (WGS) methods. Scatter plots illustrating the allelic differences between isolates using the manual and automated WGS methods with correlating Mantel statistics for bacterial species with r values less than 0.9. (b) Minimum spanning trees (MSTs) for *Acinetobacter baumannii*, *Campylobacter* species, and *Staphylococcus epidermidis*. Although *A. baumannii*, *Campylobacter* species, and *S. epidermidis* demonstrated correlation coefficients of *r* = 0.895, 0.746, and 0.822, respectively, upon Mantel Spearman analysis, the variation in allelic differences between methods minimally affected final results. The two methods show concordant relatedness groupings for *A. baumannii* and *Campylobacter* species. Two *S. epidermidis* isolates (MBRL2787 and MBRL2788) differed by a single allelic difference between the two methods, moving them from “possibly related” with the manual method to “related” with the automated method.

The Mantel Spearman correlation was calculated between the paired distance matrices (see Tables S2–S18 for distance matrices) using a 95% CI. *S. aureus*, *K. pneumoniae*, *L. pneumophila*, *C. difficile*, *E. coli*, *E. faecium, E. faecalis*, *S. pyogenes*, *S. marcescens*, *P. aeruginosa*, *S. agalactiae*, *S. lugdunensis*, and *C. acnes* demonstrated correlation coefficients above 0.900 with *P*-values of <0.0001, demonstrating a high degree of similarity between manual and automated WGS methods. *A. baumannii*, *Campylobacter* species, and *S. epidermidis* showed lower correlation coefficients (*r* = 0.895, 0.746, and 0.822, respectively) indicating more variation between manual and automated WGS methods (Fig. 1). The small sample size (*n* = 4) for *E. cloacae* complex resulted in an insignificant *P*-value (*P* = 0.082), but this grouping had the highest correlation between methods (*r* = 1).

The use of the Clear Dx^TM^ platform improved turnaround time from 44–47 h with the manual process to 26–32 h (average of 28 h) (Table 2). The biggest improvement stemmed from only 19 h of sequencing on the iSeq sequencers on the Clear Labs instrument compared to 39–42 h on the MiSeq sequencer used with the manual approach. Because of the smaller sequencing capacity of iSeq sequencing flow cells, runs can be performed more frequently instead of batching isolates to accommodate the larger capacity of the MiSeq sequencing flow cells. Additionally, automation of the manual library preparation procedure eliminated 3 h of manual work and reduced the number of manual pipetting steps.

A cost analysis accounting for labor, reagents, consumables, and standard overhead costs revealed predicted cost savings with the usage of the Clear Dx^TM^ instrument (Table 3). Automation of manual labor reduced predicted labor costs by 93% regardless of the number of isolates on a single run. For both methods, the largest cost stemmed from reagents and consumables, with library preparation kits being the most costly item. Consolidation of reagent and consumable purchases from several vendors to the Clear Labs bacterial WGS kits reduced predicted costs by 22%–54% depending on the number of isolates per run. Overall, the instrument resulted in projected cost savings of 34%–53% for run sizes of 1–12 isolates, respectively, compared to the manual process.

A final metric evaluated was the simplification of the manual WGS workflow. In the current Mayo Clinic workflow, with the manual method, isolates are extracted in one location and sent to another for library preparation and sequencing, with sequence analysis and reporting occurring in the first location. The Clear Dx^TM^ instrument allows the entire workflow to occur in one location in an enclosed environment. Additionally, automation of library preparation eliminates the need to train laboratory staff on the lengthy manual procedure while reducing the chance of pipetting errors and ergonomic strain.

## DISCUSSION

Although WGS methods for bacterial strain typing are a significant improvement from historical methods, there are opportunities for workflow optimization. Manual library preparation requiring extensive manual protocols and highly skilled staff is arguably a challenge for clinical laboratories performing WGS. Several vendors have developed platforms to automate library preparation, but these lack the ability to load the library onto a sequencer, requiring manual intervention at this step (23). Modifications to the instrument deck or software may be necessary to adapt liquid handlers to a customized library preparation workflow or the process may require validation of a manufacturer’s set workflow (23).

Incorporating automation into WGS workflows may increase the accessibility of sequencing technology to clinical laboratories by simplifying complex processes, decreasing the number of staff needed to perform testing, and improving turnaround time, providing results critical to patient care sooner. Here, it was demonstrated that an automated bacterial WGS assay can produce results that align with a more manual workflow while improving turnaround time, decreasing manual labor, providing projected cost savings, and improving overall workflow efficiency. The Clear Dx^TM^ Microbial Surveillance WGS v2.0 protocol demonstrated 99% concordance with the manual WGS method based on the grouping of isolates by relatedness. Statistical analysis of paired distance matrices demonstrated a strong positive correlation, indicating similar cgMLST results between methods. Despite r values below 0.9 calculated by Mantel Spearman correlations for the *A. baumannii*, *Campylobacter* species, and *S. epidermidis* isolates tested, the final interpretation between the two methods was minimally affected (Fig. 1b). The automated liquid handling eliminated manual library preparation and sequencer loading, minimizing human error, reducing the ergonomic strain of manual pipetting, and improving turnaround time while decreasing time spent on hands-on processes. Reduction of manual labor contributes to an overall projected decrease in cost per test, and automation provides flexibility for sequencing to occur on all laboratory shifts since the instrument functions without the need for intervention or supervision.

A limitation of this study is the small sample size. Further, isolates across all relatedness categories were not available for all species. Isolates of varied degrees of relatedness were selected, if available, and the two methods showed concordance for the isolates tested. Further testing of isolates from additional outbreaks would strengthen the evidence that the manual and automated WGS procedures produce equivalent results. Another limitation was the inability to provide a direct comparison between Illumina sequencers for the manual and automated methods; ideally, this study would have compared manual and automated sequencing processes using the same sequencers.

The development of new automated solutions for WGS will only continue to make the technology more accessible to clinical laboratories as processes become more efficient, streamlined, and affordable. With these improvements, outbreak investigations involving WGS methods will do the same, enabling rapid response and a greater understanding of pathogenicity and transmission. Finally, the automation studied herein should be applied to species identification by WGS and analysis of resistance genes and mutations from WGS data in future studies.

## Data Availability

Select isolate sequences are available in the National Center for Biotechnology Information archives under accession numbers PRJNA736958 (*E. coli*), PRJNA855929 (*P. aeruginosa*), and PRJNA809687 (*A. baumannii*). Additional sequence data, generated from isolates received from external organizations, are available from the authors upon reasonable request and with permission from those who supplied the isolates.

## SUPPLEMENTAL MATERIAL

Supplemental figures and tables jcm.00178-25-s0001.pdf Minimum spanning trees, isolate description table, and distance matrices.

ASM does not own the copyrights to Supplemental Material that may be linked to, or accessed through, an article. The authors have granted ASM a non-exclusive, world-wide license to publish the Supplemental Material files. Please contact the corresponding author directly for reuse.
