# Supplementary material for: Automated whole genome sequencing platform for bacterial strain typing in clinical microbiology laboratories
Source: J Clin Microbiol. 2025 Apr 22;63(5):e00178-25. doi: 10.1128/jcm.00178-25 (PMC12077132; doi:10.1128/jcm.00178-25)
Supplement: Supplemental figures and tables — Minimum spanning trees, isolate description table, and distance matrices. [file jcm.00178-25-s0001.pdf]

Supplemental Figure 1: *Staphylococcus aureus*
Manual Method Minimum Spanning Tree

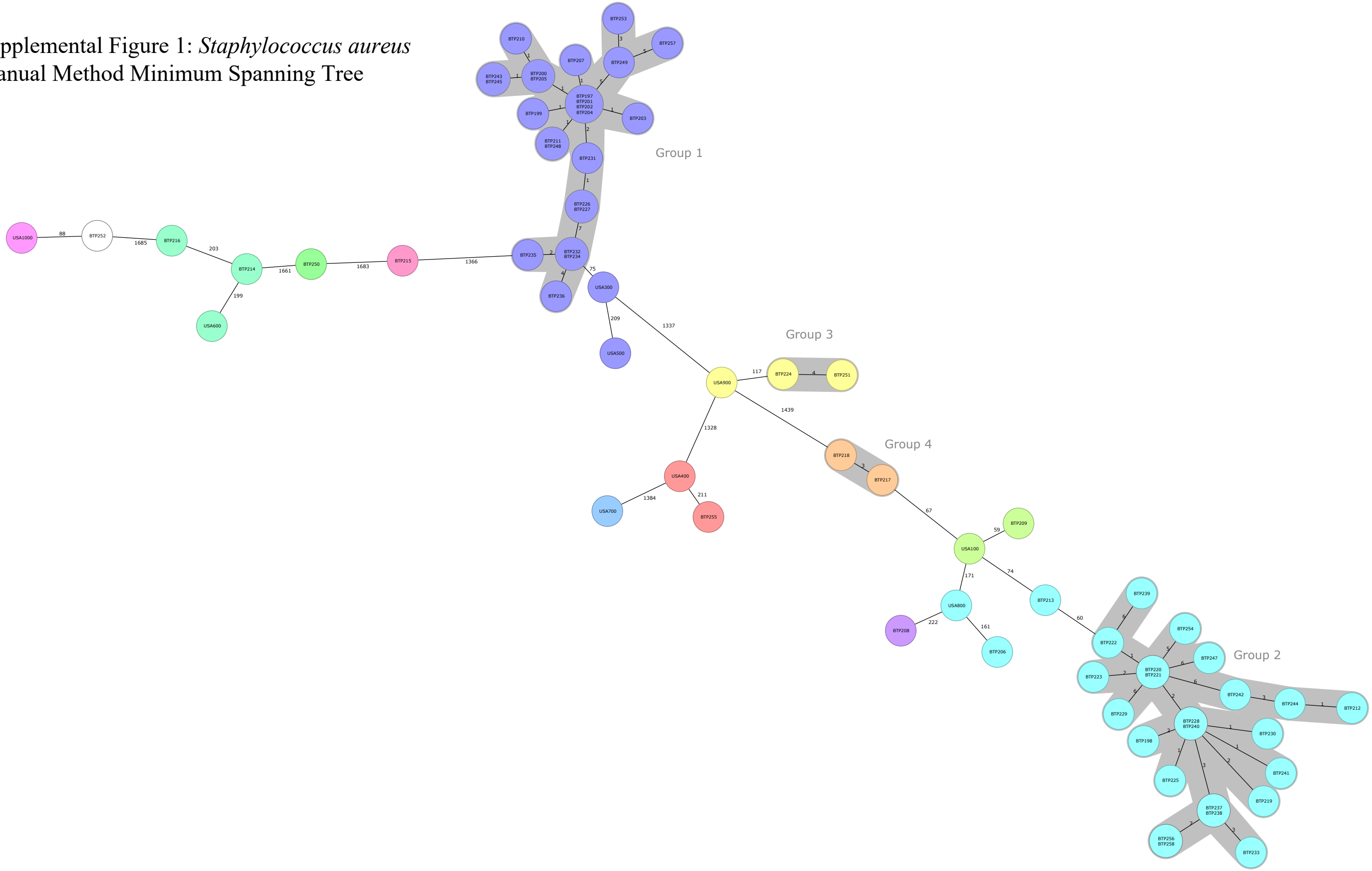

Supplemental Figure 2: *Staphylococcus aureus* Automated Method Minimum Spanning Tree

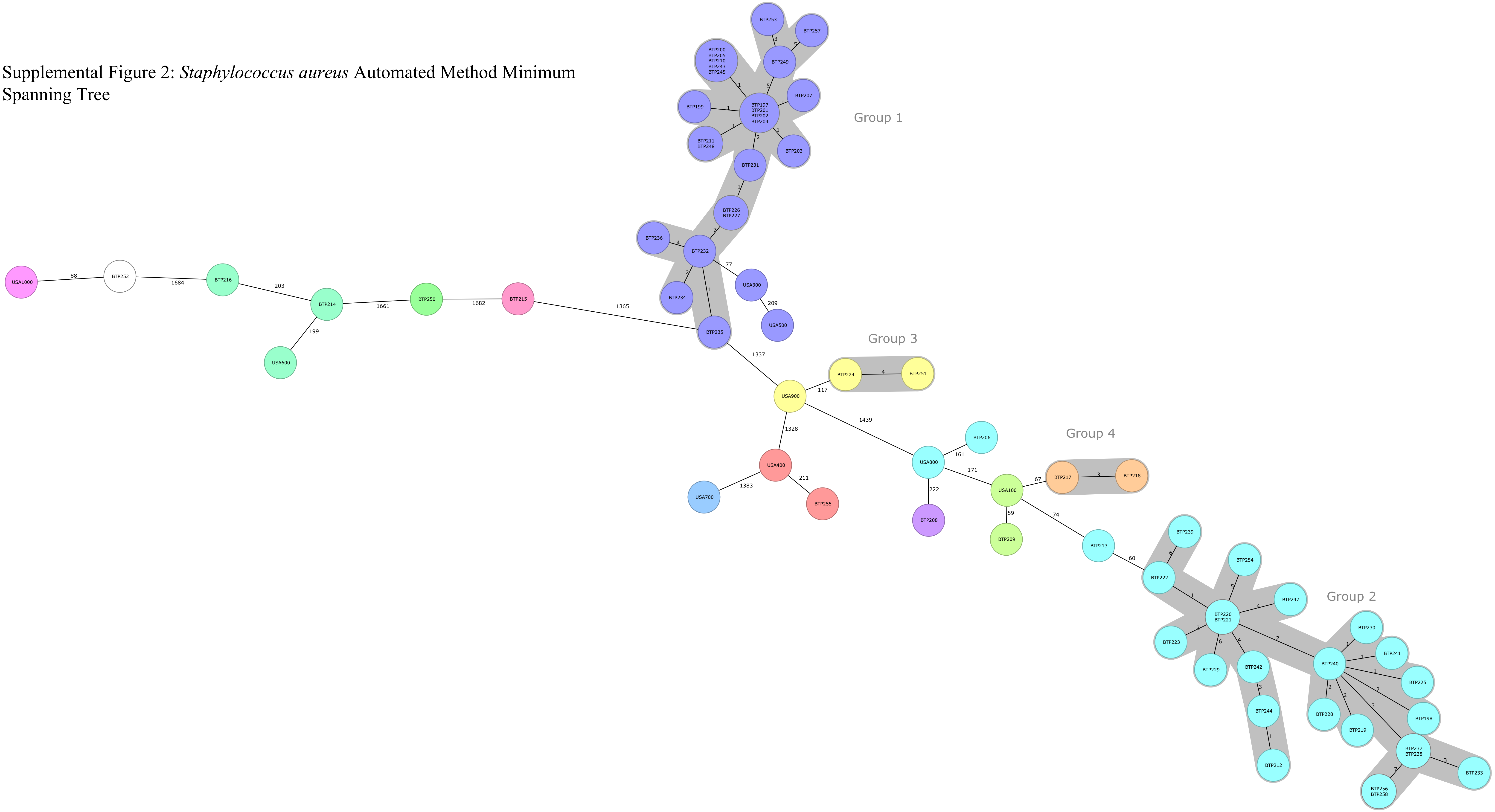

Supplemental Figure 3: *Acinetobacter baumannii* Manual Method Minimum Spanning Tree

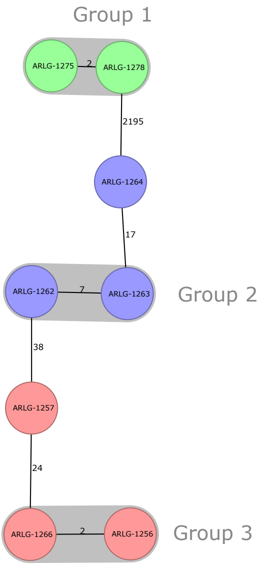

Supplemental Figure 4: *Acinetobacter baumannii* Automated Method Minimum Spanning Tree

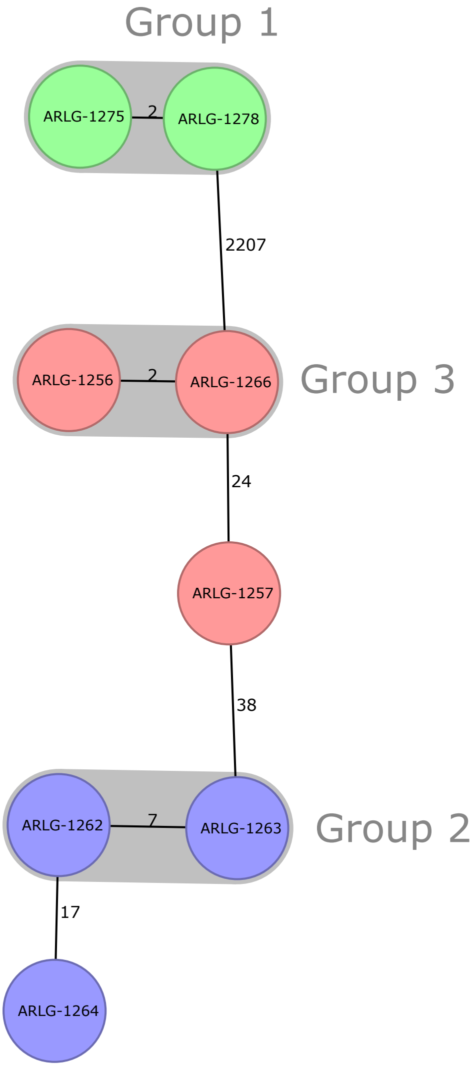

Supplemental Figure 5: *Klebsiella pneumoniae* Manual Method Minimum Spanning Tree

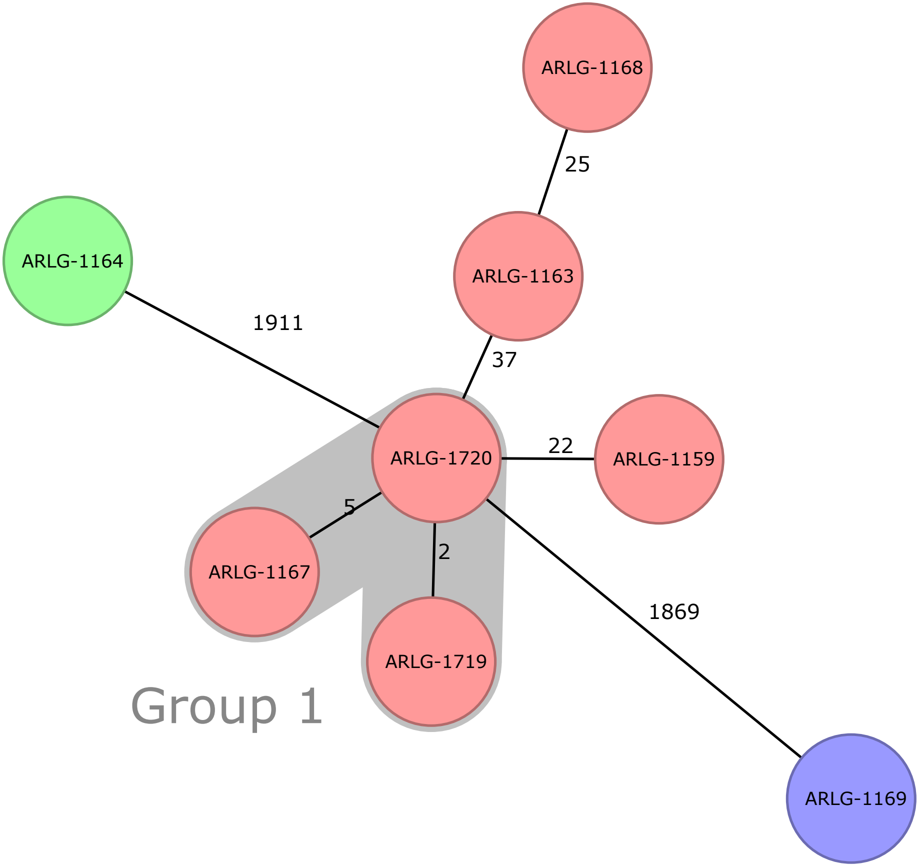

Supplemental Figure 6: *Klebsiella pneumoniae* Automated Method Minimum Spanning Tree

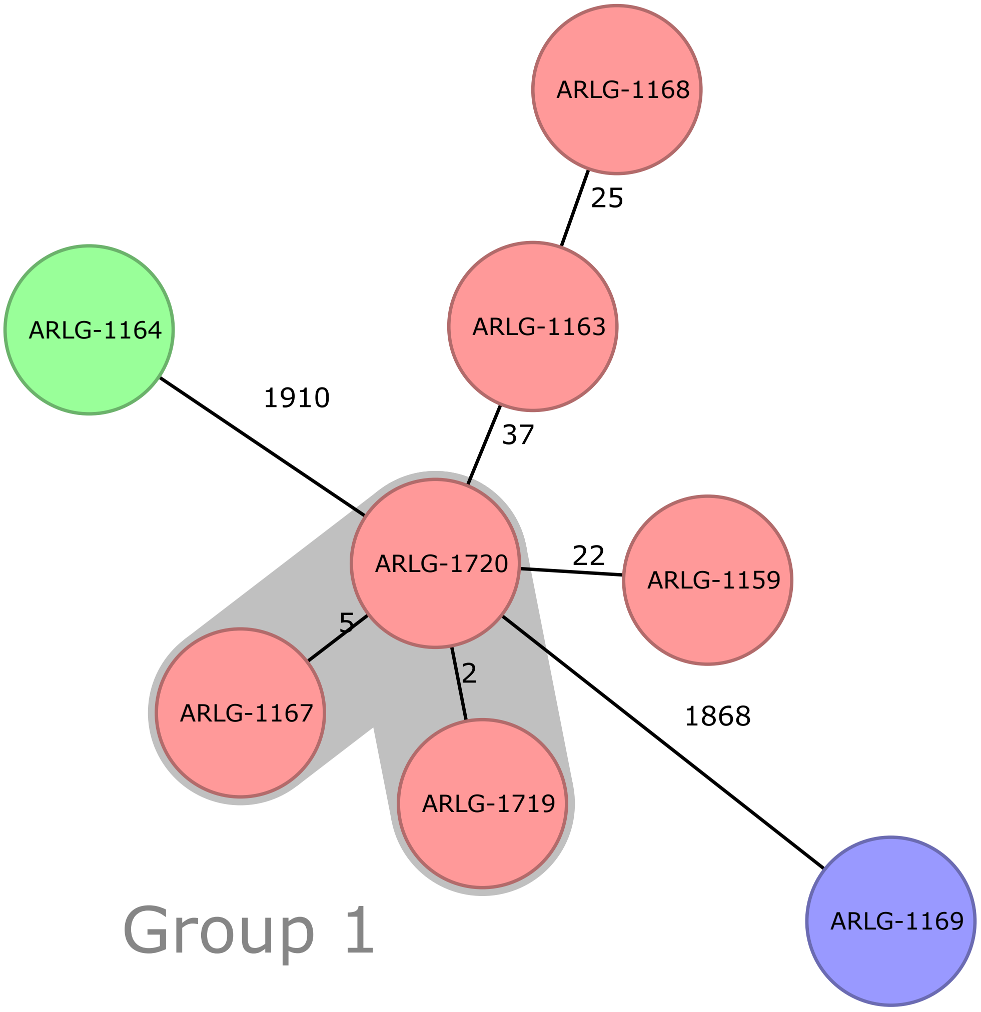

Supplemental Figure 7: *Legionella pneumophila* Manual Method Minimum Spanning Tree

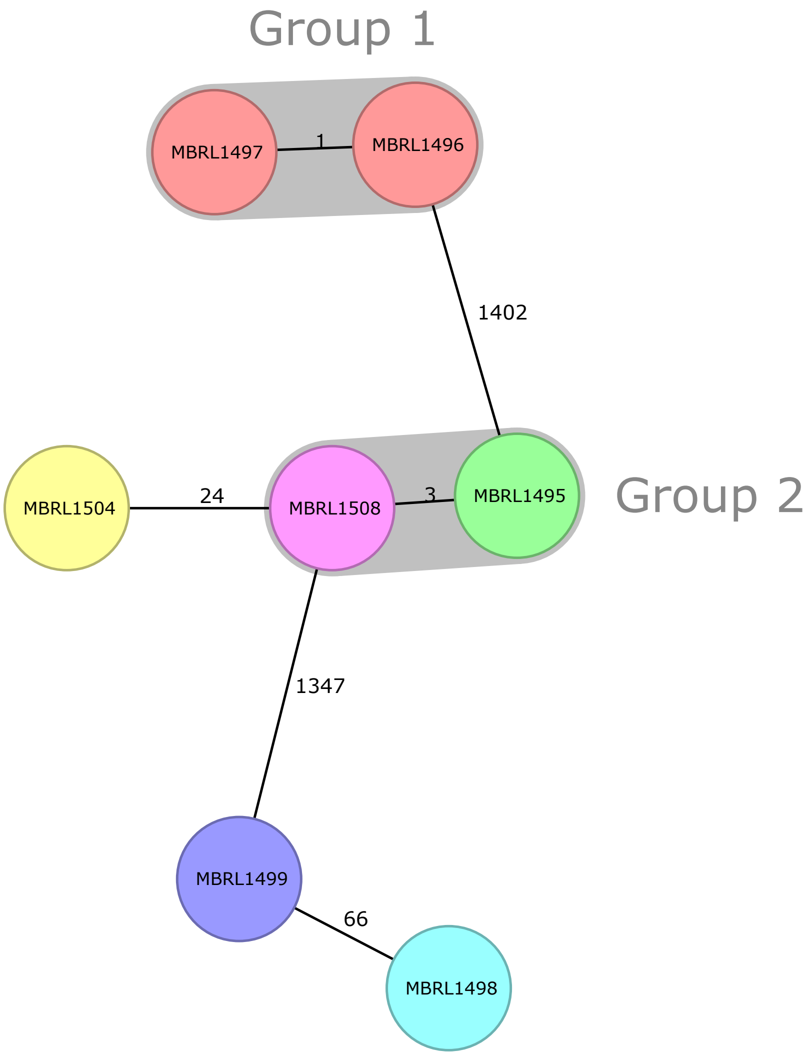

Supplemental Figure 8: *Legionella pneumophila* Automated Method Minimum Spanning Tree

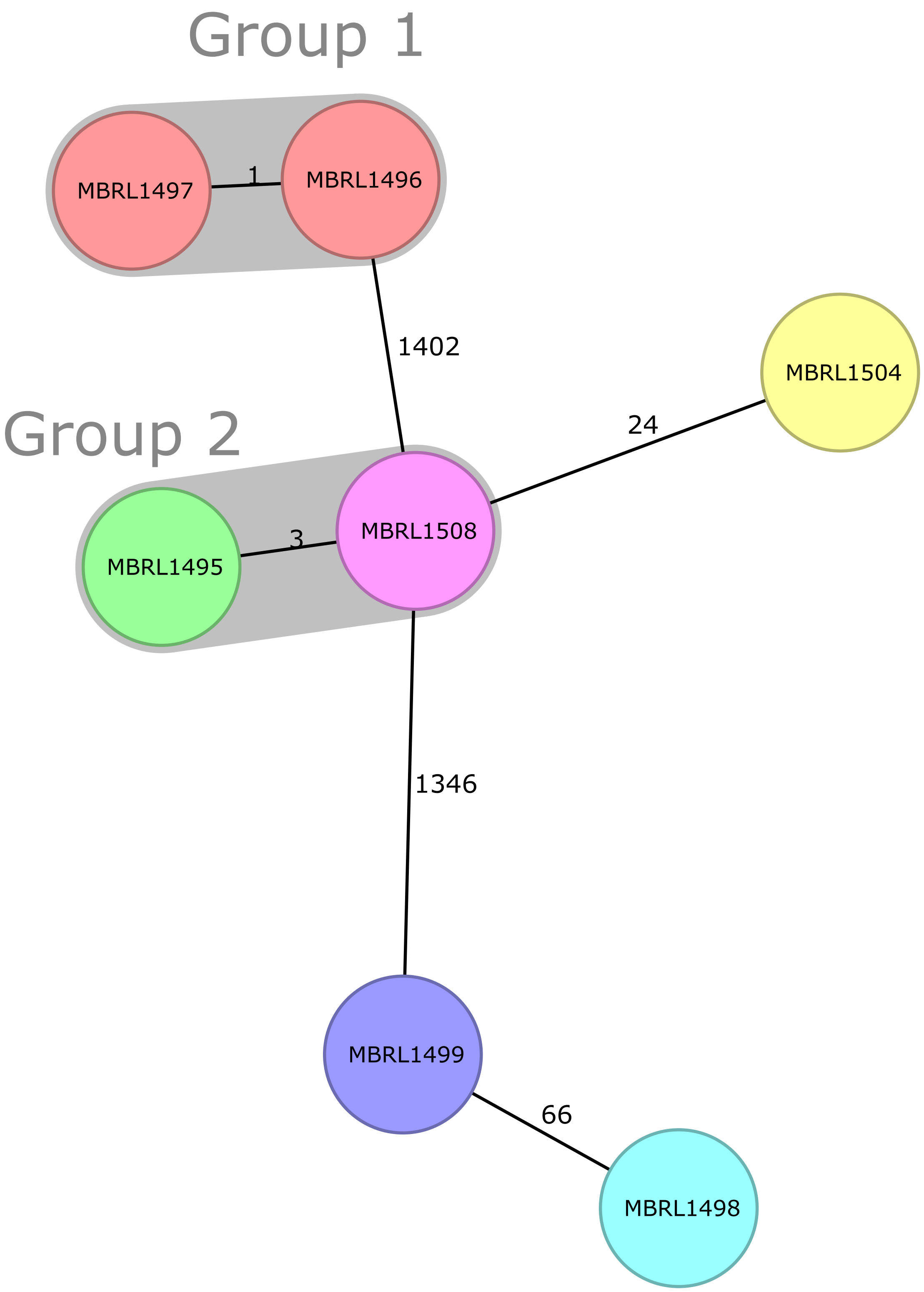

Supplemental Figure 9: *Clostridioides difficile* Manual Method Minimum Spanning Tree

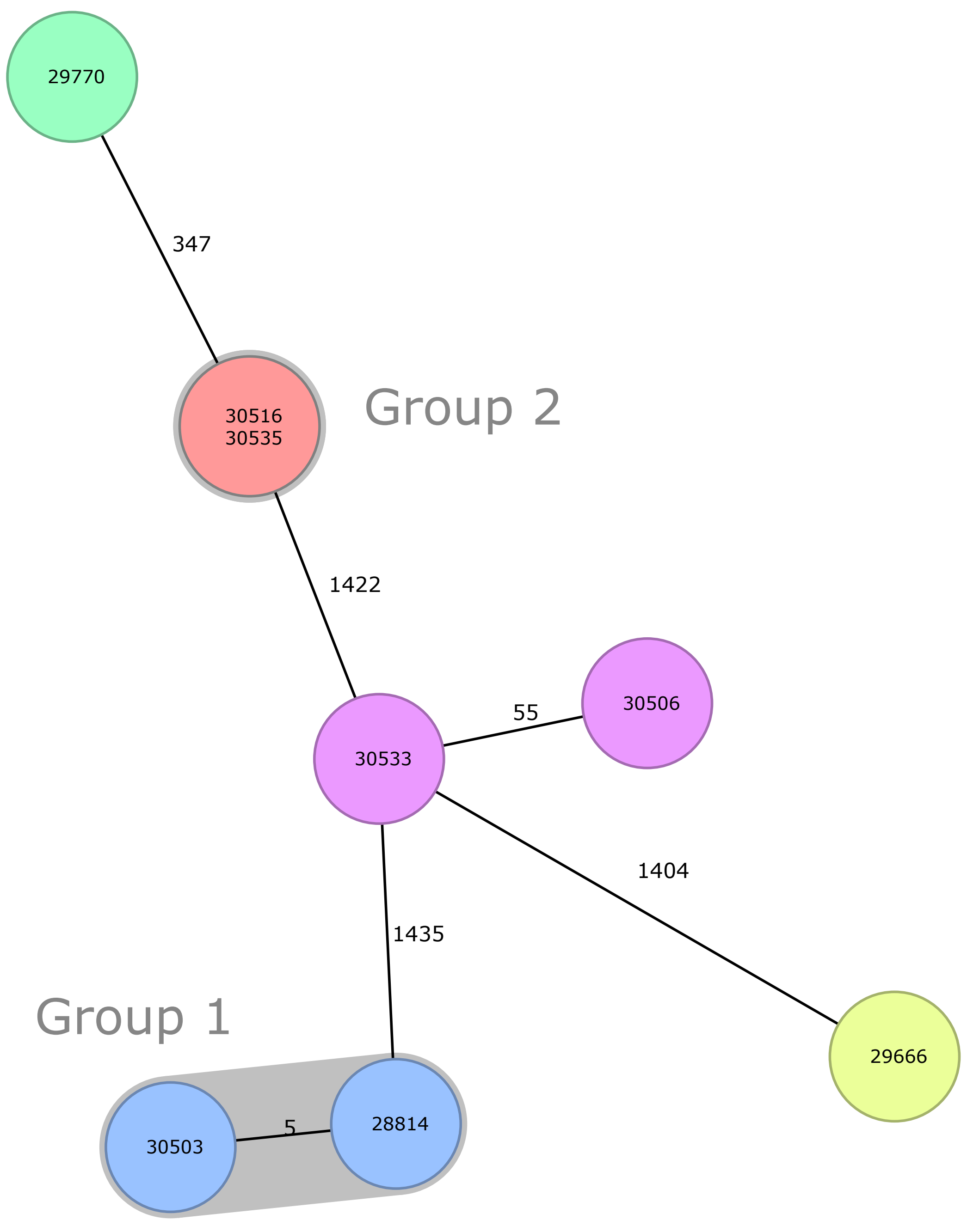

Supplemental Figure 10: *Clostridioides difficile* Automated Method Minimum Spanning Tree

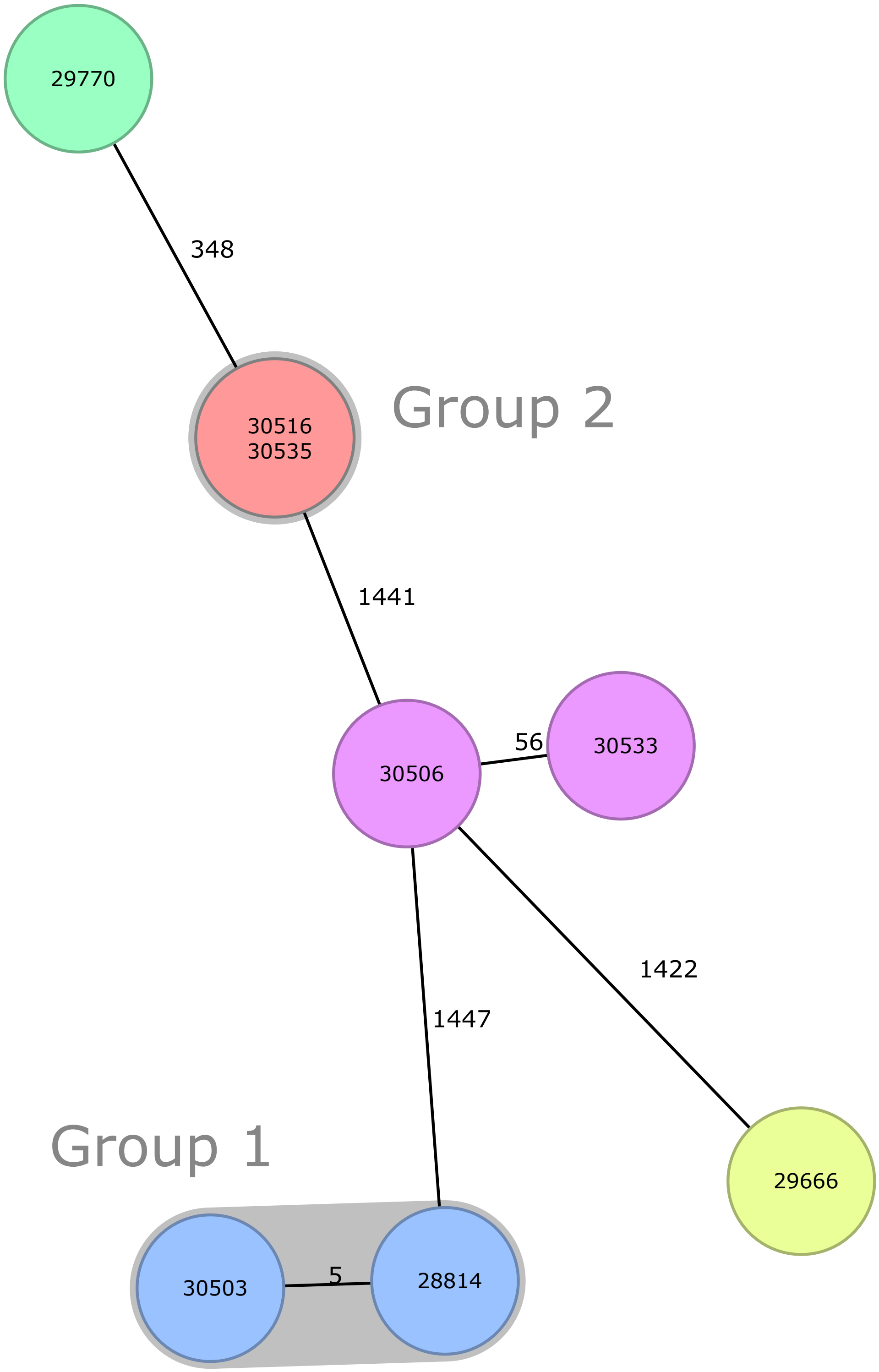

Supplemental Figure 11: *Escherichia coli* Manual Method Minimum Spanning Tree

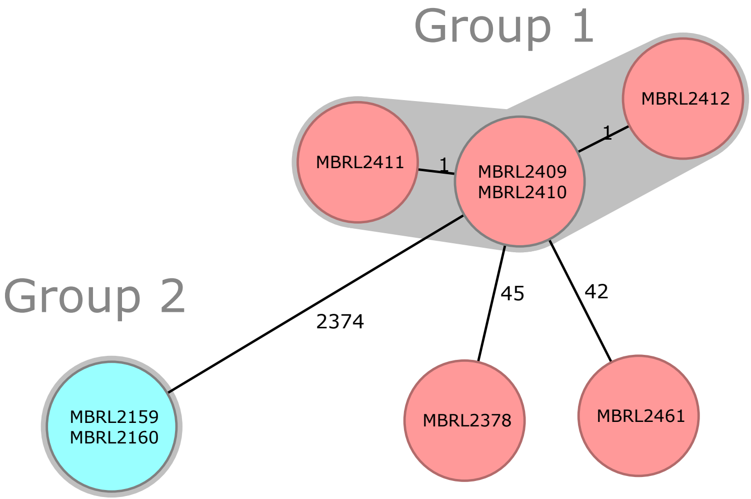

Supplemental Figure 12: *Escherichia coli* Automated Method Minimum Spanning Tree

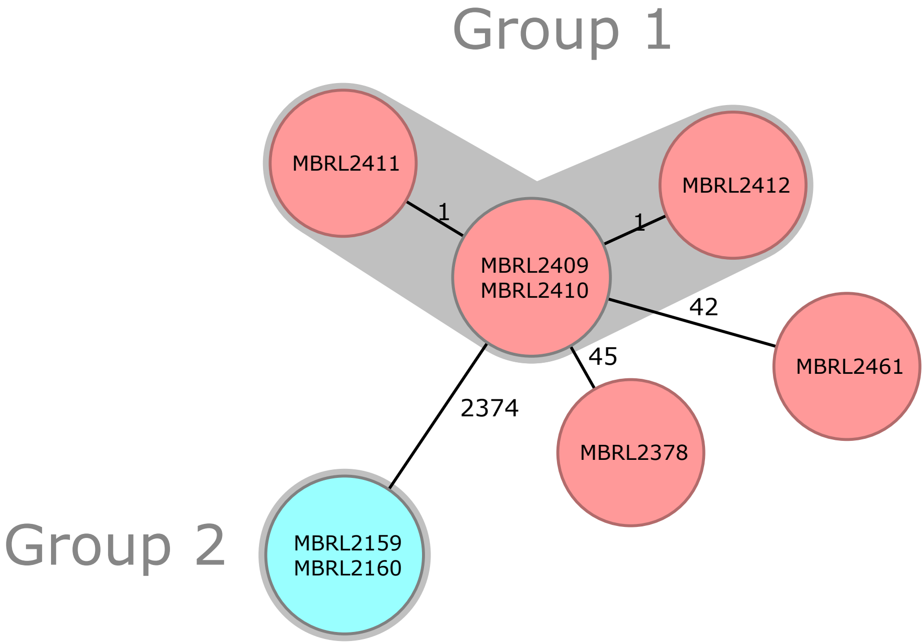

Supplemental Figure 13: *Enterobacter cloacae* complex Manual Method Minimum Spanning Tree

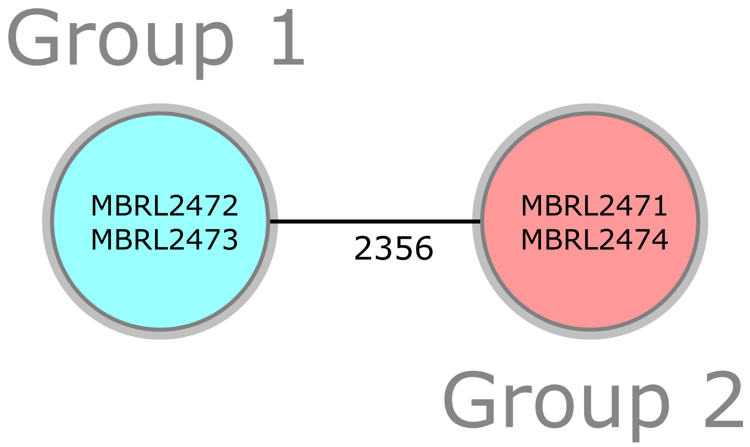

Supplemental Figure 14: *Enterobacter cloacae* complex Automated Method Minimum Spanning Tree

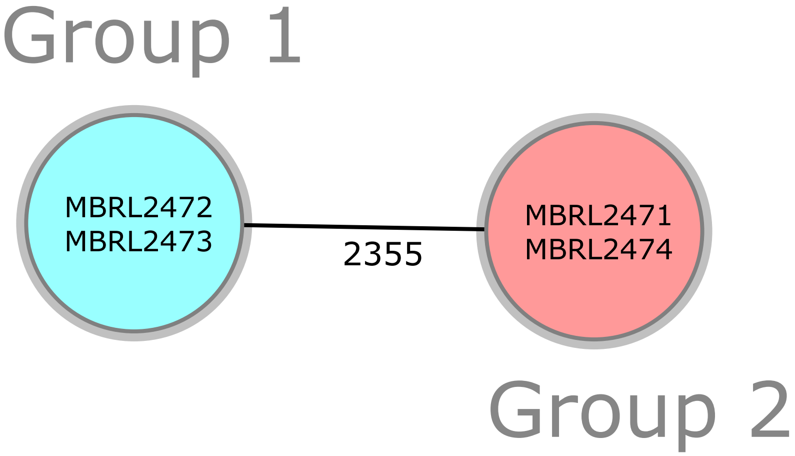

Supplemental Figure 15: *Campylobacter jejuni/coli* Manual Method Minimum Spanning Tree

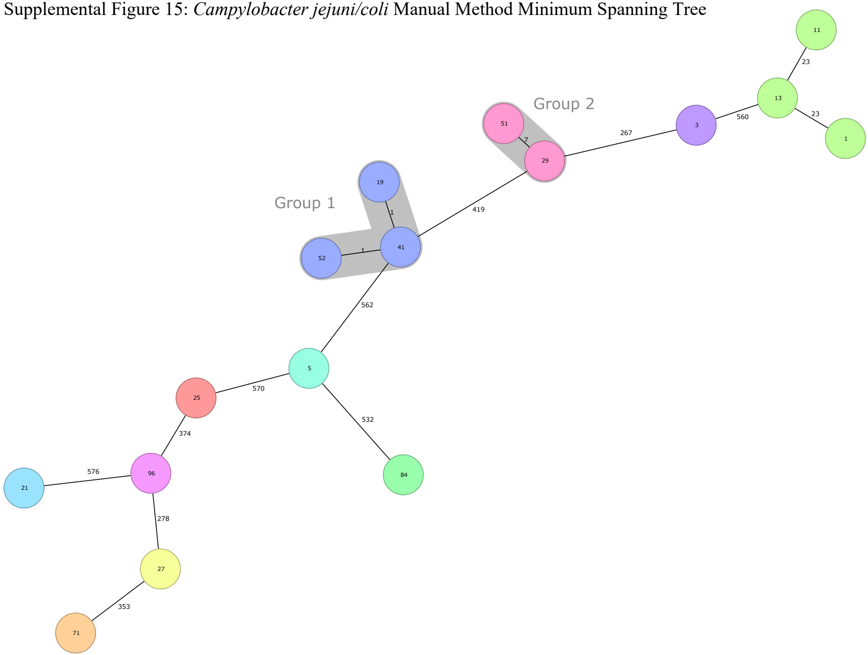

Supplemental Figure 16: *Campylobacter jejuni/coli* Automated Method Minimum Spanning Tree

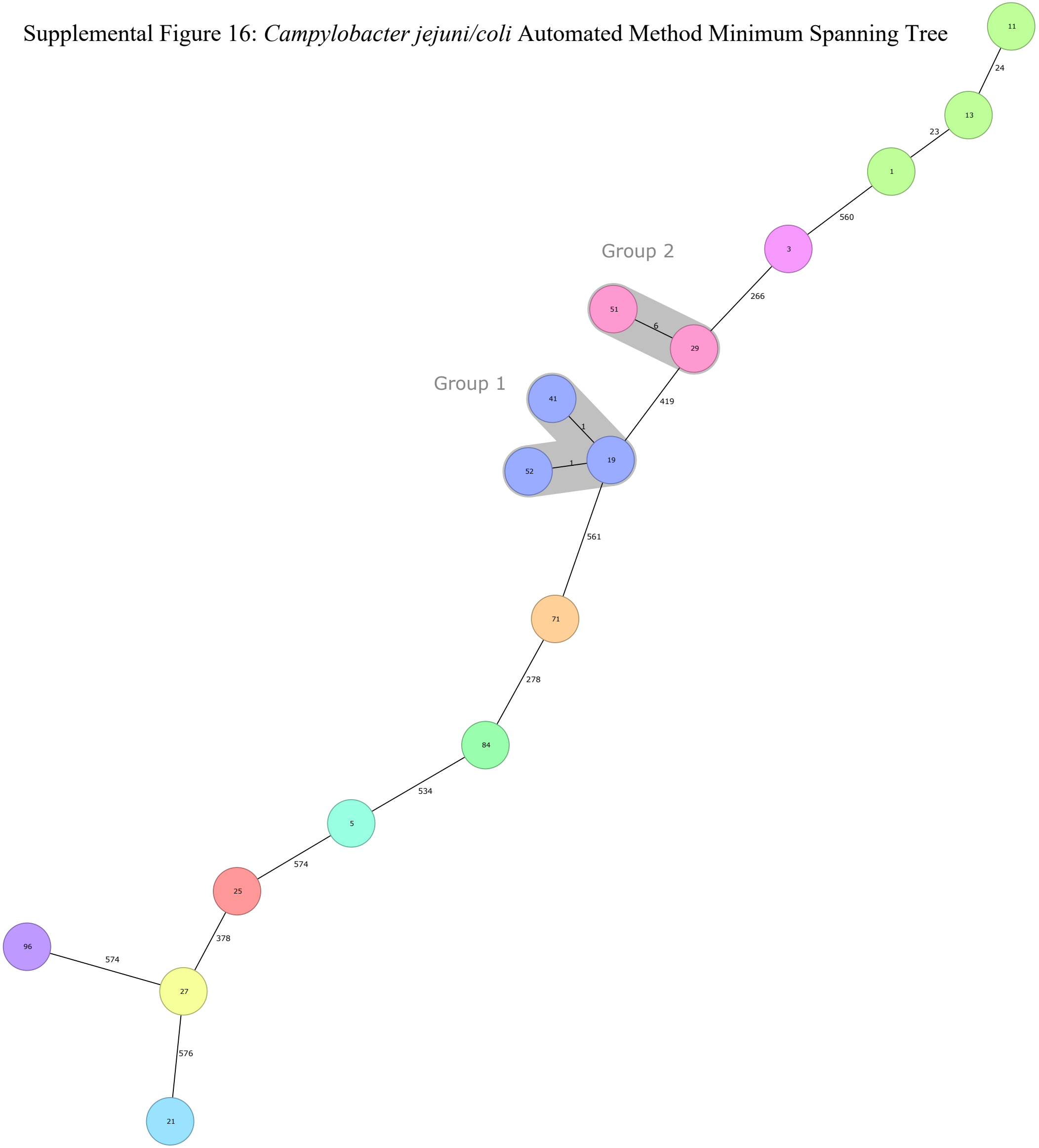

Supplemental Figure 17: *Enterococcus faecium* Manual Method Minimum Spanning Tree

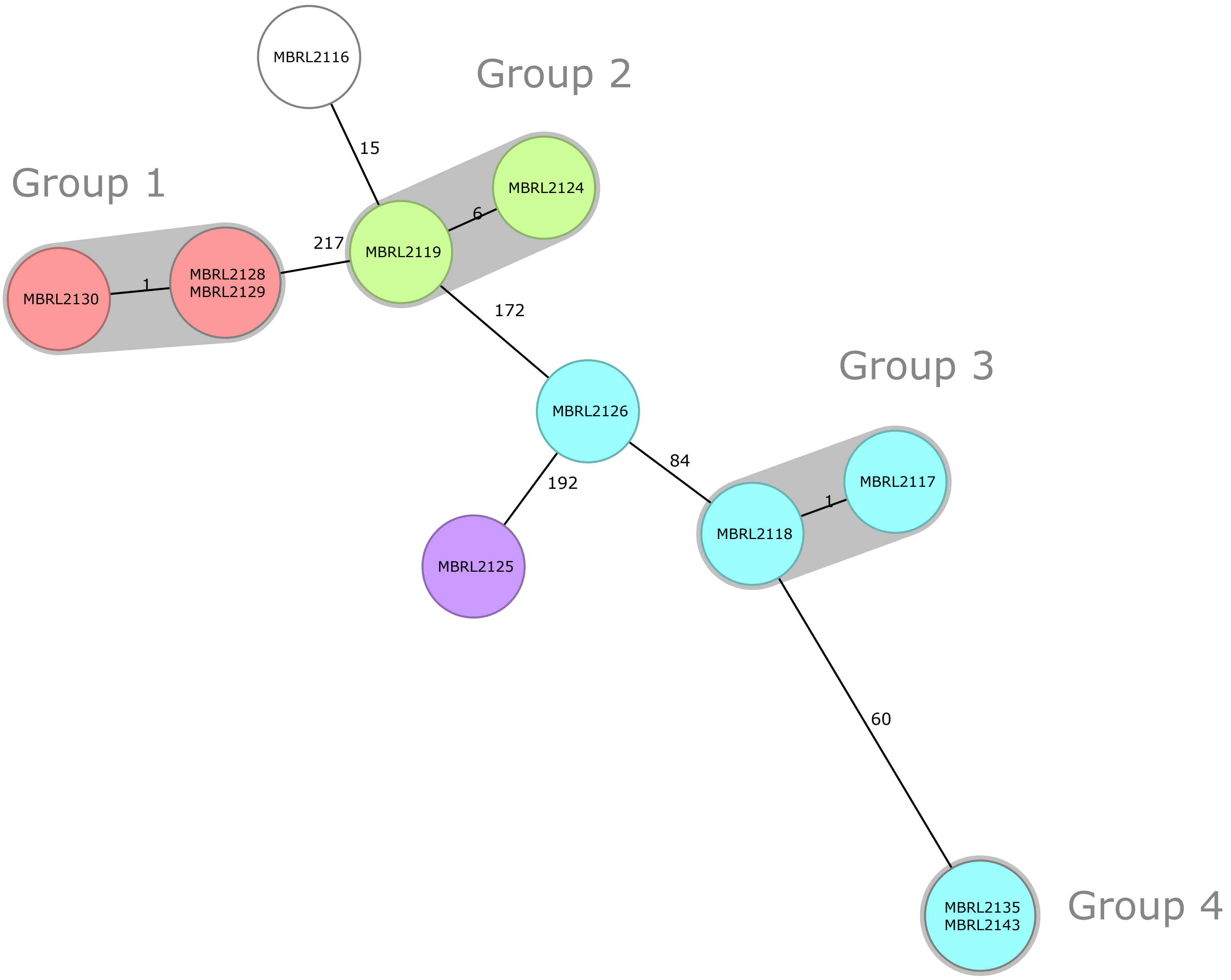

Supplemental Figure 18: *Enterococcus faecium* Automated Method Minimum Spanning Tree

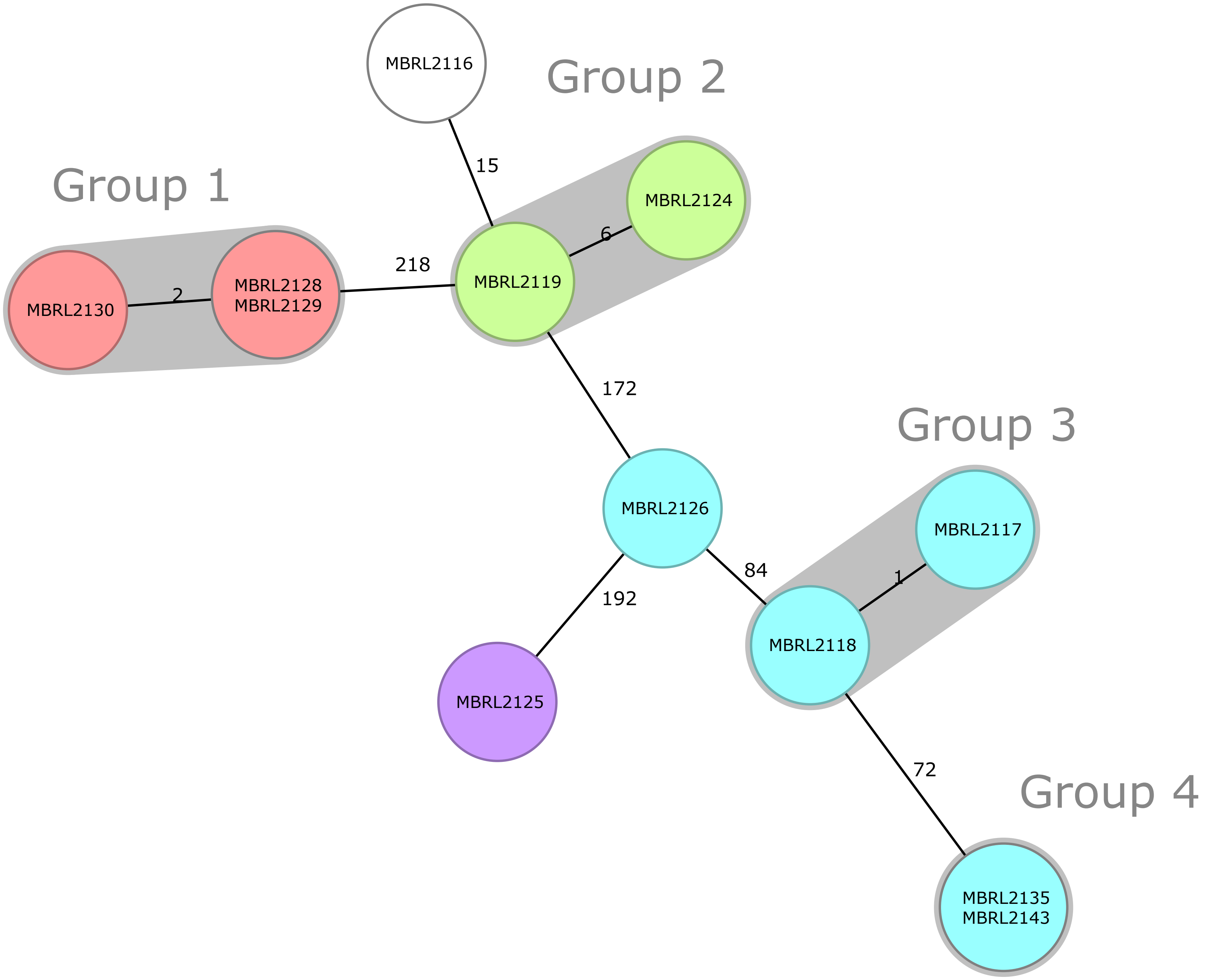

Supplemental Figure 19: *Enterococcus faecalis* Manual Method Minimum Spanning Tree

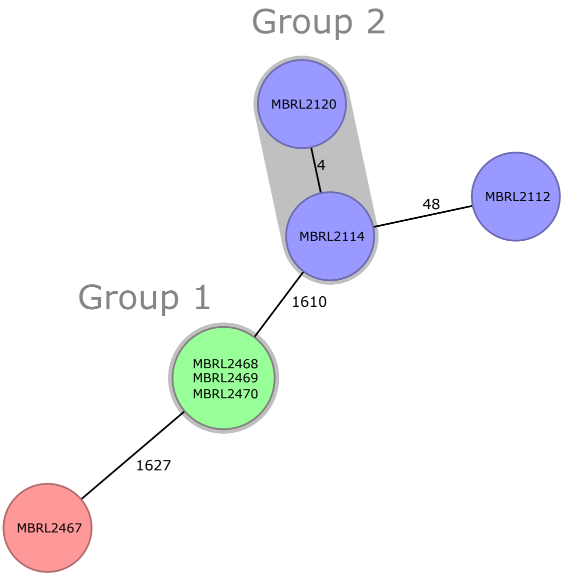

Supplemental Figure 20: *Enterococcus faecalis* Automated Method Minimum Spanning Tree

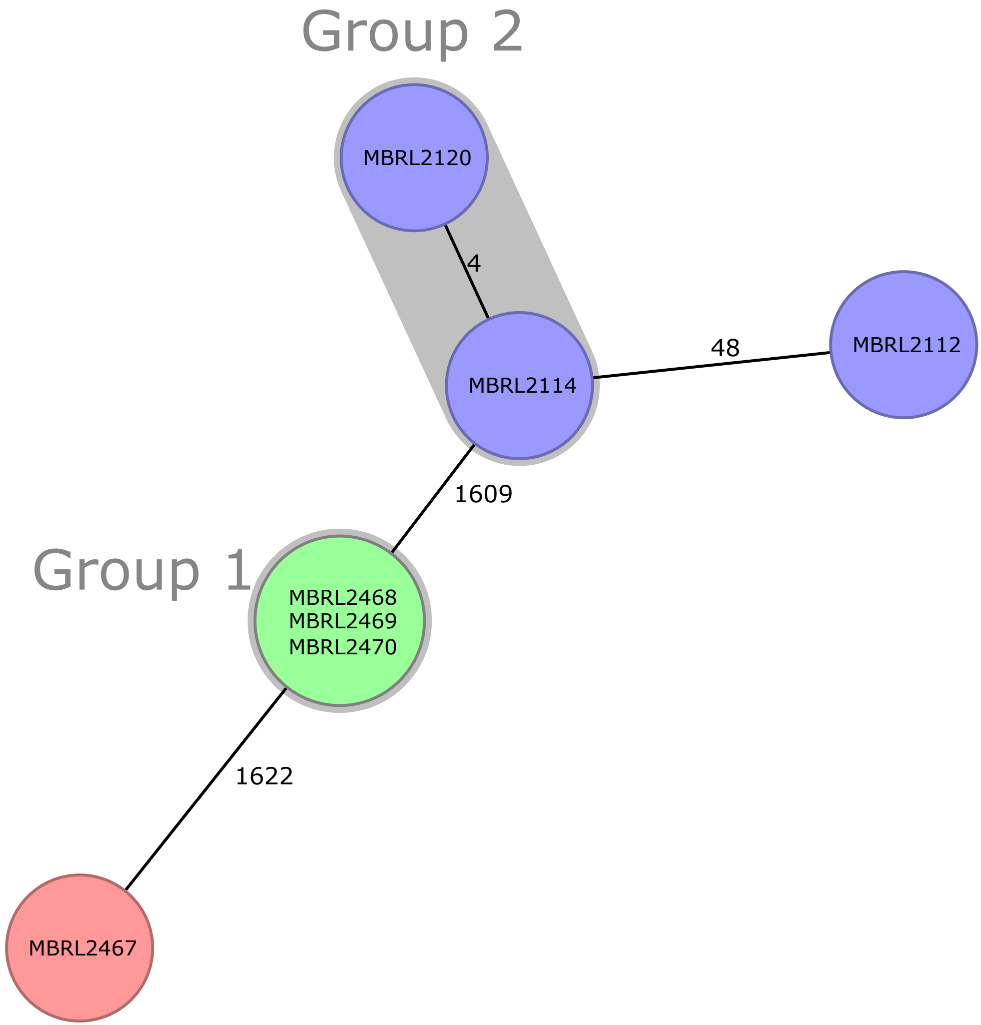

Supplemental Figure 21: *Streptococcus pyogenes* Manual Method Minimum Spanning Tree

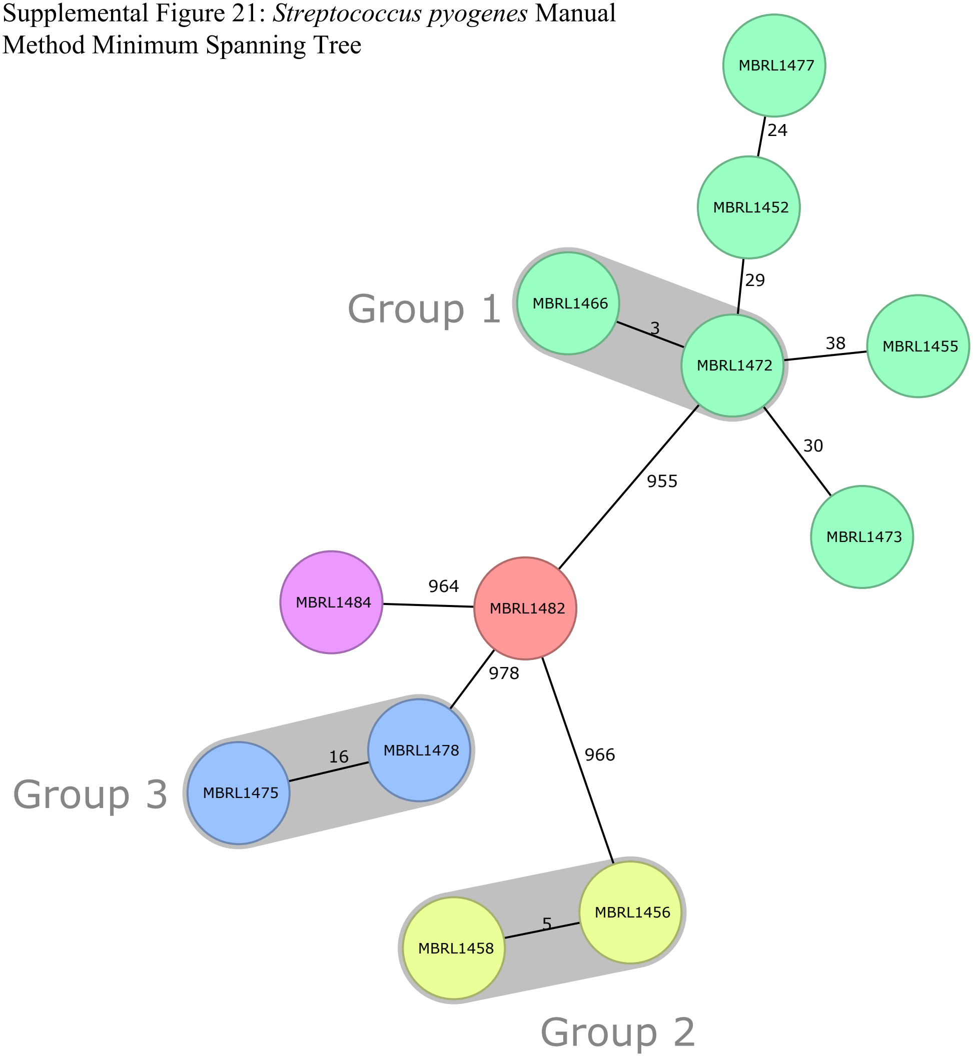

Supplemental Figure 22: *Streptococcus pyogenes* Automated Method Minimum Spanning Tree

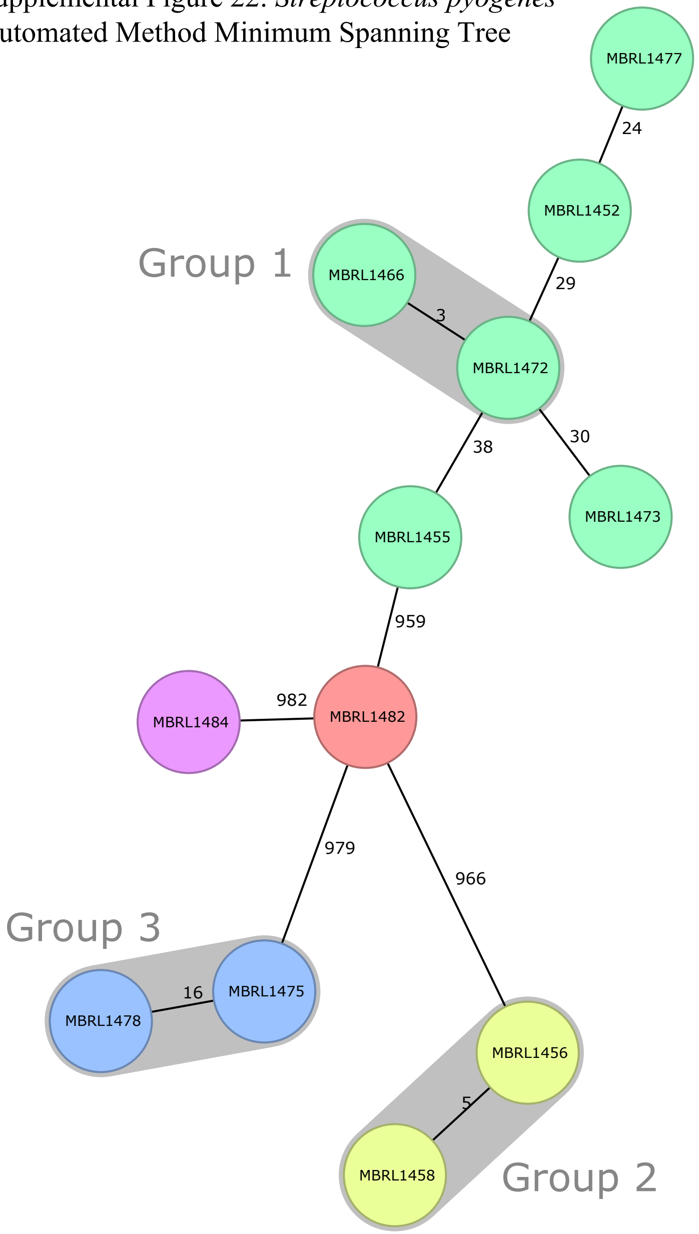

Supplemental Figure 23: *Serratia marcescens* Manual Method Minimum Spanning Tree

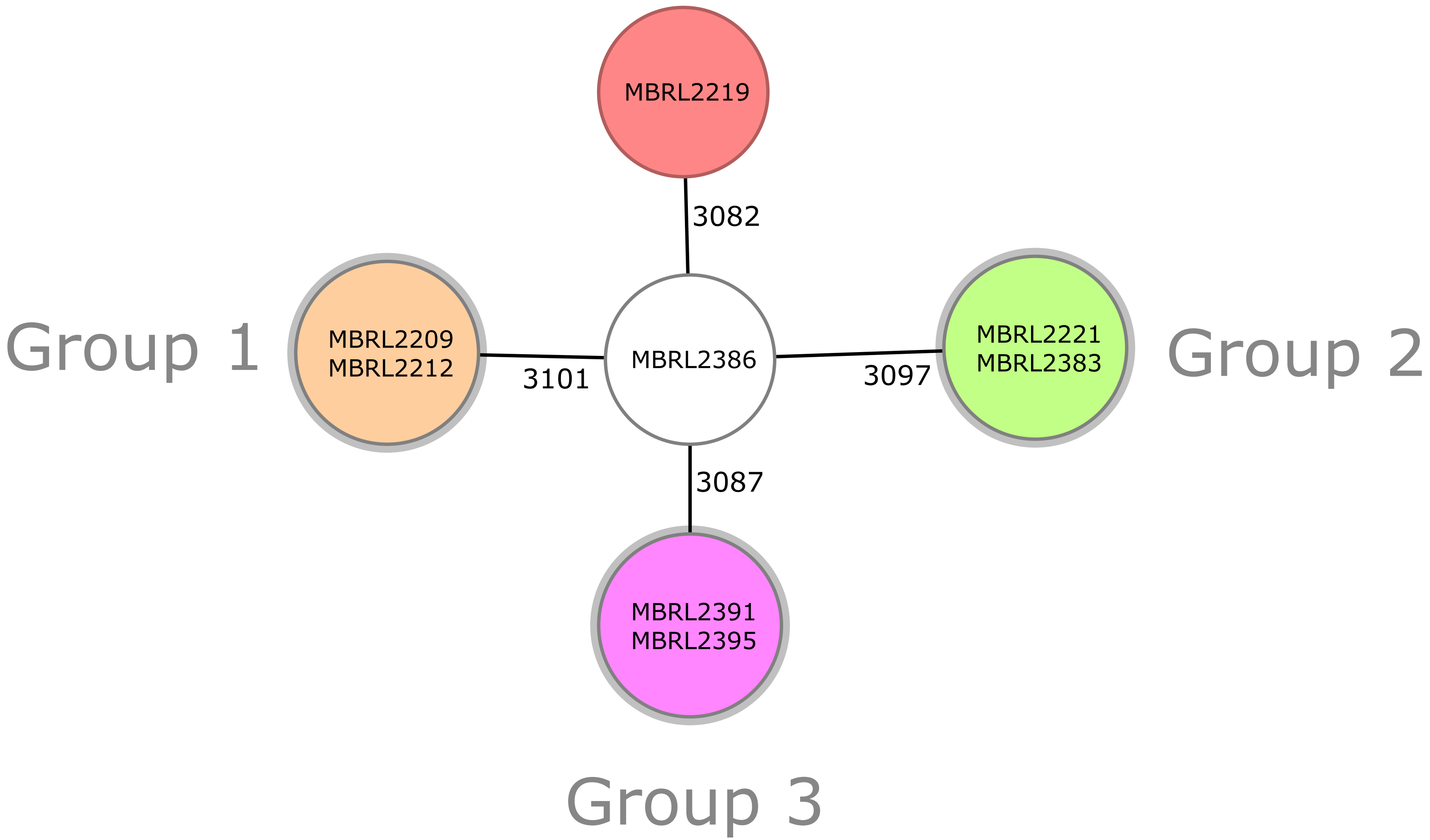

Supplemental Figure 24: *Serratia marcescens* Automated Method Minimum Spanning Tree

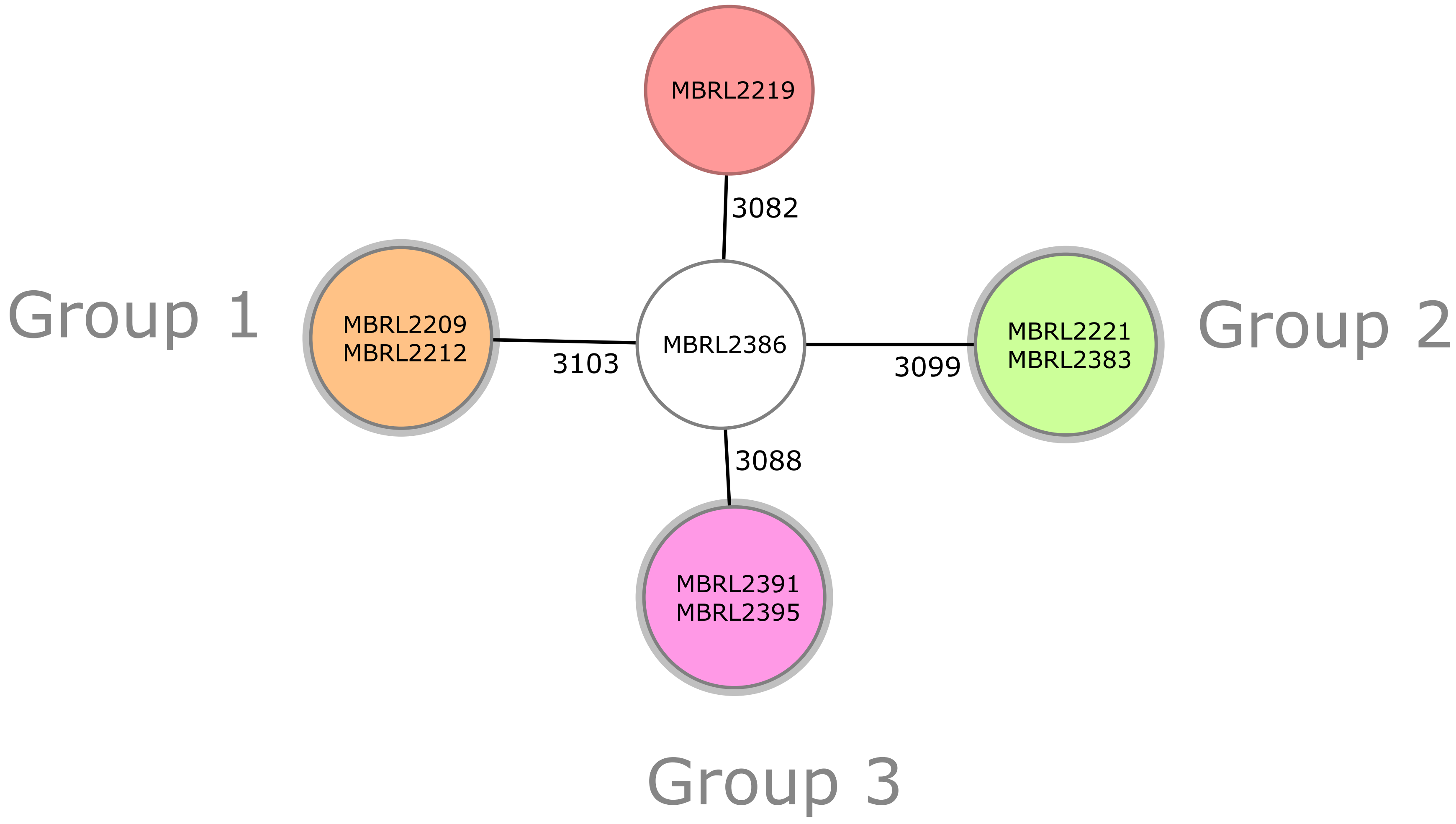

Supplemental Figure 25: *Pseudomonas aeruginosa* Manual Method Minimum Spanning Tree

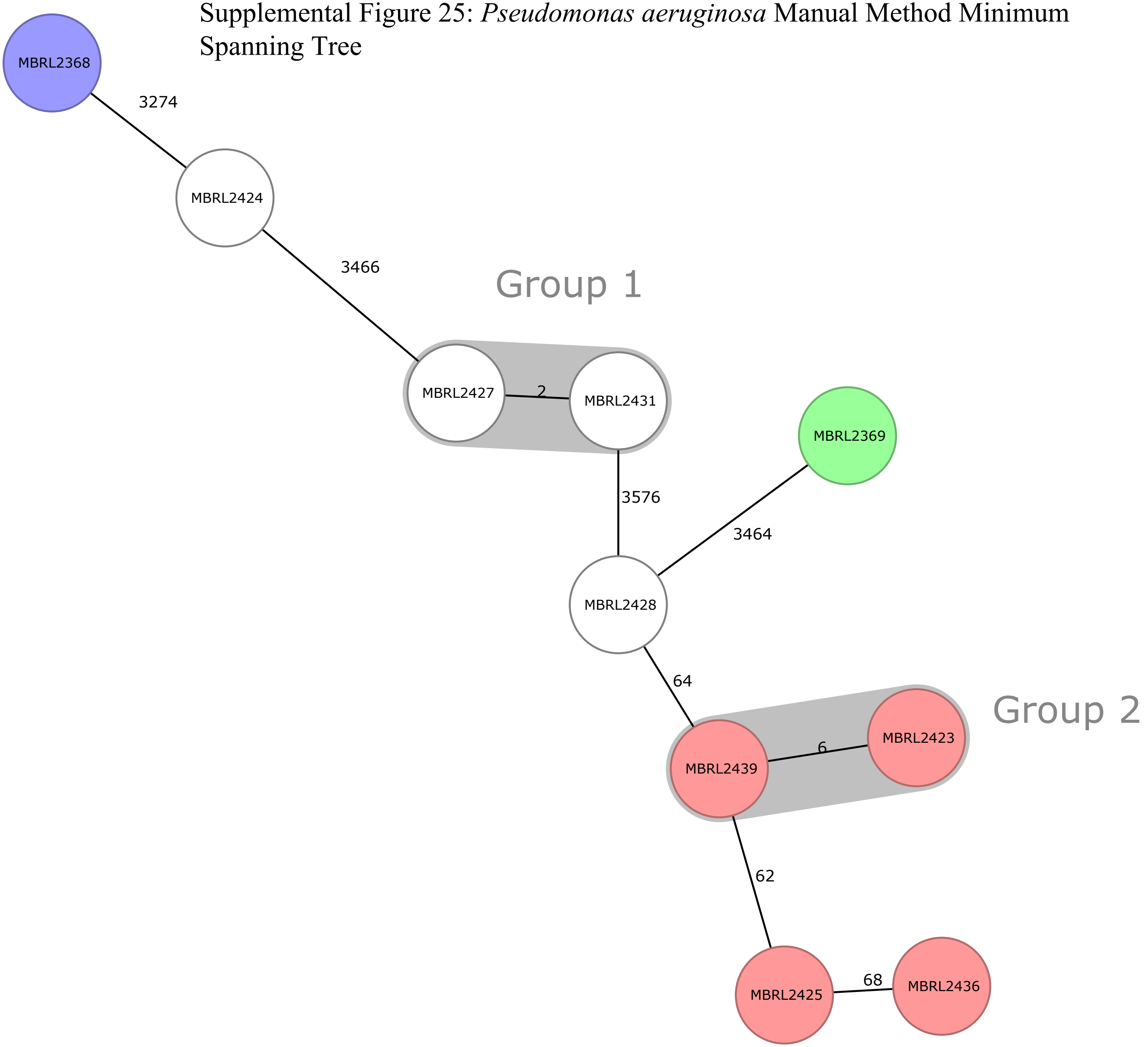

Supplemental Figure 26: *Pseudomonas aeruginosa* Automated Method Minimum Spanning Tree

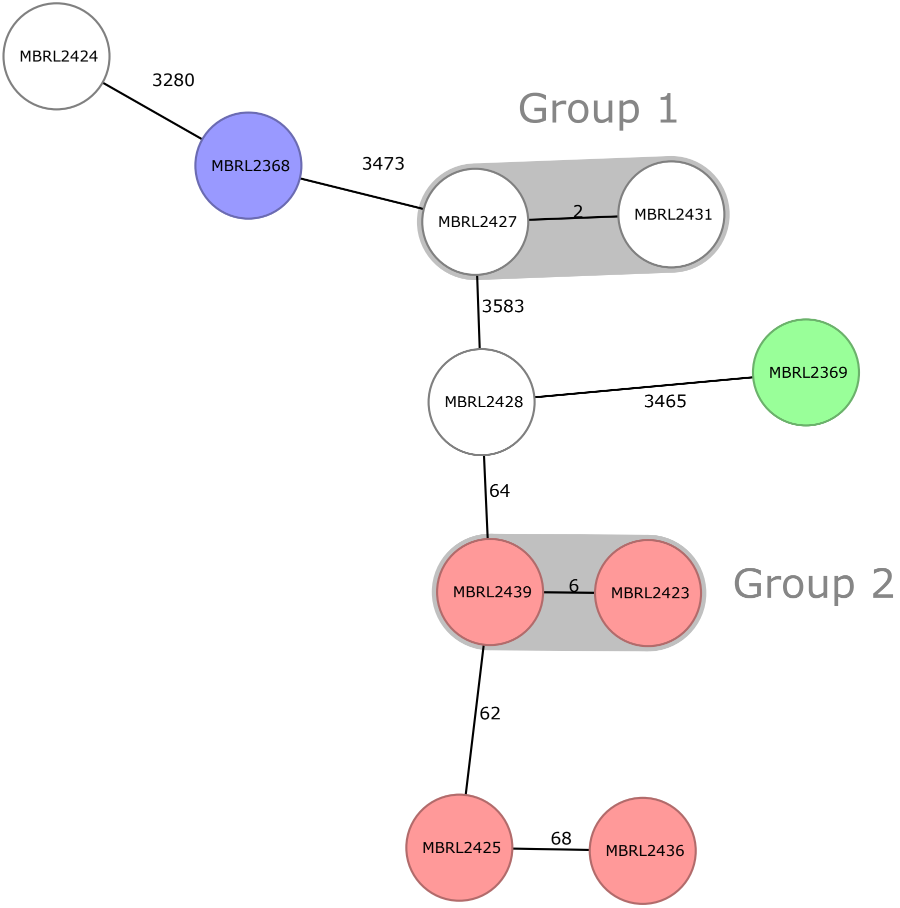

Supplemental Figure 27: *Streptococcus agalactiae* Manual Method Minimum Spanning Tree

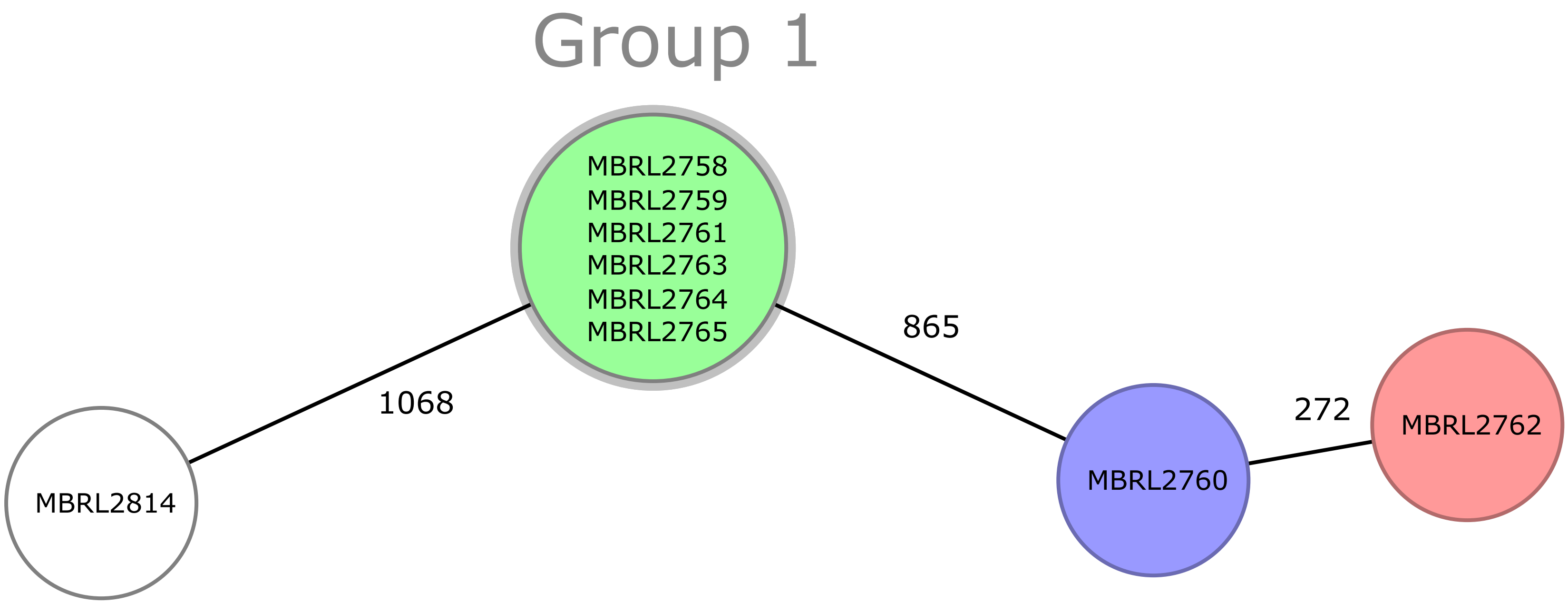

Supplemental Figure 28: *Streptococcus agalactiae* Automated Method Minimum Spanning Tree

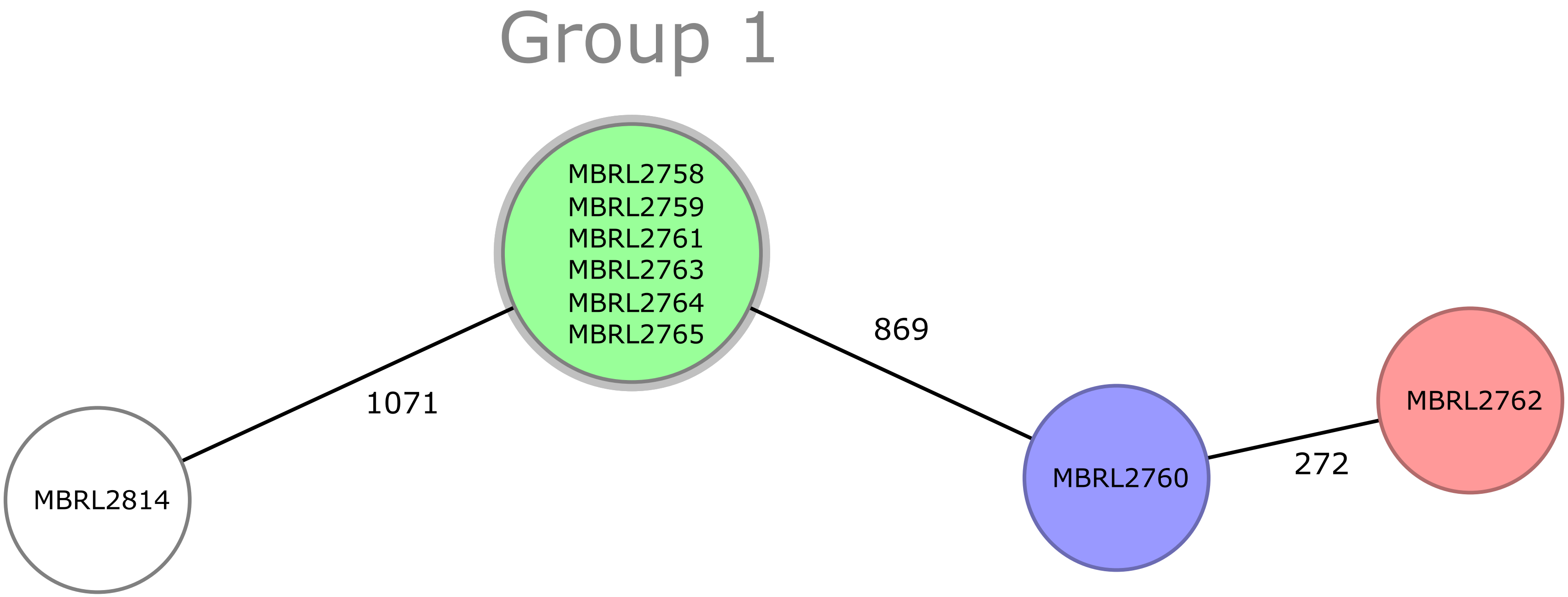

Supplemental Figure 29: *Staphylococcus lugdunensis* Manual Method Minimum Spanning Tree

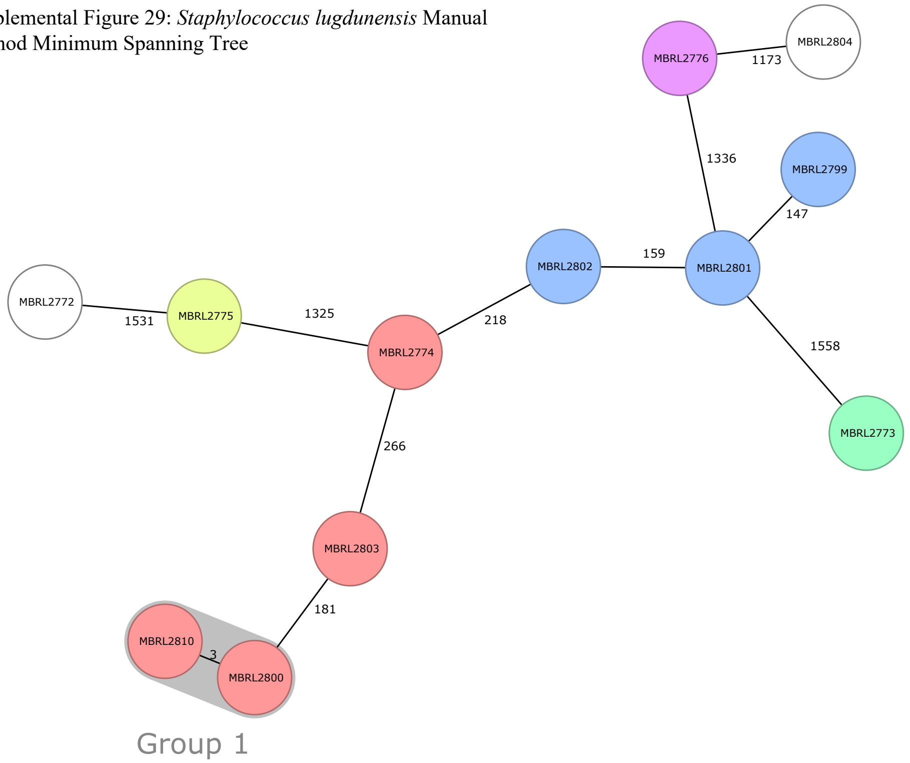

Supplemental Figure 30: *Staphylococcus lugdunensis* Automated Method Minimum Spanning Tree

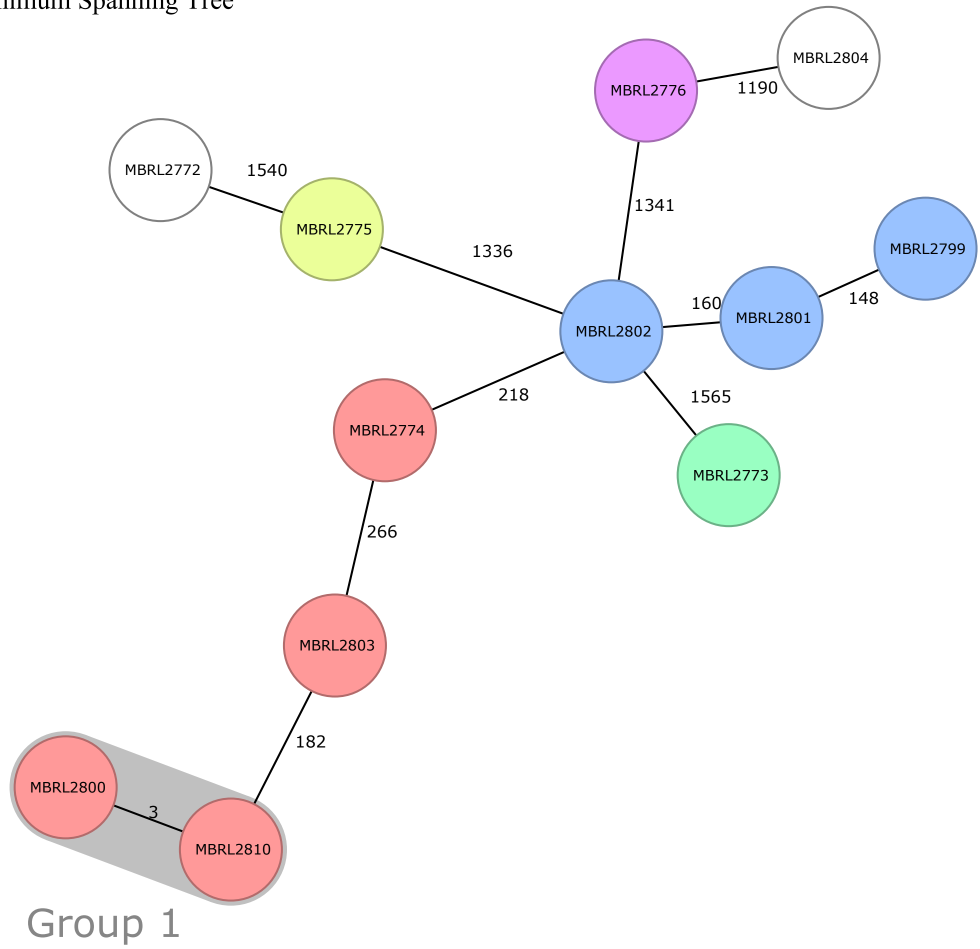

Supplemental Figure 31: *Staphylococcus epidermidis* Manual Method Minimum Spanning Tree

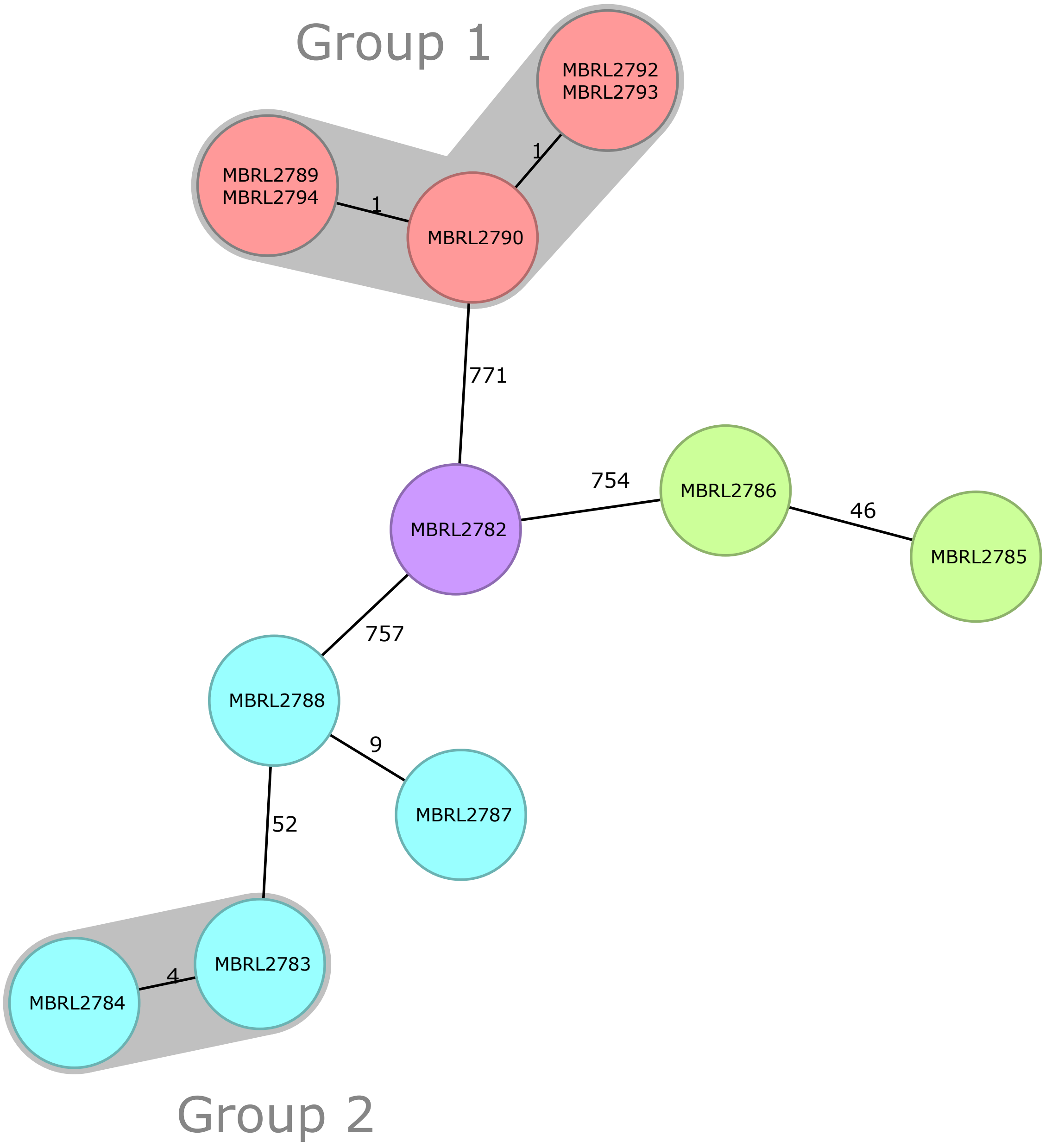

Supplemental Figure 32: *Staphylococcus epidermidis* Automated Method Minimum Spanning Tree

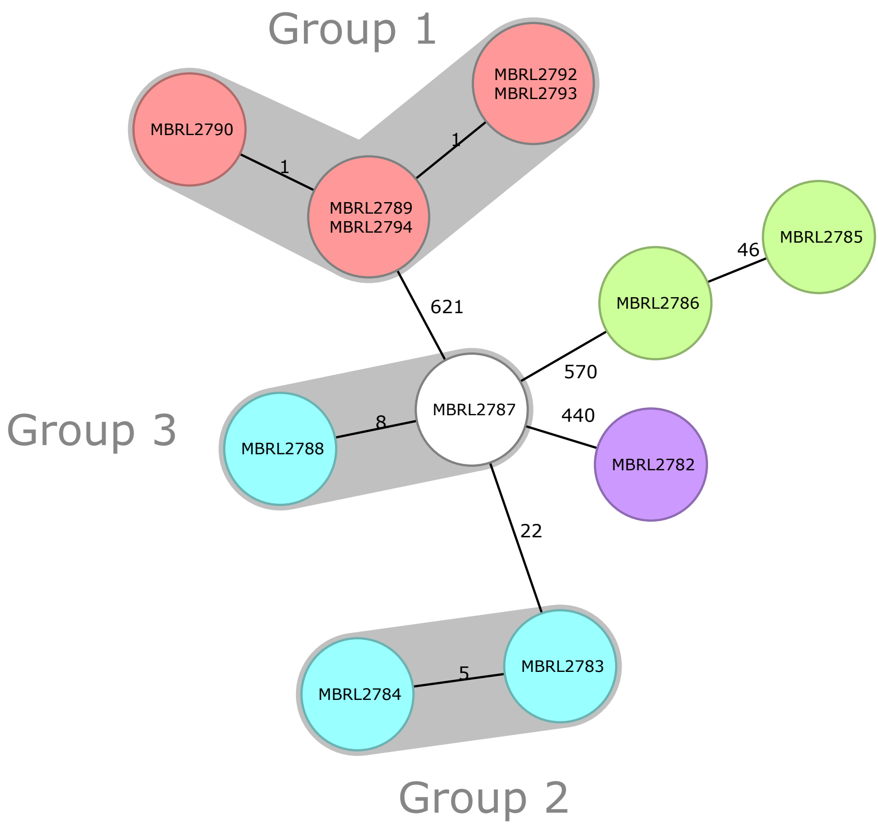

Supplemental Figure 33: *Cutibacterium acnes* Manual Method Minimum Spanning Tree

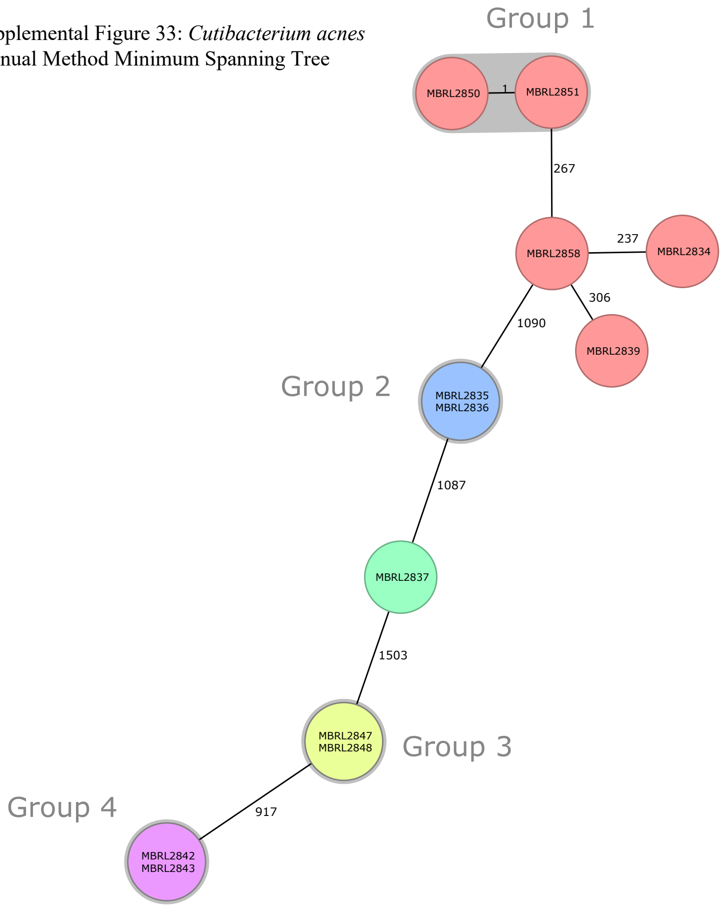

Supplemental Figure 34: *Cutibacterium acnes* Automated Method Minimum Spanning Tree

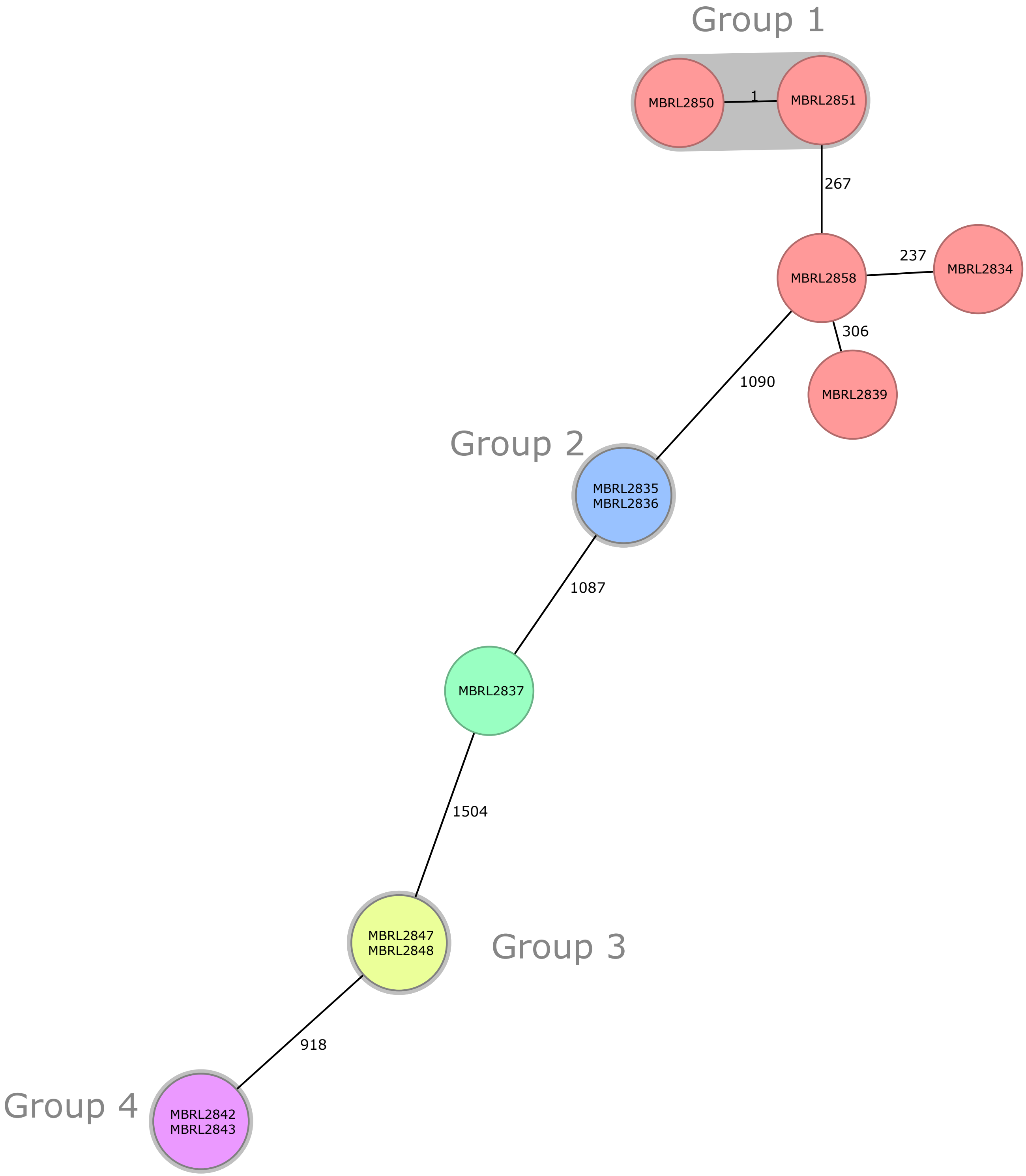

Supplemental Table 1: Study Isolates

Organism	Specimen Identification	Relatedness Category	Source
Staphylococcus aureus	BTP[a] 197	Group 1	2014-2017 Mayo Clinic neonatal intensive care unit methicillin-resistant *Staphylococcus aureus* outbreak collection
	BTP 198	Group 2	
	BTP 199	Group 1	
	BTP 200	Group 1	
	BTP 201	Group 1	
	BTP 202	Group 1	
	BTP 203	Group 1	
	BTP 204	Group 1	
	BTP 205	Group 1	
	BTP 206	No group	
	BTP 207	Group 1	
	BTP 208	No group	
	BTP 209	No group	
	BTP 210	Group 1	
	BTP 211	Group 1	
	BTP 212	Group 2	
	BTP 213	No group	
	BTP 214	No group	
	BTP 215	No group	
	BTP 216	No group	
	BTP 217	Group 4	
	BTP 218	Group 4	
	BTP 219	Group 2	
	BTP 220	Group 2	
	BTP 221	Group 2	
	BTP 222	Group 2	
	BTP 223	Group 2	
	BTP 224	Group 3	
	BTP 225	Group 2	
	BTP 226	Group 1	
	BTP 227	Group 1	
	BTP 228	Group 2	
	BTP 229	Group 2	
	BTP 230	Group 2	
	BTP 231	Group 1	
	BTP 232	Group 1	
	BTP 233	Group 2	
	BTP 234	Group 1	

	BTP 235	Group 1	
	BTP 236	Group 1	
	BTP 237	Group 2	
	BTP 238	Group 2	
	BTP 239	Group 2	
	BTP 240	Group 2	
	BTP 241	Group 2	
	BTP 242	Group 2	
	BTP 243	Group 1	
	BTP 244	Group 2	
	BTP 245	Group 1	
	BTP 246	Not included in analysis[1]	
	BTP 247	Group 2	
	BTP 248	Group 1	
	BTP 249	Group 1	
	BTP 250	No group	
	BTP 251	Group 3	
	BTP 252	No group	
	BTP 253	Group 1	
	BTP 254	Group 2	
	BTP 255	No group	
	BTP 256	Group 2	
	BTP 257	Group 1	
	BTP 258	Group 2	
	USA100	No group	Pulsed-field gel electrophoresis type strains
	USA200	Not included in analysis[2]	
	USA300	No group	
	USA400	No group	
	USA500	No group	
	USA600	No group	
	USA700	No group	
	USA800	No group	
	USA900	No group	
	USA1000	No group	
Acinetobacter baumannii	ARLG[b]-1256	Group 3	Antibacterial Resistance Leadership Group
	ARLG-1257	Possibly related to Groups 2 and 3	
	ARLG-1262	Group 2	
	ARLG-1263	Group 2	

	ARLG-1264	Possibly related to Group 1	
	ARLG-1266	Group 3	
	ARLG-1275	Group 1	
	ARLG-1278	Group 1	
Klebsiella pneumoniae	ARLG-1159	Possibly related to Group 1	
	ARLG-1163	Possibly related to Group 1 and ARLG-1168	
	ARLG-1164	No group	
	ARLG-1167	Group 1	
	ARLG-1168	Possibly related to ARLG-1163	
	ARLG-1169	No group	
	ARLG-1719	Group 1	
	ARLG-1720	Group 1	
Legionella pneumophila	MBRL[c] 1495	Group 2	Minnesota Department of Health
	MBRL 1496	Group 1	
	MBRL 1497	Group 1	
	MBRL 1498	No group	
	MBRL 1499	No group	
	MBRL 1504	Possibly related to Group 2	
	MBRL 1508	Group 2	
Clostridioides difficile	28814	Group 1	Mayo Clinic Bacteriology Laboratory
	29666	No group	
	29770	No group	
	30503	Group 1	
	30506	No group	
	30516	Group 2	
	30533	No group	
	30535	Group 2	
Escherichia coli	MBRL 2159	Group 2	
	MBRL 2160	Group 2	
	MBRL 2378	No group	
	MBRL 2409	Group 1	
	MBRL 2410	Group 1	
	MBRL 2411	Group 1	
	MBRL 2412	Group 1	
	MBRL 2461	No group	
	MBRL 2471	Group 2	

Enterobacter cloacae complex		MBRL 2472	Group 1	
		MBRL 2473	Group 1	
		MBRL 2474	Group 2	
Campylobacter jejuni/coli	*C. jejuni*	1	Possibly related to isolate 13	Mayo Clinic Gastrointestinal Barrier Function Laboratory
	C. jejuni	3	No group	
	C. jejuni	5	No group	
	C. jejuni	11	Possibly related to isolate 13	
	C. jejuni	13	Possibly related to isolates 1 and 11	
	C. jejuni	19	Group 1	
	C. jejuni	21	No group	
	C. coli	25	No group	
	C. coli	27	No group	
	C. jejuni	29	Group 2	
	C. jejuni	41	Group 1	
	C. jejuni	51	Group 2	
	C. jejuni	52	Group 1	
	C. jejuni	71	No group	
	C. jejuni	84	No group	
	C. jejuni	96	No group	
Enterococcus faecium		MBRL 2116	Possibly related to Group 2	Mayo Clinic Infectious Diseases Research Laboratory
		MBRL 2117	Group 3	
		MBRL 2118	Group 3	
		MBRL 2119	Group 2	
		MBRL 2124	Group 2	
		MBRL 2125	No group	
		MBRL 2126	No group	
		MBRL 2128	Group 1	
		MBRL 2129	Group 1	
		MBRL 2130	Group 1	
		MBRL 2135	Group 4	
		MBRL 2143	Group 4	
Enterococcus faecalis		MBRL 2112	No group	
		MBRL 2114	Group 2	
		MBRL 2120	Group 2	
		MBRL 2467	No group	Mayo Clinic Bacteriology Laboratory
		MBRL 2468	Group 1	
		MBRL 2469	Group 1	
		MBRL 2470	Group 1	

Streptococcus pyogenes	MBRL 1452	Possibly related to Group 1 and MBRL 1477	Minnesota Department of Health
	MBRL 1455	PR-Group 1	
	MBRL 1456	Group 2	
	MBRL 1458	Group 2	
	MBRL 1466	Group 1	
	MBRL 1472	Group 1	
	MBRL 1473	Possibly related to Group 1	
	MBRL 1475	Group 3	
	MBRL 1477	Possibly related to MBRL 1452	
	MBRL 1478	Group 3	
	MBRL 1482	No group	
	MBRL 1484	No group	
Serratia marcescens	MBRL 2209	Group 1	Mayo Clinic Bacteriology Laboratory
	MBRL 2212	Group 1	
	MBRL 2219	No group	
	MBRL 2221	Group 2	
	MBRL 2383	Group 2	
	MBRL 2386	No group	
	MBRL 2391	Group 3	
	MBRL 2395	Group 3	
Pseudomonas aeruginosa	MBRL 2368	No group	
	MBRL 2369	No group	
	MBRL 2423	Group 2	
	MBRL 2424	No group	
	MBRL 2425	Possibly related to Group 2 and MBRL 2436	
	MBRL 2427	Group 1	
	MBRL 2428	Possibly related to Group 2	
	MBRL 2431	Group 1	
	MBRL 2436	Possibly related to MBRL 2425	
	MBRL 2439	Group 2	
Streptococcus agalactiae	MBRL 2758	Group 1	
	MBRL 2759	Group 1	
	MBRL 2760	No group	
	MBRL 2761	Group 1	
	MBRL 2762	No group	

	MBRL 2763	Group 1	
	MBRL 2764	Group 1	
	MBRL 2765	Group 1	
	MBRL 2814	No group	ATCC[d] 13813
Staphylococcus lugdunensis	MBRL 2772	No group	Mayo Clinic Bacteriology Laboratory
	MBRL 2773	No group	
	MBRL 2774	No group	
	MBRL 2775	No group	
	MBRL 2776	No group	
	MBRL 2799	No group	Mayo Clinic Infectious Diseases Research Laboratory
	MBRL 2800	Group 1	
	MBRL 2801	No group	
	MBRL 2802	No group	
	MBRL 2803	No group	
	MBRL 2804	No group	
	MBRL 2810	Group 1	
Staphylococcus epidermidis	MBRL 2782	No group	ATCC 14990
	MBRL 2783	Group 2	Mayo Clinic Infectious Diseases Research Laboratory
	MBRL 2784	Group 2	
	MBRL 2785	No group	
	MBRL 2786	No group	
	MBRL 2787	Manual method: Possibly related to MBRL 2788 Automated method: Group 3	
	MBRL 2788	Manual method: Possibly related to MBRL 2787 Automated method: Group 3	
	MBRL 2789	Group 1	
	MBRL 2790	Group 1	
	MBRL 2792	Group 1	
	MBRL 2793	Group 1	
	MBRL 2794	Group 1	
Cutibacterium acnes	MBRL 2834	No group	Dr. Faten El Sayed, Assistance Publique-Hôpitaux de Paris, Université Paris Saclay
	MBRL 2835	Group 2	
	MBRL 2836	Group 2	
	MBRL 2837	No group	
	MBRL 2839	No group	

	MBRL 2842	Group 4	
	MBRL 2843	Group 4	
	MBRL 2847	Group 3	
	MBRL 2848	Group 3	
	MBRL 2850	Group 1	
	MBRL 2851	Group 1	
	MBRL 2858	No group	

[a]BTP, Bacterial typing project

[b]ARLG, Antibacterial Resistance Leadership Group

[c]MBRL, Mayo Bacteriology Research Laboratory

[d]ATCC, American Type Culture Collection

[1]Excluded from analysis due to a pipetting error on the Clear Dx™ instrument that resulted in no sequence generated.

[2]Excluded from analysis due to contamination with another organism upon subculture.

Supplemental Table 2: *Staphylococcus aureus* Allelic Differences Between Isolates

Manual WGS Method

Automated WGS Method

Isolate	BTP 197	BTP 198	BTP 199	BTP 200	BTP 201	BTP 202	BTP 203	BTP 204	BTP 205	BTP 206	BTP 207	BTP 208	BTP 209	BTP 210	BTP 211	BTP 212	BTP 213	BTP 214	BTP 215	BTP 216	BTP 217	BTP 218	BTP 219	BTP 220
BTP 197	0 / 0	1488 / 1489	1 / 1	1 / 1	0 / 0	0 / 0	1 / 1	0 / 0	1 / 1	1485 / 1486	1 / 1	1487 / 1487	1486 / 1487	2 / 1	1 / 1	1487 / 1488	1488 / 1488	1709 / 1709	1370 / 1370	1711 / 1710	1481 / 1481	1479 / 1481	1488 / 1489	1490 / 1490
BTP 198	1488 / 1489	0 / 0	1488 / 1489	1487 / 1488	1488 / 1489	1488 / 1488	1487 / 1489	1486 / 1488	1488 / 1488	235 / 235	1487 / 1488	284 / 284	105 / 105	1487 / 1487	1488 / 1489	12 / 12	64 / 64	1706 / 1707	1467 / 1467	1707 / 1707	110 / 110	113 / 113	4 / 4	4 / 4
BTP 199	1 / 1	1488 / 1489	0 / 0	2 / 2	1 / 1	1 / 1	2 / 2	1 / 1	2 / 2	1485 / 1486	2 / 2	1487 / 1487	1486 / 1487	3 / 2	2 / 2	1487 / 1488	1488 / 1488	1709 / 1709	1370 / 1370	1711 / 1710	1481 / 1481	1479 / 1481	1488 / 1489	1490 / 1490
BTP 200	1 / 1	1487 / 1488	2 / 2	0 / 0	1 / 1	1 / 1	2 / 2	1 / 1	0 / 0	1485 / 1485	2 / 2	1486 / 1486	1486 / 1486	1 / 0	2 / 2	1486 / 1487	1487 / 1487	1708 / 1708	1369 / 1370	1710 / 1709	1480 / 1480	1478 / 1480	1487 / 1488	1489 / 1489
BTP 201	0 / 0	1488 / 1489	1 / 1	1 / 1	0 / 0	0 / 0	1 / 1	0 / 0	1 / 1	1485 / 1486	1 / 1	1487 / 1487	1486 / 1487	2 / 1	1 / 1	1487 / 1488	1488 / 1488	1709 / 1709	1370 / 1370	1711 / 1710	1481 / 1481	1479 / 1481	1488 / 1489	1490 / 1490
BTP 202	0 / 0	1488 / 1488	1 / 1	1 / 1	0 / 0	0 / 0	1 / 1	0 / 0	1 / 1	1485 / 1485	1 / 1	1487 / 1486	1486 / 1486	2 / 1	1 / 1	1487 / 1487	1488 / 1487	1709 / 1708	1370 / 1369	1711 / 1709	1481 / 1480	1479 / 1480	1488 / 1488	1490 / 1489
BTP 203	1 / 1	1487 / 1489	2 / 2	2 / 2	1 / 1	1 / 1	0 / 0	1 / 1	2 / 2	1484 / 1486	2 / 2	1486 / 1487	1485 / 1487	3 / 2	2 / 2	1486 / 1488	1487 / 1488	1708 / 1709	1369 / 1370	1710 / 1710	1480 / 1481	1478 / 1481	1487 / 1489	1489 / 1490
BTP 204	1 / 1	1486 / 1488	1 / 1	1 / 1	0 / 0	0 / 0	1 / 1	0 / 0	1 / 1	1483 / 1485	1 / 1	1485 / 1486	1484 / 1486	2 / 1	1 / 1	1485 / 1487	1486 / 1487	1706 / 1708	1367 / 1370	1708 / 1709	1479 / 1480	1477 / 1480	1486 / 1488	1488 / 1489
BTP 205	1 / 1	1488 / 1488	2 / 2	0 / 0	1 / 1	1 / 1	2 / 2	1 / 1	0 / 0	1485 / 1485	2 / 2	1487 / 1486	1486 / 1486	1 / 0	2 / 2	1487 / 1487	1488 / 1487	1709 / 1708	1370 / 1370	1711 / 1709	1481 / 1480	1479 / 1480	1488 / 1488	1490 / 1489
BTP 206	1485 / 1486	235 / 235	1485 / 1486	1485 / 1485	1485 / 1486	1485 / 1485	1484 / 1486	1483 / 1485	1485 / 1485	0 / 0	1484 / 1485	255 / 255	232 / 232	1484 / 1484	1485 / 1486	238 / 238	228 / 228	1703 / 1705	1471 / 1471	1703 / 1704	228 / 228	231 / 231	233 / 233	232 / 232
BTP 207	1 / 1	1487 / 1488	2 / 2	2 / 2	1 / 1	1 / 1	2 / 2	1 / 1	2 / 2	1484 / 1485	0 / 0	1486 / 1486	1485 / 1486	3 / 2	2 / 2	1486 / 1487	1487 / 1487	1709 / 1709	1369 / 1369	1711 / 1710	1480 / 1480	1478 / 1480	1487 / 1488	1489 / 1489
BTP 208	1487 / 1488	284 / 284	1487 / 1487	1486 / 1486	1487 / 1487	1487 / 1486	1486 / 1487	1485 / 1486	1487 / 1486	255 / 255	1486 / 1486	0 / 0	282 / 282	1486 / 1485	1487 / 1487	287 / 287	273 / 273	1706 / 1706	1465 / 1464	1707 / 1706	284 / 284	286 / 286	283 / 283	281 / 281
BTP 209	1487 / 1488	105 / 105	1486 / 1487	1486 / 1486	1486 / 1487	1487 / 1486	1485 / 1487	1484 / 1486	1486 / 1486	232 / 232	1485 / 1486	282 / 282	0 / 0	1485 / 1485	1486 / 1487	109 / 109	98 / 98	1701 / 1703	1463 / 1464	1702 / 1703	93 / 93	95 / 95	104 / 104	102 / 102
BTP 210	2 / 1	1487 / 1487	3 / 2	1 / 0	2 / 1	2 / 1	3 / 2	2 / 1	1 / 0	1484 / 1484	3 / 2	1486 / 1485	1485 / 1485	0 / 0	3 / 2	1486 / 1486	1487 / 1486	1707 / 1706	1369 / 1368	1709 / 1707	1480 / 1479	1478 / 1479	1487 / 1487	1489 / 1488
BTP 211	1 / 1	1488 / 1489	2 / 2	2 / 2	1 / 1	1 / 1	2 / 2	1 / 1	2 / 2	1485 / 1486	2 / 2	1487 / 1487	1486 / 1487	3 / 2	0 / 0	1487 / 1488	1488 / 1488	1709 / 1709	1370 / 1370	1711 / 1710	1481 / 1481	1479 / 1481	1488 / 1489	1490 / 1490
BTP 212	1487 / 1488	12 / 12	1487 / 1488	1486 / 1487	1487 / 1488	1487 / 1487	1486 / 1488	1485 / 1487	1487 / 1487	238 / 238	1486 / 1487	287 / 287	109 / 109	1486 / 1486	1487 / 1488	0 / 0	69 / 69	1704 / 1706	1465 / 1466	1705 / 1706	114 / 114	117 / 117	10 / 10	8 / 8
BTP 213	1488 / 1488	64 / 64	1488 / 1488	1487 / 1487	1488 / 1488	1488 / 1487	1487 / 1488	1486 / 1487	1488 / 1487	228 / 228	1487 / 1487	273 / 273	98 / 98	1487 / 1486	1488 / 1488	69 / 69	0 / 0	1706 / 1706	1467 / 1466	1707 / 1706	105 / 105	108 / 108	63 / 63	61 / 61
BTP 214	1709 / 1709	1706 / 1707	1709 / 1709	1708 / 1708	1709 / 1709	1709 / 1708	1708 / 1709	1706 / 1708	1709 / 1708	1703 / 1705	1709 / 1709	1706 / 1706	1701 / 1703	1707 / 1706	1709 / 1709	1704 / 1706	1706 / 1706	0 / 0	1705 / 1704	203 / 203	1702 / 1702	1701 / 1702	1706 / 1707	1708 / 1708
BTP 215	1370 / 1370	1467 / 1467	1370 / 1370	1369 / 1370	1370 / 1370	1370 / 1369	1369 / 1370	1367 / 1370	1370 / 1370	1471 / 1471	1369 / 1369	1465 / 1464	1463 / 1464	1369 / 1368	1370 / 1370	1465 / 1466	1467 / 1466	1705 / 1704	0 / 0	1705 / 1703	1463 / 1462	1462 / 1462	1466 / 1466	1468 / 1467
BTP 216	1711 / 1710	1707 / 1707	1711 / 1710	1710 / 1709	1711 / 1710	1711 / 1709	1710 / 1710	1708 / 1709	1711 / 1709	1703 / 1704	1711 / 1710	1707 / 1706	1702 / 1703	1709 / 1707	1711 / 1710	1705 / 1706	1707 / 1706	203 / 203	1705 / 1703	0 / 0	1703 / 1702	1702 / 1702	1707 / 1707	1709 / 1708
BTP 217	1481 / 1481	110 / 110	1481 / 1480	1480 / 1480	1481 / 1481	1481 / 1480	1480 / 1480	1479 / 1480	1481 / 1480	228 / 228	1480 / 1480	284 / 284	93 / 93	1480 / 1479	1481 / 1481	114 / 114	105 / 105	1702 / 1702	1463 / 1462	1703 / 1072	0 / 0	3 / 3	108 / 108	108 / 108
BTP 218	1479 / 1481	113 / 113	1479 / 1481	1478 / 1480	1479 / 1481	1479 / 1480	1478 / 1480	1477 / 1480	1479 / 1480	231 / 231	1478 / 1480	286 / 286	95 / 95	1478 / 1479	1479 / 1481	117 / 117	108 / 108	1701 / 1702	1462 / 1462	1702 / 1702	3 / 3	0 / 0	111 / 111	111 / 111
BTP 219	1488 / 1489	4 / 4	1488 / 1489	1487 / 1488	1488 / 1489	1488 / 1488	1487 / 1489	1486 / 1488	1488 / 1488	233 / 233	1487 / 1488	283 / 283	104 / 104	1487 / 1487	1488 / 1489	10 / 10	63 / 63	1706 / 1707	1466 / 1466	1707 / 1707	108 / 108	111 / 111	0 / 0	2 / 2
BTP 220	1490 / 1490	4 / 4	1490 / 1490	1489 / 1489	1490 / 1490	1490 / 1489	1489 / 1490	1488 / 1489	1490 / 1489	232 / 232	1489 / 1489	281 / 281	102 / 102	1489 / 1488	1490 / 1490	8 / 8	61 / 61	1708 / 1708	1468 / 1467	1709 / 1708	108 / 108	111 / 111	2 / 2	0 / 0
BTP 221	1487 / 1489	4 / 4	1487 / 1489	1486 / 1489	1487 / 1489	1487 / 1488	1486 / 1489	1485 / 1488	1487 / 1488	232 / 232	1486 / 1488	281 / 281	102 / 102	1486 / 1487	1487 / 1489	8 / 8	61 / 61	1706 / 1707	1465 / 1466	1707 / 1707	107 / 107	110 / 110	2 / 2	0 / 0
BTP 222	1490 / 1490	5 / 5	1490 / 1490	1489 / 1489	1490 / 1490	1490 / 1489	1489 / 1490	1488 / 1489	1490 / 1489	231 / 231	1489 / 1489	280 / 280	101 / 101	1489 / 1488	1490 / 1490	9 / 9	60 / 60	1708 / 1708	1468 / 1467	1709 / 1708	107 / 107	110 / 110	3 / 3	1 / 1
BTP 223	1486 / 1489	6 / 6	1486 / 1489	1486 / 1488	1486 / 1489	1486 / 1488	1485 / 1489	1484 / 1488	1486 / 1488	234 / 234	1485 / 1488	282 / 282	104 / 104	1485 / 1487	1486 / 1489	9 / 9	63 / 63	1704 / 1707	1465 / 1466	1705 / 1707	109 / 109	112 / 112	4 / 4	2 / 2
BTP 224	1348 / 1348	1455 / 1456	1348 / 1348	1347 / 1347	1348 / 1348	1348 / 1347	1347 / 1348	1346 / 1347	1348 / 1347	1454 / 1456	1347 / 1347	1454 / 1454	1451 / 1452	1347 / 1346	1348 / 1348	1456 / 1456	1452 / 1452	1709 / 1709	1396 / 1395	1710 / 1709	1451 / 1451	1449 / 1451	1455 / 1456	1455 / 1456
BTP 225	1488 / 1489	3 / 3	1488 / 1489	1488 / 1488	1488 / 1489	1488 / 1488	1487 / 1489	1486 / 1488	1488 / 1488	233 / 233	1487 / 1488	282 / 282	104 / 104	1487 / 1487	1488 / 1489	11 / 11	63 / 63	1705 / 1707	1465 / 1466	1706 / 1707	109 / 109	112 / 112	3 / 3	3 / 3
BTP 226	3 / 3	1487 / 1488	4 / 4	4 / 4	3 / 3	3 / 3	4 / 4	3 / 3	4 / 4	1484 / 1485	4 / 4	1486 / 1486	1485 / 1486	5 / 4	4 / 4	1486 / 1487	1487 / 1487	1708 / 1708	1369 / 1369	1710 / 1709	1480 / 1480	1478 / 1480	1487 / 1488	1489 / 1489
BTP 227	3 / 3	1488 / 1489	4 / 4	4 / 4	3 / 3	3 / 3	4 / 4	3 / 3	4 / 4	1485 / 1486	4 / 4	1487 / 1487	1486 / 1487	5 / 4	4 / 4	1487 / 1488	1488 / 1488	1709 / 1709	1370 / 1370	1711 / 1710	1481 / 1481	1479 / 1481	1488 / 1489	1490 / 1490
BTP 228	1488 / 1489	2 / 4	1488 / 1489	1487 / 1488	1488 / 1489	1488 / 1488	1487 / 1489	1486 / 1488	1488 / 1488	233 / 235	1487 / 1488	282 / 284	103 / 105	1487 / 1487	1488 / 1489	10 / 11	62 / 64	1706 / 1707	1467 / 1466	1707 / 1707	108 / 110	111 / 113	2 / 4	2 / 4
BTP 229	1491 / 1491	10 / 10	1491 / 1491	1490 / 1490	1491 / 1491	1491 / 1490	1490 / 1491	1489 / 1490	1491 / 1490	236 / 236	1490 / 1490	286 / 286	107 / 107	1490 / 1489	1491 / 1491	12 / 12	66 / 66	1707 / 1707	1468 / 1467	1708 / 1707	112 / 112	115 / 115	8 / 8	6 / 6
BTP 230	1489 / 1489	3 / 3	1489 / 1489	1488 / 1488	1489 / 1489	1489 / 1488	1488 / 1489	1487 / 1488	1489 / 1488	234 / 234	1488 / 1488	283 / 283	104 / 104	1488 / 1487	1489 / 1489	11 / 11	63 / 63	1707 / 1707	1467 / 1466	1708 / 1707	109 / 109	112 / 112	3 / 3	3 / 3
BTP 231	2 / 2	1488 / 1488	3 / 3	3 / 3	2 / 2	2 / 2	3 / 3	2 / 2	3 / 3	1485 / 1485	3 / 3	1487 / 1486	1486 / 1486	4 / 3	3 / 3	1487 / 1487	1488 / 1487	1709 / 1708	1370 / 1370	1711 / 1709	1481 / 1480	1479 / 1480	1488 / 1488	1490 / 1489
BTP 232	10 / 10	1489 / 1490	11 / 11	11 / 11	10 / 10	10 / 10	11 / 11	10 / 10	11 / 11	1486 / 1487	11 / 11	1488 / 1488	1487 / 1488	12 / 11	11 / 11	1488 / 1489	1489 / 1489	1709 / 1709	1370 / 1370	1711 / 1710	1482 / 1482	1480 / 1481	1489 / 1490	1491 / 1491
BTP 233	1489 / 1489	6 / 6	1489 / 1489	1488 / 1488	1489 / 1489	1489 / 1488	1488 / 1489	1487 / 1488	1489 / 1488	235 / 235	1488 / 1488	284 / 284	106 / 106	1488 / 1487	1489 / 1489	12 / 12	65 / 65	1707 / 1707	1469 / 1468	1708 / 1707	111 / 111	114 / 114	4 / 4	4 / 4
BTP 234	9 / 11	1487 / 1490	10 / 12	10 / 12	9 / 11	9 / 11	10 / 12	9 / 11	10 / 12	1484 / 1487	10 / 12	1486 / 1488	1485 / 1488	11 / 12	10 / 12	1486 / 1489	1487 / 1489	1707 / 1709	1368 / 1370	1709 / 1710	1480 / 1482	1478 / 1482	1487 / 1490	1489 / 1491
BTP 235	12 / 11	1485 / 1489	13 / 12	13 / 12	12 / 11	12 / 11	12 / 12	12 / 11	13 / 12	1482 / 1482	13 / 12	1484 / 1483	1483 / 1483	14 / 12	13 / 12	1484 / 1484	1485 / 1484	1705 / 1704	1366 / 1365	1707 / 1705	1478 / 1477	1476 / 1477	1485 / 1485	1487 / 1486
BTP 236	14 / 14	1489 / 1490	15 / 15	15 / 15	14 / 14	14 / 14	15 / 15	14 / 14	15 / 15	1485 / 1486	15 / 15	1487 / 1487	1486 / 1487	16 / 15	15 / 15	1488 / 1489	1488 / 1488	1708 / 1708	1370 / 1370	1710 / 1709	1481 / 1481	1479 / 1481	1489 / 1490	1490 / 1490
BTP 237	1489 / 1489	5 / 5	1489 / 1489	1488 / 1488	1489 / 1489	1489 / 1488	1488 / 1489	1487 / 1488	1489 / 1488	234 / 234	1488 / 1488	284 / 284	105 / 105	1488 / 1487	1489 / 1489	11 / 11	64 / 64	1707 / 1707	1468 / 1467	1708 / 1707	110 / 110	113 / 113	3 / 3	3 / 3
BTP 238	1489 / 1489	5 / 5	1489 / 1489	1488 / 1488	1489 / 1489	1489 / 1488	1488 / 1489	1487 / 1488	1489 / 1488	234 / 234	1488 / 1488	284 / 284	105 / 105	1488 / 1487	1489 / 1489	11 / 11	64 / 64	1707 / 1707	1468 / 1467	1708 / 1707	110 / 110	113 / 113	3 / 3	3 / 3
BTP 239	1489 / 1489	11 / 11	1489 / 1489	1488 / 1488	1489 / 1489	1489 / 1488	1488 / 1489	1487 / 1488	1489 / 1488	234 / 234	1488 / 1488	284 / 284	105 / 105	1488 / 1487	1489 / 1489	15 / 15	64 / 64	1706 / 1707	1464 / 1464	1706 / 1706	111 / 111	114 / 114	9 / 9	7 / 7
BTP 240	1489 / 1489	2 / 2	1489 / 1489	1488 / 1488	1489 / 1489	1489 / 1488	1488 / 1489	1487 / 1488	1489 / 1488	233 / 233	1488 / 1488	282 / 282	103 / 103	1488 / 1487	1489 / 1489	10 / 10	62 / 62	1706 / 1706	1467 / 1466	1707 / 1706	108 / 108	111 / 111	2 / 2	2 / 2
BTP 241	1489 / 1489	3 / 3	1489 / 1489	1488 / 1488	1489 / 1489	1489 / 1488	1488 / 1489	1487 / 1488	1489 / 1488	234 / 234	1488 / 1488	282 / 282	104 / 104	1488 / 1487	1489 / 1489	11 / 11	63 / 63	1707 / 1707	1467 / 1466	1708 / 1707	109 / 109	112 / 112	3 / 3	3 / 3
BTP 242	1489 / 1489	10 / 10	1489 / 1489	1488 / 1488	1489 / 1489	1489 / 1488	1488 / 1489	1487 / 1488	1489 / 1488	237 / 235	1488 / 1488	286 / 284	107 / 105	1488 / 1487	1489 / 1489	4 / 4	67 / 65	1707 / 1707	1467 / 1466	1708 / 1707	113 / 111	116 / 114	8 / 6	6 / 4
BTP 243	2 / 2	1488 / 1489	3 / 3	1 / 1	2 / 2	2 / 2	3 / 3	2 / 2	1 / 1	1485 / 1486	3 / 3	1487 / 1487	1486 / 1487	1 / 0	3 / 3	1487 / 1488	1488 / 1488	1708 / 1708	1370 / 1370	1710 / 1709	1481 / 1481	1479 / 1481	1488 / 1489	1490 / 1490
BTP 244	1488 / 1488	11 / 11	1488 / 1488	1487 / 1487	1488 / 1488	1488 / 1487	1487 / 1488	1486 / 1487	1488 / 1487	238 / 238	1487 / 1487	286 / 286	108 / 108	1487 / 1486	1488 / 1488	1 / 1	68 / 68	1706 / 1706	1467 / 1466	1707 / 1706	113 / 113	116 / 116	9 / 9	7 / 7
BTP 245	2 / 2	1488 / 1489	3 / 3	1 / 1	2 / 2	2 / 2	3 / 3	2 / 2	1 / 1	1485 / 1486	3 / 3	1487 / 1487	1486 / 1487	1 / 0	3 / 3	1487 / 1488	1488 / 1488	1709 / 1709	1370 / 1370	1711 / 1710	1481 / 1481	1479 / 1481	1488 / 1489	1490 / 1490
BTP 247	1488 / 1489	10 / 10	1488 / 1489	1487 / 1488	1488 / 1489	1488 / 1488	1487 / 1489	1486 / 1488	1488 / 1488	238 / 238	1487 / 1488	286 / 286	107 / 107	1487 / 1487	1488 / 1489	10 / 10	67 / 67	1704 / 1706	1466 / 1466	1705 / 1706	113 / 113	116 / 116	8 / 8	6 / 6
BTP 248	1 / 1	1487 / 1489	2 / 2	2 / 2	1 / 1	1 / 1	2 / 2	1 / 1	2 / 2	1485 / 1486	2 / 2	1486 / 1487	1486 / 1487	3 / 2	0 / 0	1486 / 1488	1487 / 1488	1708 / 1709	1369 / 1370	1710 / 1710	1480 / 1481	1478 / 1481	1487 / 1489	1489 / 1490
BTP 249	5 / 5	1488 / 1489	6 / 6	6 / 6	5 / 5	5 / 5	6 / 6	5 / 5	6 / 6	1485 / 1486	6 / 6	1487 / 1487	1486 / 1487	7 / 6	6 / 6	1487 / 1488	1488 / 1488	1709 / 1709	1371 / 1371	1711 / 1710	1481 / 1481	1479 / 1481	1488 / 1489	1490 / 1490
BTP 250	1691 / 1691	1690 / 1691	1691 / 1691	1690 / 1690	1691 / 1691	1691 / 1690	1690 / 1691	1689 / 1690	1691 / 1690	1691 / 1693	1690 / 1690	1693 / 1693	1688 / 1689	1689 / 1688	1691 / 1691	1688 / 1690	1690 / 1690	1661 / 1661	1683 / 1682	1663 / 1662	1687 / 1687	1686 / 1687	1690 / 1691	1692 / 1692
BTP 251	1348 / 1347	1454 / 1454	1348 / 1347	1348 / 1346	1348 / 1347	1348 / 1346	1347 / 1347	1346 / 1346	1348 / 1346	1454 / 1454	1347 / 1346	1453 / 1452	1451 / 1450	1347 / 1345	1348 / 1347	1455 / 1454	1451 / 1450	1708 / 1707	1396 / 1394	1709 / 1707	1450 / 1449	1448 / 1449	1454 / 1454	1455 / 1454
BTP 252	1699 / 1699	1701 / 1702	1699 / 1699	1698 / 1698	1699 / 1699	1699 / 1698	1698 / 1699	1696 / 1698	1699 / 1698	1701 / 1702	1698 / 1698	1705 / 1705	1697 / 1700	1698 / 1697	1699 / 1699	1699 / 1702	1701 / 1701	1686 / 1686	1689 / 1688	1685 / 1684	1697 / 1679	1695 / 1697	1701 / 1702	1703 / 1703
BTP 253	8 / 8	1488 / 1490	9 / 9	9 / 9	8 / 8	8 / 8	9 / 9	8 / 8	9 / 9	1486 / 1487	9 / 9	1487 / 1488	1487 / 1488	10 / 9	9 / 9	1487 / 1489	1488 / 1489	1708 / 1709	1371 / 1372	1710 / 1710	1481 / 1482	1479 / 1482	1488 / 1490	1490 / 1491
BTP 254	1489 / 1489	9 / 9	1489 / 1489	1488 / 1488	1489 / 1489	1489 / 1488	1488 / 1489	1487 / 1488	1489 / 1488	235 / 235	1488 / 1488	283 / 283	107 / 107	1488 / 1487	1489 / 1489	9 / 9	66 / 66	1706 / 1706	1466 / 1465	1708 / 1707	112 / 112	115 / 115	7 / 7	5 / 5
BTP 255	1392 / 1392	1467 / 1468	1392 / 1392	1391 / 1391	1392 / 1392	1392 / 1391	1391 / 1392	1389 / 1391	1392 / 1391	1463 / 1464	1391 / 1391	1464 / 1464	1462 / 1464	1390 / 1389	1392 / 1392	1465 / 1467	1467 / 1467	1703 / 1703	1409 / 1408	1704 / 1703	1463 / 1463	1462 / 1463	1467 / 1468	1469 / 1469
BTP 256	1489 / 1489	10 / 10	1489 / 1489	1488 / 1488	1489 / 1489	1489 / 1488	1488 / 1489	1487 / 1488	1489 / 1488	238 / 238	1488 / 1488	286 / 286	109 / 109	1488 / 1487	1489 / 1489	16 / 16	68 / 68	1706 / 1706	1470 / 1469	1707 / 1706	114 / 114	117 / 117	8 / 8	8 / 8
BTP 257	10 / 10	1488 / 1489	11 / 11	11 / 11	10 / 10	10 / 10	11 / 11	10 / 10	11 / 11	1485 / 1486	11 / 11	1487 / 1487	1486 / 1487	12 / 11	11 / 11	1487 / 1488	1488 / 1488	1708 / 1708	1371 / 1371	1710 / 1709	1481 / 1481	1479 / 1481	1488 / 1489	1490 / 1490
BTP 258	1489 / 1489	10 / 10	1489 / 1489	1488 / 1488	1489 / 1489	1489 / 1488	1488 / 1489	1487 / 1488	1489 / 1488	238 / 238	1488 / 1488	286 / 286	109 / 109	1488 / 1487	1489 / 1489	16 / 16	68 / 68	1706 / 1706	1470 / 1469	1707 / 1706	114 / 114	117 / 117	8 / 8	8 / 8
USA100	1487 / 1488	79 / 79	1487 / 1488	1486 / 1487	1487 / 1488	1487 / 1487	1486 / 1488	1485 / 1487	1487 / 1487	208 / 208	1486 / 1487	258 / 258	59 / 59	1486 / 1486	1487 / 1488	84 / 84	74 / 74	1708 / 1709	1464 / 1464	1709 / 1709	67 / 67	70 / 70	78 / 78	76 / 76
USA300	80 / 80	1487 / 1488	81 / 81	81 / 81	80 / 80	80 / 80	81 / 81	80 / 80	81 / 81	1484 / 1485	81 / 81	1486 / 1486	1485 / 1486	82 / 81	81 / 81	1486 / 1487	1487 / 1487	1713 / 1713	1370 / 1370	1715 / 1714	1480 / 1480	1478 / 1480	1487 / 1488	1489 / 1489
USA400	1385 / 1385	1466 / 1467	1385 / 1385	1384 / 1384	1385 / 1385	1385 / 1384	1384 / 1385	1383 / 1384	1385 / 1384	1461 / 1642	1384 / 1384	1460 / 1460	1463 / 1465	1383 / 1382	1385 / 1385	1465 / 1467	1466 / 1466	1703 / 1703	1400 / 1399	1704 / 1703	1462 / 1462	1461 / 1462	1466 / 1467	1468 / 1468
USA500	247 / 247	1494 / 1496	248 / 248	248 / 248	247 / 247	247 / 247	248 / 248	247 / 248	248 / 248	1496 / 1497	248 / 248	1495 / 1496	1493 / 1494	249 / 248	248 / 248	1493 / 1495	1494 / 1495	1711 / 1712	1372 / 1373	1712 / 1712	1487 / 1488	1485 / 1488	1494 / 1496	1496 / 1497
USA600	1713 / 1713	1709 / 1710	1713 / 1713	1712 / 1712	1713 / 1713	1713 / 1712	1712 / 1713	1710 / 1712	1713 / 1712	1705 / 1707	1713 / 1713	1709 / 1709	1704 / 1706	1711 / 1710	1713 / 1713	1707 / 1709	1709 / 1709	199 / 199	1708 / 1707	233 / 233	1705 / 1705	1704 / 1705	1709 / 1710	1711 / 1711
USA700	1420 / 1419	1461 / 1461	1420 / 1419	1419 / 1418	1420 / 1419	1420 / 1418	1419 / 1419	1418 / 1418	1420 / 1418	1455 / 1455	1419 / 1418	1459 / 1458	1462 / 1462	1418 / 1416	1420 / 1419	1461 / 1460	1461 / 1460	1698 / 1697	1418 / 1416	1699 / 1697	1456 / 1455	1455 / 1455	1461 / 1461	1463 / 1462
USA800	1481 / 1481	200 / 200	1481 / 1481	1480 / 1480	1481 / 1481	1481 / 1480	1480 / 1481	1479 / 1480	1481 / 1480	161 / 161	1480 / 1480	222 / 222	198 / 198	1480 / 1479	1481 / 1481	203 / 203	192 / 192	1707 / 1701	1466 / 1465	1708 / 1707	198 / 198	201 / 201	199 / 199	197 / 197
USA900	1341 / 1341	1446 / 1447	1341 / 1341	1340 / 1340	1341 / 1341	1341 / 1340	1340 / 1341	1339 / 1340	1341 / 1340	1443 / 1445	1340 / 1340	1444 / 1444	1441 / 1442	1340 / 1339	1341 / 1341	1446 / 1447	1444 / 1444	1710 / 1710	1389 / 1388	1711 / 1710	1441 / 1441	1439 / 1441	1446 / 1447	1447 / 1447
USA1000	1701 / 1701	1704 / 1705	1701 / 1701	1700 / 1700	1701 / 1701	1701 / 1700	1700 / 1701	1698 / 1700	1701 / 1700	1704 / 1705	1700 / 1700	1708 / 1708	1700 / 1703	1700 / 1699	1701 / 1701	1702 / 1705	1704 / 1704	1690 / 1690	1691 / 1690	1689 / 1688	1700 / 1700	1698 / 1700	1704 / 1705	1706 / 1706

Isolate	BTP 221	BTP 222	BTP 223	BTP 224	BTP 225	BTP 226	BTP 227	BTP 228	BTP 229	BTP 230	BTP 231	BTP 232	BTP 233	BTP 234	BTP 235	BTP 236	BTP 237	BTP 238	BTP 239	BTP 240	BTP 241
BTP 197	1487 / 1489	1490 / 1490	1486 / 1489	1348 / 1348	1488 / 1489	3 / 3	3 / 3	1488 / 1489	1491 / 1491	1489 / 1489	2 / 2	10 / 10	1489 / 1489	9 / 11	12 / 11	14 / 14	1489 / 1489	1489 / 1489	1489 / 1489	1489 / 1489	1489 / 1489
BTP 198	4 / 4	5 / 5	6 / 6	1455 / 1456	3 / 3	1487 / 1488	1488 / 1489	2 / 4	10 / 10	3 / 3	1488 / 1488	1489 / 1490	6 / 6	1487 / 1490	1485 / 1489	1489 / 1490	5 / 5	5 / 5	11 / 11	2 / 2	3 / 3
BTP 199	1490 / 1490	1490 / 1490	1486 / 1489	1348 / 1348	1488 / 1489	4 / 4	4 / 4	1488 / 1489	1491 / 1491	1489 / 1489	3 / 3	11 / 11	1489 / 1489	10 / 12	13 / 12	15 / 15	1489 / 1489	1489 / 1489	1489 / 1489	1489 / 1489	1489 / 1489
BTP 200	1486 / 1489	1489 / 1489	1486 / 1488	1347 / 1347	1488 / 1488	4 / 4	4 / 4	1487 / 1488	1490 / 1490	1488 / 1488	3 / 3	11 / 11	1488 / 1488	10 / 12	13 / 12	15 / 15	1488 / 1488	1488 / 1488	1488 / 1488	1488 / 1488	1488 / 1488
BTP 201	1487 / 1489	1490 / 1490	1486 / 1489	1348 / 1348	1488 / 1489	3 / 3	3 / 3	1488 / 1489	1491 / 1491	1489 / 1489	2 / 2	10 / 10	1489 / 1489	9 / 11	12 / 11	14 / 14	1489 / 1489	1489 / 1489	1489 / 1489	1489 / 1489	1489 / 1489
BTP 202	1487 / 1488	1490 / 1489	1486 / 1488	1348 / 1347	1488 / 1488	3 / 3	3 / 3	1488 / 1488	1491 / 1490	1489 / 1488	2 / 2	10 / 10	1489 / 1488	9 / 11	12 / 11	14 / 14	1489 / 1488	1489 / 1488	1489 / 1488	1489 / 1488	1489 / 1488
BTP 203	1486 / 1489	1489 / 1490	1485 / 1489	1347 / 1348	1487 / 1489	4 / 4	4 / 4	1487 / 1489	1490 / 1491	1488 / 1489	3 / 3	11 / 11	1488 / 1489	10 / 12	12 / 12	15 / 15	1488 / 1489	1488 / 1489	1488 / 1489	1488 / 1489	1488 / 1489
BTP 204	1485 / 1488	1488 / 1489	1484 / 1488	1346 / 1347	1486 / 1488	3 / 3	3 / 3	1486 / 1488	1489 / 1490	1487 / 1488	2 / 2	10 / 10	1487 / 1488	9 / 11	12 / 11	14 / 14	1487 / 1488	1487 / 1488	1487 / 1488	1487 / 1488	1487 / 1488
BTP 205	1487 / 1488	1490 / 1489	1486 / 1488	1348 / 1347	1488 / 1488	4 / 4	4 / 4	1488 / 1488	1491 / 1490	1489 / 1488	3 / 3	11 / 11	1489 / 1488	10 / 12	13 / 12	15 / 15	1489 / 1488	1489 / 1488	1489 / 1488	1489 / 1488	1489 / 1488
BTP 206	232 / 232	231 / 231	234 / 234	1454 / 1456	233 / 233	1484 / 1485	1485 / 1486	233 / 235	236 / 236	234 / 234	1485 / 1485	1486 / 1487	235 / 235	1484 / 1487	1482 / 1482	1485 / 1486	234 / 234	234 / 234	234 / 234	233 / 233	234 / 234
BTP 207	1486 / 1488	1489 / 1489	1485 / 1488	1347 / 1347	1487 / 1488	4 / 4	4 / 4	1487 / 1488	1490 / 1490	1488 / 1488	3 / 3	11 / 11	1488 / 1488	10 / 12	13 / 12	15 / 15	1488 / 1488	1488 / 1488	1488 / 1488	1488 / 1488	1488 / 1488
BTP 208	281 / 281	280 / 280	282 / 282	1454 / 1454	282 / 282	1486 / 1486	1487 / 1487	282 / 284	286 / 286	283 / 283	1487 / 1486	1488 / 1488	284 / 284	1486 / 1488	1484 / 1483	1487 / 1487	284 / 284	284 / 284	284 / 284	282 / 282	282 / 282
BTP 209	102 / 102	101 / 101	104 / 104	1451 / 1452	104 / 104	1485 / 1486	1486 / 1487	103 / 105	107 / 107	104 / 104	1486 / 1486	1487 / 1488	106 / 106	1485 / 1488	1483 / 1483	1486 / 1487	105 / 105	105 / 105	105 / 105	103 / 103	104 / 104
BTP 210	1486 / 1487	1489 / 1488	1485 / 1487	1347 / 1346	1487 / 1487	5 / 4	5 / 4	1487 / 1487	1490 / 1489	1488 / 1487	4 / 3	12 / 11	1488 / 1487	11 / 12	14 / 12	16 / 15	1488 / 1487	1488 / 1487	1488 / 1487	1488 / 1487	1488 / 1487
BTP 211	1487 / 1489	1490 / 1490	1486 / 1489	1348 / 1348	1488 / 1489	4 / 4	4 / 4	1488 / 1489	1491 / 1491	1489 / 1489	3 / 3	11 / 11	1489 / 1489	10 / 12	13 / 12	15 / 15	1489 / 1489	1489 / 1489	1489 / 1489	1489 / 1489	1489 / 1489
BTP 212	8 / 8	9 / 9	9 / 9	1456 / 1456	11 / 11	1486 / 1487	1487 / 1488	10 / 11	12 / 12	11 / 11	1487 / 1487	1488 / 1489	12 / 12	1486 / 1489	1484 / 1484	1488 / 1489	11 / 11	11 / 11	15 / 15	10 / 10	11 / 11
BTP 213	61 / 61	60 / 60	63 / 63	1452 / 1452	63 / 63	1487 / 1487	1488 / 1488	62 / 64	66 / 66	63 / 63	1488 / 1487	1489 / 1489	65 / 65	1487 / 1489	1485 / 1484	1488 / 1488	64 / 64	64 / 64	64 / 64	62 / 62	63 / 63
BTP 214	1706 / 1707	1708 / 1708	1704 / 1707	1709 / 1709	1705 / 1707	1708 / 1708	1709 / 1709	1706 / 1707	1707 / 1707	1707 / 1707	1709 / 1708	1709 / 1709	1707 / 1707	1707 / 1709	1705 / 1704	1708 / 1708	1707 / 1707	1707 / 1707	1706 / 1707	1706 / 1706	1707 / 1707
BTP 215	1465 / 1466	1468 / 1467	1465 / 1466	1396 / 1395	1465 / 1466	1369 / 1369	1370 / 1370	1467 / 1466	1468 / 1467	1467 / 1466	1370 / 1370	1370 / 1370	1469 / 1468	1368 / 1370	1366 / 1365	1370 / 1370	1468 / 1467	1468 / 1467	1464 / 1464	1467 / 1466	1467 / 1466
BTP 216	1707 / 1707	1709 / 1708	1705 / 1707	1710 / 1709	1706 / 1707	1710 / 1709	1711 / 1710	1707 / 1707	1708 / 1707	1708 / 1707	1711 / 1709	1711 / 1710	1708 / 1707	1709 / 1710	1707 / 1705	1710 / 1709	1708 / 1707	1708 / 1707	1706 / 1706	1707 / 1706	1708 / 1707
BTP 217	107 / 107	107 / 107	109 / 109	1451 / 1451	109 / 109	1480 / 1480	1481 / 1481	108 / 110	112 / 112	109 / 109	1481 / 1480	1482 / 1482	111 / 111	1480 / 1482	1478 / 1477	1481 / 1481	110 / 110	110 / 110	111 / 111	108 / 108	109 / 109
BTP 218	110 / 110	110 / 110	112 / 112	1449 / 1451	112 / 112	1478 / 1480	1479 / 1481	111 / 113	115 / 115	112 / 112	1479 / 1480	1480 / 1481	114 / 114	1478 / 1482	1476 / 1477	1479 / 1481	113 / 113	113 / 113	114 / 114	111 / 111	112 / 112
BTP 219	2 / 2	3 / 3	4 / 4	1455 / 1456	3 / 3	1487 / 1488	1488 / 1489	2 / 4	8 / 8	3 / 3	1488 / 1488	1489 / 1490	4 / 4	1487 / 1490	1485 / 1485	1489 / 1490	3 / 3	3 / 3	9 / 9	2 / 2	3 / 3
BTP 220	0 / 0	1 / 1	2 / 2	1456 / 1456	3 / 3	1489 / 1489	1490 / 1490	2 / 4	6 / 6	3 / 3	1490 / 1489	1491 / 1491	4 / 4	1489 / 1491	1487 / 1486	1490 / 1490	3 / 3	3 / 3	7 / 7	2 / 2	3 / 3
BTP 221	0 / 0	1 / 1	2 / 2	1456 / 1456	3 / 3	1486 / 1488	1487 / 1489	2 / 4	6 / 6	3 / 3	1487 / 1488	1488 / 1490	4 / 4	1486 / 1490	1484 / 1485	1488 / 1490	3 / 3	3 / 3	7 / 7	2 / 2	3 / 3
BTP 222	1 / 1	0 / 0	3 / 3	1456 / 1456	4 / 4	1489 / 1489	1490 / 1490	3 / 5	7 / 7	4 / 4	1490 / 1489	1491 / 1491	5 / 5	1489 / 1491	1487 / 1486	1490 / 1490	4 / 4	4 / 4	6 / 6	3 / 3	4 / 4
BTP 223	2 / 2	3 / 3	0 / 0	1454 / 1456	5 / 5	1485 / 1488	1486 / 1489	4 / 6	8 / 8	5 / 5	1486 / 1488	1487 / 1490	6 / 6	1485 / 1490	1483 / 1485	1487 / 1490	5 / 5	5 / 5	9 / 9	4 / 4	5 / 5
BTP 224	1456 / 1456	1456 / 1456	1454 / 1456	0 / 0	1454 / 1456	1347 / 1347	1348 / 1348	1455 / 1456	1457 / 1457	1456 / 1456	1348 / 1347	1346 / 1346	1456 / 1456	1344 / 1346	1344 / 1343	1345 / 1345	1457 / 1457	1457 / 1457	1454 / 1455	1456 / 1456	1456 / 1456
BTP 225	3 / 3	4 / 4	5 / 5	1454 / 1456	0 / 0	1487 / 1488	1488 / 1489	1 / 3	9 / 9	2 / 2	1488 / 1488	1489 / 1490	5 / 5	1487 / 1490	1485 / 1485	1489 / 1490	4 / 4	4 / 4	10 / 10	1 / 1	2 / 2
BTP 226	1486 / 1488	1489 / 1489	1485 / 1488	1347 / 1347	1487 / 1488	0 / 0	0 / 0	1487 / 1488	1490 / 1490	1488 / 1488	1 / 1	7 / 7	1488 / 1488	7 / 9	9 / 8	11 / 11	1488 / 1488	1488 / 1488	1488 / 1488	1488 / 1488	1488 / 1488
BTP 227	1487 / 1489	1490 / 1490	1486 / 1489	1348 / 1348	1488 / 1489	0 / 0	0 / 0	1488 / 1489	1491 / 1491	1489 / 1489	1 / 1	7 / 7	1489 / 1489	7 / 9	9 / 8	11 / 11	1489 / 1489	1489 / 1489	1489 / 1489	1489 / 1489	1489 / 1489
BTP 228	2 / 4	3 / 5	4 / 6	1455 / 1456	1 / 3	1487 / 1488	1488 / 1489	0 / 0	8 / 10	1 / 3	1488 / 1488	1489 / 1490	4 / 6	1487 / 1485	1485 / 1485	1489 / 1490	3 / 5	3 / 5	9 / 11	0 / 2	1 / 3
BTP 229	6 / 6	7 / 7	8 / 8	1457 / 1457	9 / 9	1490 / 1490	1491 / 1491	8 / 10	0 / 0	9 / 9	1491 / 1490	1492 / 1492	10 / 10	1490 / 1492	1488 / 1487	1492 / 1492	9 / 9	9 / 9	13 / 13	8 / 8	9 / 9
BTP 230	3 / 3	4 / 4	5 / 5	1456 / 1456	2 / 2	1488 / 1488	1489 / 1489	1 / 3	9 / 9	0 / 0	1489 / 1488	1490 / 1490	5 / 5	1488 / 1490	1486 / 1485	1490 / 1490	4 / 4	4 / 4	10 / 10	1 / 1	2 / 2
BTP 231	1487 / 1488	1490 / 1489	1486 / 1488	1348 / 1347	1488 / 1488	1 / 1	1 / 1	1488 / 1488	1491 / 1490	1489 / 1488	0 / 0	8 / 8	1489 / 1488	8 / 10	10 / 8	12 / 12	1489 / 1488	1489 / 1488	1489 / 1488	1489 / 1488	1489 / 1488
BTP 232	1488 / 1490	1491 / 1491	1487 / 1490	1346 / 1346	1489 / 1490	7 / 7	7 / 7	1489 / 1490	1492 / 1492	1490 / 1490	8 / 8	0 / 0	1490 / 1490	0 / 2	2 / 1	4 / 4	1490 / 1490	1490 / 1490	1490 / 1490	1490 / 1490	1490 / 1490
BTP 233	4 / 4	5 / 5	6 / 6	1456 / 1456	5 / 5	1488 / 1488	1489 / 1489	4 / 6	10 / 10	5 / 5	1489 / 1488	1490 / 1490	0 / 0	1488 / 1490	1486 / 1485	1490 / 1490	3 / 3	3 / 3	11 / 11	4 / 4	5 / 5
BTP 234	1486 / 1490	1489 / 1491	1485 / 1490	1344 / 1346	1487 / 1490	7 / 9	7 / 9	1487 / 1485	1490 / 1492	1488 / 1490	8 / 10	0 / 2	1488 / 1490	0 / 0	2 / 3	4 / 6	1488 / 1490	1488 / 1490	1488 / 1490	1488 / 1490	1488 / 1490
BTP 235	1484 / 1485	1487 / 1486	1483 / 1485	1344 / 1343	1485 / 1485	9 / 8	9 / 8	1485 / 1485	1488 / 1487	1486 / 1485	10 / 9	1 / 2	1486 / 1485	2 / 3	0 / 0	6 / 5	1486 / 1485	1486 / 1485	1486 / 1485	1486 / 1485	1486 / 1485
BTP 236	1488 / 1490	1490 / 1490	1487 / 1490	1345 / 1345	1489 / 1490	11 / 11	11 / 11	1489 / 1490	1492 / 1492	1490 / 1490	12 / 12	4 / 4	1490 / 1490	4 / 6	6 / 5	0 / 0	1490 / 1490	1490 / 1490	1490 / 1490	1490 / 1490	1490 / 1490
BTP 237	3 / 3	4 / 4	5 / 5	1457 / 1457	4 / 4	1488 / 1488	1489 / 1489	3 / 5	9 / 9	4 / 4	1489 / 1488	1490 / 1490	3 / 3	1488 / 1490	1486 / 1485	1490 / 1490	0 / 0	0 / 0	10 / 10	3 / 3	4 / 4
BTP 238	3 / 3	4 / 4	5 / 5	1457 / 1457	4 / 4	1488 / 1488	1489 / 1489	3 / 5	9 / 9	4 / 4	1489 / 1488	1490 / 1490	3 / 3	1488 / 1490	1486 / 1485	1490 / 1490	0 / 0	0 / 0	10 / 10	3 / 3	4 / 4
BTP 239	7 / 7	6 / 6	9 / 9	1454 / 1455	10 / 10	1488 / 1488	1489 / 1489	9 / 11	13 / 13	10 / 10	1489 / 1488	1490 / 1490	11 / 11	1488 / 1490	1486 / 1485	1489 / 1489	10 / 10	10 / 10	0 / 0	9 / 9	10 / 10
BTP 240	2 / 2	3 / 3	4 / 4	1456 / 1456	1 / 1	1488 / 1488	1489 / 1489	0 / 2	8 / 8	1 / 1	1489 / 1488	1490 / 1490	4 / 4	1488 / 1490	1486 / 1485	1490 / 1490	3 / 3	3 / 3	9 / 9	0 / 0	1 / 1
BTP 241	3 / 3	4 / 4	5 / 5	1456 / 1456	2 / 2	1488 / 1488	1489 / 1489	1 / 3	9 / 9	2 / 2	1489 / 1488	1490 / 1490	5 / 5	1488 / 1490	1486 / 1485	1490 / 1490	4 / 4	4 / 4	10 / 10	1 / 1	0 / 0
BTP 242	6 / 4	7 / 5	8 / 6	1457 / 1456	9 / 7	1488 / 1488	1489 / 1489	8 / 7	10 / 8	9 / 7	1489 / 1488	1490 / 1490	10 / 8	1488 / 1490	1486 / 1485	1490 / 1490	9 / 7	9 / 7	13 / 11	8 / 6	9 / 7
BTP 243	1487 / 1489	1490 / 1490	1486 / 1489	1348 / 1348	1488 / 1489	5 / 5	5 / 5	1488 / 1489	1491 / 1491	1489 / 1489	4 / 4	12 / 12	1489 / 1489	11 / 13	14 / 13	16 / 16	1489 / 1489	1489 / 1489	1489 / 1489	1489 / 1489	1489 / 1489
BTP 244	7 / 7	8 / 8	8 / 8	1456 / 1456	10 / 10	1487 / 1487	1488 / 1488	9 / 10	11 / 11	10 / 10	1488 / 1487	1489 / 1489	11 / 11	1487 / 1489	1485 / 1484	1489 / 1489	10 / 10	10 / 10	14 / 14	9 / 9	10 / 10
BTP 245	1487 / 1489	1490 / 1490	1486 / 1489	1348 / 1348	1488 / 1489	5 / 5	5 / 5	1488 / 1489	1491 / 1491	1489 / 1489	4 / 4	12 / 12	1489 / 1489	11 / 13	14 / 13	16 / 16	1489 / 1489	1489 / 1489	1489 / 1489	1489 / 1489	1489 / 1489
BTP 247	6 / 6	7 / 7	8 / 8	1453 / 1455	9 / 9	1487 / 1488	1488 / 1489	8 / 9	10 / 10	9 / 9	1488 / 1488	1489 / 1490	10 / 10	1487 / 1490	1485 / 1485	1489 / 1490	9 / 9	9 / 9	13 / 13	8 / 8	9 / 9
BTP 248	1486 / 1489	1489 / 1490	1486 / 1489	1347 / 1348	1488 / 1489	4 / 4	4 / 4	1487 / 1489	1490 / 1491	1488 / 1489	3 / 3	11 / 11	1488 / 1489	10 / 12	13 / 12	15 / 15	1488 / 1489	1488 / 1489	1488 / 1489	1488 / 1489	1488 / 1489
BTP 249	1487 / 1489	1490 / 1490	1486 / 1489	1349 / 1349	1488 / 1489	8 / 8	8 / 8	1488 / 1489	1491 / 1491	1489 / 1489	7 / 7	15 / 15	1489 / 1489	14 / 16	17 / 16	19 / 19	1489 / 1489	1489 / 1489	1489 / 1489	1489 / 1489	1489 / 1489
BTP 250	1690 / 1691	1692 / 1692	1688 / 1691	1690 / 1690	1689 / 1691	1690 / 1690	1691 / 1691	1690 / 1691	1692 / 1692	1691 / 1691	1691 / 1690	1691 / 1691	1691 / 1691	1689 / 1691	1687 / 1686	1690 / 1690	1692 / 1692	1692 / 1692	1690 / 1691	1690 / 1690	1691 / 1691
BTP 251	1455 / 1454	1455 / 1454	1454 / 1454	4 / 4	1454 / 1454	1347 / 1346	1348 / 1347	1454 / 1454	1456 / 1455	1455 / 1454	1348 / 1346	1346 / 1345	1455 / 1454	1344 / 1345	1344 / 1342	1345 / 1344	1456 / 1455	1456 / 1455	1453 / 1453	1455 / 1454	1455 / 1454
BTP 252	1701 / 1702	1703 / 1703	1699 / 1702	1696 / 1696	1700 / 1702	1698 / 1698	1699 / 1699	1701 / 1702	1703 / 1703	1702 / 1702	1699 / 1698	1699 / 1699	1702 / 1702	1697 / 1699	1695 / 1694	1698 / 1698	1701 / 1702	1702 / 1702	1700 / 1701	1701 / 1701	1702 / 1702
BTP 253	1487 / 1490	1490 / 1491	1487 / 1490	1349 / 1350	1489 / 1490	11 / 11	11 / 11	1488 / 1490	1491 / 1492	1489 / 1490	10 / 10	18 / 18	1489 / 1490	17 / 19	20 / 19	22 / 22	1489 / 1490	1489 / 1490	1489 / 1490	1488 / 1489	1489 / 1490
BTP 254	5 / 5	6 / 6	7 / 7	1456 / 1456	8 / 8	1488 / 1488	1489 / 1489	7 / 8	9 / 9	8 / 8	1489 / 1488	1490 / 1490	9 / 9	1488 / 1490	1486 / 1485	1490 / 1490	8 / 8	8 / 8	12 / 12	7 / 7	8 / 8
BTP 255	1467 / 1468	1469 / 1469	1466 / 1468	1347 / 1347	1466 / 1468	1391 / 1391	1392 / 1392	1467 / 1468	1469 / 1469	1468 / 1468	1392 / 1391	1393 / 1393	1469 / 1469	1391 / 1393	1390 / 1389	1393 / 1393	1470 / 1470	1470 / 1470	1466 / 1467	1468 / 1468	1468 / 1468
BTP 256	8 / 8	9 / 9	10 / 10	1456 / 1456	9 / 9	1488 / 1488	1489 / 1489	8 / 10	14 / 14	9 / 9	1489 / 1488	1490 / 1490	8 / 8	1488 / 1490	1486 / 1485	1490 / 1490	7 / 7	7 / 7	15 / 15	8 / 8	9 / 9
BTP 257	1487 / 1489	1490 / 1490	1486 / 1489	1350 / 1350	1488 / 1489	13 / 13	13 / 13	1488 / 1489	1491 / 1491	1489 / 1489	12 / 12	20 / 20	1489 / 1489	19 / 21	22 / 21	24 / 24	1489 / 1489	1489 / 1489	1489 / 1489	1489 / 1489	1489 / 1489
BTP 258	8 / 8	9 / 9	10 / 10	1456 / 1456	9 / 9	1488 / 1488	1489 / 1489	8 / 10	14 / 14	9 / 9	1489 / 1488	1490 / 1490	8 / 8	1488 / 1490	1486 / 1485	1490 / 1490	7 / 7	7 / 7	15 / 15	8 / 8	9 / 9
USA100	76 / 76	75 / 75	78 / 78	1453 / 1454	78 / 78	1486 / 1487	1487 / 1488	77 / 79	81 / 81	78 / 78	1487 / 1487	1488 / 1489	80 / 80	1486 / 1489	1484 / 1484	1487 / 1488	79 / 79	79 / 79	79 / 79	77 / 77	78 / 78
USA300	1486 / 1488	1489 / 1489	1485 / 1488	1344 / 1344	1487 / 1488	78 / 78	78 / 78	1487 / 1488	1490 / 1490	1488 / 1488	79 / 79	77 / 77	1488 / 1488	75 / 77	78 / 77	80 / 80	1488 / 1488	1488 / 1488	1489 / 1489	1488 / 1488	1488 / 1488
USA400	1466 / 1467	1468 / 1468	1465 / 1467	1334 / 1334	1465 / 1467	1384 / 1384	1385 / 1385	1466 / 1467	1468 / 1468	1467 / 1467	1385 / 1384	1386 / 1386	1468 / 1468	1384 / 1386	1382 / 1381	1386 / 1386	1469 / 1469	1469 / 1469	1465 / 1466	1467 / 1467	1467 / 1467
USA500	1493 / 1496	1496 / 1497	1493 / 1496	1347 / 1348	1495 / 1496	246 / 246	246 / 246	1494 / 1496	1497 / 1498	1495 / 1496	247 / 247	246 / 246	1495 / 1496	245 / 247	247 / 246	250 / 250	1495 / 1496	1495 / 1496	1496 / 1497	1495 / 1496	1495 / 1496
USA600	1709 / 1710	1711 / 1711	1707 / 1710	1712 / 1712	1708 / 1710	1712 / 1712	1713 / 1713	1709 / 1710	1710 / 1710	1710 / 1710	1713 / 1712	1713 / 1713	1710 / 1710	1711 / 1713	1709 / 1708	1712 / 1712	1710 / 1710	1710 / 1710	1708 / 1709	1709 / 1709	1710 / 1710
USA700	1462 / 1461	1463 / 1462	1460 / 1461	1397 / 1396	1461 / 1461	1419 / 1418	1420 / 1419	1462 / 1461	1462 / 1461	1462 / 1461	1420 / 1418	1421 / 1420	1462 / 1461	1419 / 1420	1417 / 1415	1420 / 1419	1462 / 1461	1462 / 1461	1462 / 1461	1462 / 1461	1462 / 1461
USA800	197 / 197	196 / 196	199 / 199	1450 / 1450	198 / 198	1480 / 1480	1481 / 1481	198 / 200	202 / 202	199 / 199	1481 / 1480	1482 / 1482	200 / 200	1480 / 1482	1478 / 1477	1481 / 1481	199 / 199	199 / 199	199 / 199	198 / 198	199 / 199
USA900	1446 / 1447	1447 / 1447	1445 / 1447	117 / 117	1445 / 1447	1340 / 1340	1341 / 1341	1446 / 1447	1448 / 1448	1447 / 1447	1341 / 1340	1340 / 1340	1447 / 1447	1338 / 1340	1338 / 1337	1339 / 1339	1448 / 1448	1448 / 1448	1445 / 1446	1447 / 1447	1447 / 1447
USA1000	1704 / 1705	1706 / 1706	1702 / 1705	1698 / 1698	1703 / 1705	1700 / 1700	1701 / 1701	1704 / 1705	1706 / 1706	1705 / 1705	1701 / 1700	1701 / 1701	1705 / 1705	1699 / 1701	1697 / 1696	1700 / 1700	1704 / 1705	1705 / 1705	1703 / 1704	1704 / 1704	1705 / 1705

Isolate	BTP 242	BTP 243	BTP 244	BTP 245	BTP 247	BTP 248	BTP 249	BTP 250	BTP 251	BTP 252	BTP 253	BTP 254	BTP 255	BTP 256	BTP 257	BTP 258	USA100	USA300	USA400	USA500	USA600	USA700	USA800	USA900	USA1000
BTP 197	1489 / 1489	2 / 2	1488 / 1488	2 / 2	1488 / 1489	1 / 1	5 / 5	1691 / 1691	1348 / 1347	1699 / 1699	8 / 8	1489 / 1489	1392 / 1392	1489 / 1489	10 / 10	1489 / 1489	1487 / 1488	80 / 80	1385 / 1385	247 / 247	1713 / 1713	1420 / 1419	1481 / 1481	1341 / 1341	1701 / 1701
BTP 198	10 / 10	1488 / 1489	11 / 11	1488 / 1489	10 / 10	1487 / 1489	1488 / 1489	1690 / 1691	1454 / 1454	1701 / 1702	1488 / 1490	9 / 9	1467 / 1468	10 / 10	1488 / 1489	10 / 10	79 / 79	1487 / 1488	1466 / 1467	1494 / 1496	1709 / 1710	1461 / 1461	200 / 200	1446 / 1447	1704 / 1705
BTP 199	1489 / 1489	3 / 3	1488 / 1488	3 / 3	1488 / 1489	2 / 2	6 / 6	1691 / 1691	1348 / 1347	1699 / 1699	9 / 9	1489 / 1489	1392 / 1392	1489 / 1489	11 / 11	1489 / 1489	1487 / 1488	81 / 81	1385 / 1385	248 / 248	1713 / 1713	1420 / 1419	1481 / 1481	1341 / 1341	1701 / 1701
BTP 200	1488 / 1488	1 / 1	1487 / 1487	1 / 1	1487 / 1488	2 / 2	6 / 6	1690 / 1690	1348 / 1346	1698 / 1698	9 / 9	1488 / 1488	1391 / 1391	1488 / 1488	11 / 11	1488 / 1488	1486 / 1487	81 / 81	1384 / 1384	248 / 248	1712 / 1712	1419 / 1418	1480 / 1480	1340 / 1340	1700 / 1700
BTP 201	1489 / 1489	2 / 2	1488 / 1488	2 / 2	1488 / 1489	1 / 1	5 / 5	1691 / 1691	1348 / 1347	1699 / 1699	8 / 8	1489 / 1489	1392 / 1392	1489 / 1489	10 / 10	1489 / 1489	1487 / 1488	80 / 80	1385 / 1385	247 / 247	1713 / 1713	1420 / 1419	1481 / 1481	1341 / 1341	1701 / 1701
BTP 202	1489 / 1488	2 / 2	1488 / 1487	2 / 2	1488 / 1488	1 / 1	5 / 5	1691 / 1690	1348 / 1346	1699 / 1698	8 / 8	1489 / 1488	1392 / 1391	1489 / 1488	10 / 10	1489 / 1488	1487 / 1487	80 / 80	1385 / 1384	247 / 247	1713 / 1712	1420 / 1418	1481 / 1480	1341 / 1340	1701 / 1700
BTP 203	1488 / 1489	3 / 3	1487 / 1488	3 / 3	1487 / 1489	2 / 2	6 / 6	1690 / 1691	1347 / 1347	1698 / 1699	9 / 9	1488 / 1489	1391 / 1392	1488 / 1489	11 / 11	1488 / 1489	1486 / 1488	81 / 81	1384 / 1385	248 / 248	1712 / 1713	1419 / 1419	1480 / 1481	1340 / 1341	1700 / 1701
BTP 204	1487 / 1488	2 / 2	1486 / 1487	2 / 2	1486 / 1488	1 / 1	5 / 5	1689 / 1690	1346 / 1346	1696 / 1698	8 / 8	1487 / 1488	1389 / 1391	1487 / 1488	10 / 10	1487 / 1488	1485 / 1487	80 / 80	1383 / 1384	247 / 248	1710 / 1712	1418 / 1418	1479 / 1480	1339 / 1340	1698 / 1700
BTP 205	1489 / 1488	1 / 1	1488 / 1487	1 / 1	1488 / 1488	2 / 2	6 / 6	1691 / 1690	1348 / 1346	1699 / 1698	9 / 9	1489 / 1488	1392 / 1391	1489 / 1488	11 / 11	1489 / 1488	1487 / 1487	81 / 81	1385 / 1384	248 / 248	1713 / 1712	1420 / 1418	1481 / 1480	1341 / 1340	1701 / 1700
BTP 206	237 / 235	1485 / 1486	238 / 238	1485 / 1486	238 / 238	1485 / 1486	1485 / 1486	1691 / 1693	1454 / 1454	1701 / 1702	1486 / 1487	235 / 235	1463 / 1464	238 / 238	1485 / 1486	238 / 238	208 / 208	1484 / 1485	1461 / 1642	1496 / 1497	1705 / 1707	1455 / 1455	161 / 161	1443 / 1445	1704 / 1705
BTP 207	1488 / 1488	3 / 3	1487 / 1487	3 / 3	1487 / 1488	2 / 2	6 / 6	1690 / 1690	1347 / 1346	1698 / 1698	9 / 9	1488 / 1488	1391 / 1391	1488 / 1488	11 / 11	1488 / 1488	1486 / 1487	81 / 81	1384 / 1384	248 / 248	1713 / 1713	1419 / 1418	1480 / 1480	1340 / 1340	1700 / 1700
BTP 208	286 / 284	1487 / 1487	286 / 286	1487 / 1487	286 / 286	1486 / 1487	1487 / 1487	1693 / 1693	1453 / 1452	1705 / 1705	1487 / 1488	283 / 283	1464 / 1464	286 / 286	1487 / 1487	286 / 286	258 / 258	1486 / 1486	1460 / 1460	1495 / 1496	1709 / 1709	1459 / 1458	222 / 222	1444 / 1444	1708 / 1708
BTP 209	107 / 105	1486 / 1487	108 / 108	1486 / 1487	107 / 107	1486 / 1487	1486 / 1487	1688 / 1689	1451 / 1450	1697 / 1700	1487 / 1488	107 / 107	1462 / 1464	109 / 109	1486 / 1487	109 / 109	59 / 59	1485 / 1486	1463 / 1465	1493 / 1494	1704 / 1706	1462 / 1462	198 / 198	1441 / 1442	1700 / 1703
BTP 210	1488 / 1487	1 / 0	1487 / 1486	1 / 0	1487 / 1487	3 / 2	7 / 6	1689 / 1688	1347 / 1345	1698 / 1697	10 / 9	1488 / 1487	1390 / 1389	1488 / 1487	12 / 11	1488 / 1487	1486 / 1486	82 / 81	1383 / 1382	249 / 248	1711 / 1710	1418 / 1416	1480 / 1479	1340 / 1339	1700 / 1699
BTP 211	1489 / 1489	3 / 3	1488 / 1488	3 / 3	1488 / 1489	0 / 0	6 / 6	1691 / 1691	1348 / 1347	1699 / 1699	9 / 9	1489 / 1489	1392 / 1392	1489 / 1489	11 / 11	1489 / 1489	1487 / 1488	81 / 81	1385 / 1385	248 / 248	1713 / 1713	1420 / 1419	1481 / 1481	1341 / 1341	1701 / 1701
BTP 212	4 / 4	1487 / 1488	1 / 1	1487 / 1488	10 / 10	1486 / 1488	1487 / 1488	1688 / 1690	1455 / 1454	1699 / 1702	1487 / 1489	9 / 9	1465 / 1467	16 / 16	1487 / 1488	16 / 16	84 / 84	1486 / 1487	1465 / 1467	1493 / 1495	1707 / 1709	1461 / 1460	203 / 203	1446 / 1447	1702 / 1705
BTP 213	67 / 65	1488 / 1488	68 / 68	1488 / 1488	67 / 67	1487 / 1488	1488 / 1488	1690 / 1690	1451 / 1450	1701 / 1701	1488 / 1489	66 / 66	1467 / 1467	68 / 68	1488 / 1488	68 / 68	74 / 74	1487 / 1487	1466 / 1466	1494 / 1495	1709 / 1709	1461 / 1460	192 / 192	1444 / 1444	1704 / 1704
BTP 214	1707 / 1707	1708 / 1708	1706 / 1706	1709 / 1709	1704 / 1706	1708 / 1709	1709 / 1709	1661 / 1661	1708 / 1707	1686 / 1686	1708 / 1709	1706 / 1706	1703 / 1703	1706 / 1706	1708 / 1708	1706 / 1706	1708 / 1709	1713 / 1713	1703 / 1703	1711 / 1712	199 / 199	1698 / 1697	1707 / 1701	1710 / 1710	1690 / 1690
BTP 215	1467 / 1466	1370 / 1370	1467 / 1466	1370 / 1370	1466 / 1466	1369 / 1370	1371 / 1371	1683 / 1682	1396 / 1394	1689 / 1688	1371 / 1372	1466 / 1465	1409 / 1408	1470 / 1469	1371 / 1371	1470 / 1469	1464 / 1464	1370 / 1370	1400 / 1399	1372 / 1373	1708 / 1707	1418 / 1416	1466 / 1465	1389 / 1388	1691 / 1690
BTP 216	1708 / 1707	1710 / 1709	1707 / 1706	1711 / 1710	1705 / 1706	1710 / 1710	1711 / 1710	1663 / 1662	1709 / 1707	1685 / 1684	1710 / 1710	1708 / 1707	1704 / 1703	1707 / 1706	1710 / 1709	1707 / 1706	1709 / 1709	1715 / 1714	1704 / 1703	1712 / 1712	233 / 233	1699 / 1697	1708 / 1707	1711 / 1710	1689 / 1688
BTP 217	113 / 111	1481 / 1481	113 / 113	1481 / 1481	113 / 113	1480 / 1481	1481 / 1481	1687 / 1687	1450 / 1449	1697 / 1679	1481 / 1482	112 / 112	1463 / 1463	114 / 114	1481 / 1481	114 / 114	67 / 67	1480 / 1480	1462 / 1462	1487 / 1488	1705 / 1705	1456 / 1455	198 / 198	1441 / 1441	1700 / 1700
BTP 218	116 / 114	1479 / 1481	116 / 116	1479 / 1481	116 / 116	1478 / 1481	1479 / 1481	1686 / 1687	1448 / 1449	1695 / 1697	1479 / 1482	115 / 115	1462 / 1463	117 / 117	1479 / 1481	117 / 117	70 / 70	1478 / 1480	1461 / 1462	1485 / 1488	1704 / 1705	1455 / 1455	201 / 201	1439 / 1441	1698 / 1700
BTP 219	8 / 6	1488 / 1489	9 / 9	1488 / 1489	8 / 8	1487 / 1489	1488 / 1489	1690 / 1691	1454 / 1454	1701 / 1702	1488 / 1490	7 / 7	1467 / 1468	8 / 8	1488 / 1489	8 / 8	78 / 78	1487 / 1488	1466 / 1467	1494 / 1496	1709 / 1710	1461 / 1461	199 / 199	1446 / 1447	1704 / 1705
BTP 220	6 / 4	1490 / 1490	7 / 7	1490 / 1490	6 / 6	1489 / 1490	1490 / 1490	1692 / 1692	1455 / 1454	1703 / 1703	1490 / 1491	5 / 5	1469 / 1469	8 / 8	1490 / 1490	8 / 8	76 / 76	1489 / 1489	1468 / 1468	1496 / 1497	1711 / 1711	1463 / 1462	197 / 197	1447 / 1447	1706 / 1706
BTP 221	6 / 4	1487 / 1489	7 / 7	1487 / 1489	6 / 6	1486 / 1489	1487 / 1489	1690 / 1691	1455 / 1454	1701 / 1702	1487 / 1490	5 / 5	1467 / 1468	8 / 8	1487 / 1489	8 / 8	76 / 76	1486 / 1488	1466 / 1467	1493 / 1496	1709 / 1710	1462 / 1461	197 / 197	1446 / 1447	1704 / 1705
BTP 222	7 / 5	1490 / 1490	8 / 8	1490 / 1490	7 / 7	1489 / 1490	1490 / 1490	1692 / 1692	1455 / 1454	1703 / 1703	1490 / 1491	6 / 6	1469 / 1469	9 / 9	1490 / 1490	9 / 9	75 / 75	1489 / 1489	1468 / 1468	1496 / 1497	1711 / 1711	1463 / 1462	196 / 196	1447 / 1447	1706 / 1706
BTP 223	8 / 6	1486 / 1489	8 / 8	1486 / 1489	8 / 8	1486 / 1489	1486 / 1489	1688 / 1691	1454 / 1454	1699 / 1702	1487 / 1490	7 / 7	1466 / 1468	10 / 10	1486 / 1489	10 / 10	78 / 78	1485 / 1488	1465 / 1467	1493 / 1496	1707 / 1710	1460 / 1461	199 / 199	1445 / 1447	1702 / 1705
BTP 224	1457 / 1456	1348 / 1348	1456 / 1456	1348 / 1348	1453 / 1455	1347 / 1348	1349 / 1349	1690 / 1690	4 / 4	1696 / 1696	1349 / 1350	1456 / 1456	1347 / 1347	1456 / 1456	1350 / 1350	1456 / 1456	1453 / 1454	1344 / 1344	1334 / 1334	1347 / 1348	1712 / 1712	1397 / 1396	1450 / 1450	117 / 117	1698 / 1698
BTP 225	9 / 7	1488 / 1489	10 / 10	1488 / 1489	9 / 9	1488 / 1489	1488 / 1489	1689 / 1691	1454 / 1454	1700 / 1702	1489 / 1490	8 / 8	1466 / 1468	9 / 9	1488 / 1489	9 / 9	78 / 78	1487 / 1488	1465 / 1467	1495 / 1496	1708 / 1710	1461 / 1461	198 / 198	1445 / 1447	1703 / 1705
BTP 226	1488 / 1488	5 / 5	1487 / 1487	5 / 5	1487 / 1488	4 / 4	8 / 8	1690 / 1690	1347 / 1346	1698 / 1698	11 / 11	1488 / 1488	1391 / 1391	1488 / 1488	13 / 13	1488 / 1488	1486 / 1487	78 / 78	1384 / 1384	246 / 246	1712 / 1712	1419 / 1418	1480 / 1480	1340 / 1340	1700 / 1700
BTP 227	1489 / 1489	5 / 5	1488 / 1488	5 / 5	1488 / 1489	4 / 4	8 / 8	1691 / 1691	1348 / 1347	1699 / 1699	11 / 11	1489 / 1489	1392 / 1392	1489 / 1489	13 / 13	1489 / 1489	1487 / 1488	78 / 78	1385 / 1385	246 / 246	1713 / 1713	1420 / 1419	1481 / 1481	1341 / 1341	1701 / 1701
BTP 228	8 / 7	1488 / 1489	9 / 10	1488 / 1489	8 / 9	1487 / 1489	1488 / 1489	1690 / 1691	1454 / 1454	1701 / 1702	1488 / 1490	7 / 8	1467 / 1468	8 / 10	1488 / 1489	8 / 10	77 / 79	1487 / 1488	1466 / 1467	1494 / 1496	1709 / 1710	1462 / 1461	198 / 200	1446 / 1447	1704 / 1705
BTP 229	10 / 8	1491 / 1491	11 / 11	1491 / 1491	10 / 10	1490 / 1491	1491 / 1491	1692 / 1692	1456 / 1455	1703 / 1703	1491 / 1492	9 / 9	1469 / 1469	14 / 14	1491 / 1491	14 / 14	81 / 81	1490 / 1490	1468 / 1468	1497 / 1498	1710 / 1710	1462 / 1461	202 / 202	1448 / 1448	1706 / 1706
BTP 230	9 / 7	1489 / 1489	10 / 10	1489 / 1489	9 / 9	1488 / 1489	1489 / 1489	1691 / 1691	1455 / 1454	1702 / 1702	1489 / 1490	8 / 8	1468 / 1468	9 / 9	1489 / 1489	9 / 9	78 / 78	1488 / 1488	1467 / 1467	1495 / 1496	1710 / 1710	1462 / 1461	199 / 199	1447 / 1447	1705 / 1705
BTP 231	1489 / 1488	4 / 4	1488 / 1487	4 / 4	1488 / 1488	3 / 3	7 / 7	1691 / 1690	1348 / 1346	1699 / 1698	10 / 10	1489 / 1488	1392 / 1391	1489 / 1488	12 / 12	1489 / 1488	1487 / 1487	79 / 79	1385 / 1384	247 / 247	1713 / 1712	1420 / 1418	1481 / 1480	1341 / 1340	1701 / 1700
BTP 232	1490 / 1490	12 / 12	1489 / 1489	12 / 12	1489 / 1490	11 / 11	15 / 15	1691 / 1691	1346 / 1345	1699 / 1699	18 / 18	1490 / 1490	1393 / 1393	1490 / 1490	20 / 20	1490 / 1490	1488 / 1489	77 / 77	1386 / 1386	246 / 246	1713 / 1713	1421 / 1420	1482 / 1482	1340 / 1340	1701 / 1701
BTP 233	10 / 8	1489 / 1489	11 / 11	1489 / 1489	10 / 10	1488 / 1489	1489 / 1489	1691 / 1691	1455 / 1454	1702 / 1702	1489 / 1490	9 / 9	1469 / 1469	8 / 8	1489 / 1489	8 / 8	80 / 80	1488 / 1488	1468 / 1468	1495 / 1496	1710 / 1710	1462 / 1461	200 / 200	1447 / 1447	1705 / 1705
BTP 234	1488 / 1490	11 / 13	1487 / 1489	11 / 13	1487 / 1490	10 / 12	14 / 16	1689 / 1691	1344 / 1345	1697 / 1699	17 / 19	1488 / 1490	1391 / 1393	1488 / 1490	19 / 21	1488 / 1490	1486 / 1489	75 / 77	1384 / 1386	245 / 247	1711 / 1713	1419 / 1420	1480 / 1482	1338 / 1340	1699 / 1701
BTP 235	1486 / 1485	14 / 13	1485 / 1484	14 / 13	1485 / 1485	13 / 12	17 / 16	1687 / 1686	1344 / 1342	1695 / 1694	20 / 19	1486 / 1485	1390 / 1389	1486 / 1485	22 / 21	1486 / 1485	1484 / 1484	78 / 77	1382 / 1381	247 / 246	1709 / 1708	1417 / 1415	1478 / 1477	1338 / 1337	1697 / 1696
BTP 236	1490 / 1490	16 / 16	1489 / 1489	16 / 16	1489 / 1490	15 / 15	19 / 19	1690 / 1690	1345 / 1344	1698 / 1698	22 / 22	1490 / 1490	1393 / 1393	1490 / 1490	24 / 24	1490 / 1490	1487 / 1488	80 / 80	1386 / 1386	250 / 250	1712 / 1712	1420 / 1419	1481 / 1481	1339 / 1339	1700 / 1700
BTP 237	9 / 7	1489 / 1489	10 / 10	1489 / 1489	9 / 9	1488 / 1489	1489 / 1489	1692 / 1692	1456 / 1455	1701 / 1702	1489 / 1490	8 / 8	1470 / 1470	7 / 7	1489 / 1489	7 / 7	79 / 79	1488 / 1488	1469 / 1469	1495 / 1496	1710 / 1710	1462 / 1461	199 / 199	1448 / 1448	1704 / 1705
BTP 238	9 / 7	1489 / 1489	10 / 10	1489 / 1489	9 / 9	1488 / 1489	1489 / 1489	1692 / 1692	1456 / 1455	1702 / 1702	1489 / 1490	8 / 8	1470 / 1470	7 / 7	1489 / 1489	7 / 7	79 / 79	1488 / 1488	1469 / 1469	1495 / 1496	1710 / 1710	1462 / 1461	199 / 199	1448 / 1448	1705 / 1705
BTP 239	13 / 11	1489 / 1489	14 / 14	1489 / 1489	13 / 13	1488 / 1489	1489 / 1489	1690 / 1691	1453 / 1453	1700 / 1701	1489 / 1490	12 / 12	1466 / 1467	15 / 15	1489 / 1489	15 / 15	79 / 79	1489 / 1489	1465 / 1466	1496 / 1497	1708 / 1709	1462 / 1461	199 / 199	1445 / 1446	1703 / 1704
BTP 240	8 / 6	1489 / 1489	9 / 9	1489 / 1489	8 / 8	1488 / 1489	1489 / 1489	1690 / 1690	1455 / 1454	1701 / 1701	1488 / 1489	7 / 7	1468 / 1468	8 / 8	1489 / 1489	8 / 8	77 / 77	1488 / 1488	1467 / 1467	1495 / 1496	1709 / 1709	1462 / 1461	198 / 198	1447 / 1447	1704 / 1704
BTP 241	9 / 7	1489 / 1489	10 / 10	1489 / 1489	9 / 9	1488 / 1489	1489 / 1489	1691 / 1691	1455 / 1454	1702 / 1702	1489 / 1490	8 / 8	1468 / 1468	9 / 9	1489 / 1489	9 / 9	78 / 78	1488 / 1488	1467 / 1467	1495 / 1496	1710 / 1710	1462 / 1461	199 / 199	1447 / 1447	1705 / 1705
BTP 242	0 / 0	1489 / 1489	3 / 3	1489 / 1489	8 / 6	1488 / 1489	1489 / 1489	1691 / 1691	1456 / 1454	1703 / 1703	1489 / 1490	7 / 5	1468 / 1468	14 / 12	1489 / 1489	14 / 12	82 / 80	1488 / 1488	1468 / 1468	1495 / 1496	1710 / 1710	1462 / 1461	202 / 200	1448 / 1447	1706 / 1706
BTP 243	1489 / 1489	0 / 0	1488 / 1488	0 / 0	1488 / 1489	3 / 3	7 / 7	1690 / 1690	1348 / 1347	1698 / 1698	10 / 10	1489 / 1489	1392 / 1392	1489 / 1489	12 / 12	1489 / 1489	1487 / 1488	82 / 82	1385 / 1385	249 / 249	1712 / 1712	1420 / 1419	1481 / 1481	1341 / 1341	1700 / 1700
BTP 244	3 / 3	1488 / 1488	0 / 0	1488 / 1488	9 / 9	1487 / 1488	1488 / 1488	1690 / 1690	1455 / 1454	1702 / 1702	1488 / 1489	8 / 8	1467 / 1467	15 / 15	1488 / 1488	15 / 15	83 / 83	1487 / 1487	1467 / 1467	1494 / 1495	1709 / 1709	1461 / 1460	202 / 202	1447 / 1447	1705 / 1705
BTP 245	1489 / 1489	0 / 0	1488 / 1488	0 / 0	1488 / 1489	3 / 3	7 / 7	1690 / 1690	1348 / 1347	1699 / 1699	10 / 10	1489 / 1489	1392 / 1392	1489 / 1489	12 / 12	1489 / 1489	1487 / 1488	82 / 82	1385 / 1385	249 / 249	1713 / 1713	1420 / 1419	1481 / 1481	1341 / 1341	1701 / 1701
BTP 247	8 / 6	1488 / 1489	9 / 9	1488 / 1489	0 / 0	1487 / 1489	1488 / 1489	1688 / 1690	1452 / 1453	1700 / 1701	1488 / 1490	7 / 7	1466 / 1467	14 / 14	1488 / 1489	14 / 14	82 / 82	1487 / 1488	1465 / 1466	1493 / 1495	1707 / 1709	1462 / 1462	203 / 204	1444 / 1446	1703 / 1704
BTP 248	1488 / 1489	3 / 3	1487 / 1488	3 / 3	1487 / 1489	0 / 0	6 / 6	1690 / 1691	1348 / 1347	1698 / 1699	9 / 9	1488 / 1489	1391 / 1392	1488 / 1489	11 / 11	1488 / 1489	1486 / 1488	81 / 81	1384 / 1385	248 / 248	1712 / 1713	1419 / 1419	1480 / 1481	1340 / 1341	1700 / 1701
BTP 249	1489 / 1489	7 / 7	1488 / 1488	7 / 7	1488 / 1489	6 / 6	0 / 0	1691 / 1691	1349 / 1348	1699 / 1699	3 / 3	1489 / 1489	1393 / 1393	1489 / 1489	5 / 5	1489 / 1489	1487 / 1488	84 / 84	1386 / 1386	251 / 251	1713 / 1713	1421 / 1420	1481 / 1481	1342 / 1342	1701 / 1701
BTP 250	1691 / 1691	1690 / 1690	1690 / 1690	1690 / 1690	1688 / 1690	1690 / 1691	1691 / 1691	0 / 0	1689 / 1688	1688 / 1688	1690 / 1691	1690 / 1690	1690 / 1690	1690 / 1690	1690 / 1690	1690 / 1690	1693 / 1694	1696 / 1696	1688 / 1688	1696 / 1697	1665 / 1665	1698 / 1697	1694 / 1694	1689 / 1689	1690 / 1690
BTP 251	1456 / 1454	1348 / 1347	1455 / 1454	1348 / 1347	1452 / 1453	1348 / 1347	1349 / 1348	1689 / 1688	0 / 0	1695 / 1694	1350 / 1349	1455 / 1454	1347 / 1347	1455 / 1454	1350 / 1349	1455 / 1454	1452 / 1452	1344 / 1343	1334 / 1334	1348 / 1347	1711 / 1710	1396 / 1394	1449 / 1448	119 / 120	1697 / 1696
BTP 252	1703 / 1703	1698 / 1698	1702 / 1702	1699 / 1699	1700 / 1701	1698 / 1699	1699 / 1699	1688 / 1688	1695 / 1694	0 / 0	1698 / 1699	1701 / 1701	1693 / 1693	1701 / 1701	1698 / 1698	1701 / 1701	1703 / 1704	1703 / 1703	1694 / 1694	1703 / 1704	1688 / 1688	1698 / 1697	1704 / 1704	1694 / 1695	88 / 88
BTP 253	1489 / 1490	10 / 10	1488 / 1489	10 / 10	1488 / 1490	9 / 9	3 / 3	1690 / 1691	1350 / 1349	1698 / 1699	0 / 0	1489 / 1490	1394 / 1395	1489 / 1490	6 / 6	1489 / 1490	1487 / 1489	87 / 87	1387 / 1388	254 / 254	1712 / 1713	1421 / 1421	1481 / 1482	1342 / 1343	1700 / 1701
BTP 254	7 / 5	1489 / 1489	8 / 8	1489 / 1489	7 / 7	1488 / 1489	1489 / 1489	1690 / 1690	1455 / 1454	1701 / 1701	1489 / 1490	0 / 0	1468 / 1468	13 / 13	1489 / 1489	13 / 13	81 / 81	1488 / 1488	1467 / 1467	1495 / 1496	1709 / 1709	1461 / 1460	201 / 201	1447 / 1447	1704 / 1704
BTP 255	1468 / 1468	1392 / 1392	1467 / 1467	1392 / 1392	1466 / 1467	1391 / 1392	1393 / 1393	1690 / 1690	1347 / 1347	1693 / 1693	1394 / 1395	1468 / 1468	0 / 0	1469 / 1469	1393 / 1393	1469 / 1469	1465 / 1466	1386 / 1386	211 / 211	1393 / 1394	1706 / 1706	1399 / 1398	1466 / 1466	1340 / 1340	1695 / 1695
BTP 256	14 / 12	1489 / 1489	15 / 15	1489 / 1489	14 / 14	1488 / 1489	1489 / 1489	1690 / 1690	1455 / 1454	1701 / 1701	1489 / 1490	13 / 13	1469 / 1469	0 / 0	1489 / 1489	0 / 0	83 / 83	1488 / 1488	1468 / 1468	1495 / 1496	1709 / 1709	1463 / 1462	203 / 204	1447 / 1447	1704 / 1704
BTP 257	1489 / 1489	12 / 12	1488 / 1488	12 / 12	1488 / 1489	11 / 11	5 / 5	1690 / 1690	1350 / 1349	1698 / 1698	6 / 6	1489 / 1489	1393 / 1393	1489 / 1489	0 / 0	1489 / 1489	1487 / 1488	88 / 88	1386 / 1386	256 / 256	1712 / 1712	1421 / 1420	1481 / 1481	1343 / 1343	1700 / 1700
BTP 258	14 / 12	1489 / 1489	15 / 15	1489 / 1489	14 / 14	1488 / 1489	1489 / 1489	1690 / 1690	1455 / 1454	1701 / 1701	1489 / 1490	13 / 13	1469 / 1469	0 / 0	1489 / 1489	0 / 0	83 / 83	1488 / 1488	1468 / 1468	1495 / 1496	1709 / 1709	1463 / 1462	203 / 203	1447 / 1447	1704 / 1704
USA100	82 / 80	1487 / 1488	83 / 83	1487 / 1488	82 / 82	1486 / 1488	1487 / 1488	1693 / 1694	1452 / 1452	1703 / 1704	1487 / 1489	81 / 81	1465 / 1466	83 / 83	1487 / 1488	83 / 83	0 / 0	1486 / 1487	1464 / 1465	1493 / 1495	1711 / 1712	1461 / 1461	171 / 171	1443 / 1444	1706 / 1707
USA300	1488 / 1488	82 / 82	1487 / 1487	82 / 82	1487 / 1488	81 / 81	84 / 84	1696 / 1696	1344 / 1343	1703 / 1703	87 / 87	1488 / 1488	1386 / 1386	1488 / 1488	88 / 88	1488 / 1488	1486 / 1487	0 / 0	1381 / 1381	209 / 209	1717 / 1717	1420 / 1419	1480 / 1480	1337 / 1337	1705 / 1705
USA400	1468 / 1468	1385 / 1385	1467 / 1467	1385 / 1385	1465 / 1466	1384 / 1385	1386 / 1386	1688 / 1688	1334 / 1334	1694 / 1694	1387 / 1388	1467 / 1467	211 / 211	1468 / 1468	1386 / 1386	1468 / 1468	1464 / 1465	1381 / 1381	0 / 0	1386 / 1387	1706 / 1706	1384 / 1383	1463 / 1463	1328 / 1328	1696 / 1696
USA500	1495 / 1496	249 / 249	1494 / 1495	249 / 249	1493 / 1495	248 / 248	251 / 251	1696 / 1697	1348 / 1347	1703 / 1704	254 / 254	1495 / 1496	1393 / 1394	1495 / 1496	256 / 256	1495 / 1496	1493 / 1495	209 / 209	1386 / 1387	0 / 0	1715 / 1716	1416 / 1416	1488 / 1489	1340 / 1341	1705 / 1706
USA600	1710 / 1710	1712 / 1712	1709 / 1709	1713 / 1713	1707 / 1709	1712 / 1713	1713 / 1713	1665 / 1665	1711 / 1710	1688 / 1688	1712 / 1713	1709 / 1709	1706 / 1706	1709 / 1709	1712 / 1712	1709 / 1709	1711 / 1712	1717 / 1717	1706 / 1706	1715 / 1716	0 / 0	1702 / 1701	1710 / 1710	1713 / 1713	1692 / 1692
USA700	1462 / 1461	1420 / 1419	1461 / 1460	1420 / 1419	1462 / 1462	1419 / 1419	1421 / 1420	1698 / 1697	1396 / 1394	1698 / 1697	1421 / 1421	1461 / 1460	1399 / 1398	1463 / 1462	1421 / 1420	1463 / 1462	1461 / 1461	1420 / 1419	1384 / 1383	1416 / 1416	1702 / 1701	0 / 0	1465 / 1464	1393 / 1392	1700 / 1699
USA800	202 / 200	1481 / 1481	202 / 202	1481 / 1481	203 / 204	1480 / 1481	1481 / 1481	1694 / 1694	1449 / 1448	1704 / 1704	1481 / 1482	201 / 201	1466 / 1466	203 / 204	1481 / 1481	203 / 203	171 / 171	1480 / 1480	1463 / 1463	1488 / 1489	1710 / 1710	1465 / 1464	0 / 0	1439 / 1439	1707 / 1707
USA900	1448 / 1447	1341 / 1341	1447 / 1447	1341 / 1341	1444 / 1446	1340 / 1341	1342 / 1342	1689 / 1689	119 / 120	1694 / 1695	1342 / 1343	1447 / 1447	1340 / 1340	1447 / 1447	1343 / 1343	1447 / 1447	1443 / 1444	1337 / 1337	1328 / 1328	1340 / 1341	1713 / 1713	1393 / 1392	1439 / 1439	0 / 0	1697 / 1698
USA1000	1706 / 1706	1700 / 1700	1705 / 1705	1701 / 1701	1703 / 1704	1700 / 1701	1701 / 1701	1690 / 1690	1697 / 1696	88 / 88	1700 / 1701	1704 / 1704	1695 / 1695	1704 / 1704	1700 / 1700	1704 / 1704	1706 / 1707	1705 / 1705	1696 / 1696	1705 / 1706	1692 / 1692	1700 / 1699	1707 / 1707	1697 / 1698	0 / 0

≤8 related (green), 9-29 possibly related (yellow), ≥30 unrelated (red)

Supplemental Table 3: *Acinetobacter baumannii* Allelic Differences Between Isolates

Manual WGS Method

Automated WGS Method

**Isolate**	**ARLG-1256**	**ARLG-1257**	**ARLG-1262**	**ARLG-1263**	**ARLG-1264**	**ARLG-1266**	**ARLG-1275**	**ARLG-1278**
**ARLG-1256**	0 / 0	26 / 26	52 / 54	54 / 52	65 / 65	2 / 2	2207 / 2209	2206 / 2208
**ARLG-1257**	26 / 26	0 / 0	38 / 40	40 / 38	51 / 51	24 / 24	2208 / 2210	2207 / 2209
**ARLG-1262**	52 / 54	38 / 40	0 / 0	7 / 7	19 / 17	50 / 52	2209 / 2209	2208 / 2208
**ARLG-1263**	54 / 52	40 / 38	7 / 7	0 / 0	17 / 19	52 / 50	2209 / 2210	2208 / 2209
**ARLG-1264**	65 / 65	51 / 51	19 / 17	17 / 17	0 / 0	63 / 63	2196 / 2212	2195 / 2211
**ARLG-1266**	2 / 2	24 / 24	50 / 52	52 / 52	63 / 63	0 / 0	2209 / 2208	2208 / 2207
**ARLG-1275**	2207 / 2209	2208 / 2210	2209 / 2209	2209 / 2209	2196 / 2212	2209 / 2208	0 / 0	2 / 2
**ARLG-1278**	2206 / 2208	2207 / 2209	2208 / 2208	2208 / 2208	2195 / 2211	2208 / 2207	2 / 2	0 / 0

≤9 related (green), 10-200 possibly related (yellow), ≥201 unrelated (red)

Supplemental Table 4: *Klebsiella pneumoniae* Allelic Differences Between Isolates

Manual WGS Method

Automated WGS Method

**Isolate**	**ARLG-1159**	**ARLG-1163**	**ARLG-1164**	**ARLG-1167**	**ARLG-1168**	**ARLG-1169**	**ARLG-1719**	**ARLG-1720**
**ARLG-1159**	0 / 0	38 / 38	1912 / 1911	27 / 27	44 / 44	1872 / 1871	24 / 24	22 / 22
**ARLG-1163**	38 / 38	0 / 0	1915 / 1913	42 / 42	25 / 25	1872 / 1870	39 / 39	37 / 37
**ARLG-1164**	1912 / 1911	1915 / 1913	0 / 0	1911 / 1910	1912 / 1915	1938 / 1936	1912 / 1911	1911 / 1910
**ARLG-1167**	27 / 27	42 / 42	1911 / 1910	0 / 0	47 / 47	1869 / 1868	7 / 7	5 / 5
**ARLG-1168**	44 / 44	25 / 25	1912 / 1915	47 / 47	0 / 0	1870 / 1873	44 / 44	42 / 42
**ARLG-1169**	1872 / 1871	1872 / 1870	1938 / 1936	1869 / 1868	1870 / 1873	0 / 0	1870 / 1869	1869 / 1868
**ARLG-1719**	24 / 24	39 / 39	1912 / 1911	7 / 7	44 / 44	1870 / 1869	0 / 0	2 / 2
**ARLG-1720**	22 / 22	37 / 37	1911 / 1910	5 / 5	42 / 42	1869 / 1868	2 / 2	0 / 0

≤15 related (green), 16-50 possibly related (yellow), ≥51 unrelated (red)

Supplemental Table 5: *Legionella pneumophila* Allelic Differences Between Isolates

Manual WGS Method

Automated WGS Method

**Isolate**	**MBRL 1495**	**MBRL 1496**	**MBRL 1497**	**MBRL 1498**	**MBRL 1499**	**MBRL 1504**	**MBRL 1508**	**MBRL 1509**
**MBRL 1495**	0 / 0	1402 / 1402	1403 / 1403	1349 / 1350	1347 / 1347	27 / 27	3 / 3	1403 / 1403
**MBRL 1496**	1402 / 1402	0 / 0	1 / 1	1438 / 1439	1438 / 1438	1404 / 1404	1403 / 1402	14 / 14
**MBRL 1497**	1403 / 1403	1 / 1	0 / 0	1439 / 1440	1439 / 1439	1405 / 1405	1404 / 1403	15 / 15
**MBRL 1498**	1349 / 1350	1438 / 1439	1439 / 1440	0 / 0	66 / 66	1353 / 1354	1349 / 1349	1440 / 1441
**MBRL 1499**	1347 / 1347	1438 / 1438	1439 / 1439	66 / 66	0 / 0	1351 / 1351	1347 / 1346	1440 / 1440
**MBRL 1504**	27 / 27	1404 / 1404	1405 / 1405	1353 / 1354	1351 / 1351	0 / 0	24 / 24	1405 / 1405
**MBRL 1508**	3 / 3	1403 / 1402	1404 / 1403	1349 / 1349	1347 / 1346	24 / 24	0 / 0	1404 / 1403
**MBRL 1509**	1403 / 1403	14 / 14	15 / 15	1440 / 1441	1440 / 1440	1405 / 1405	1404 / 1403	0 / 0

≤4 related (green), 5-30 possibly related (yellow), ≥31 unrelated (red)

Supplemental Table 6: *Clostridioides difficile* Allelic Differences Between Isolates

Manual WGS Method

Automated WGS Method

**Isolate**	**28814**	**29666**	**29770**	**30503**	**30506**	**30516**	**30533**	**30535**
**28814**	0<br>0	1568<br>1568	1539<br>1539	5<br>5	1449<br>1447	1524<br>1522	1435<br>1449	1523<br>1522
**29666**	1568<br>1568	0<br>0	1558<br>1562	1569<br>1572	1419<br>1422	1566<br>1569	1404<br>1425	1565<br>1569
**29770**	1539<br>1539	1558<br>1562	0<br>0	1542<br>1544	1460<br>1462	348<br>348	1442<br>1462	347<br>348
**30503**	5<br>5	1569<br>1572	1542<br>1544	0<br>0	1450<br>1451	1527<br>1527	1436<br>1453	1526<br>1527
**30506**	1449<br>1447	1419<br>1422	1460<br>1462	1450<br>1451	0<br>0	1440<br>1441	55<br>56	1439<br>1441
**30516**	1524<br>1522	1566<br>1569	348<br>348	1527<br>1527	1440<br>1441	0<br>0	1423<br>1441	0<br>0
**30533**	1435<br>1449	1404<br>1425	1442<br>1462	1436<br>1453	55<br>56	1423<br>1441	0<br>0	1422<br>1441
**30535**	1523<br>1522	1565<br>1569	347<br>348	1526<br>1527	1439<br>1441	0<br>0	1422<br>1441	0<br>0

≤6 related (green), 7-50 possibly related (yellow), ≥51 unrelated (red)

Supplemental Table 7: *Escherichia coli* Allelic Differences Between Isolates

Manual WGS Method

Automated WGS Method

**Isolate**	**MBRL 2159**	**MBRL 2160**	**MBRL 2378**	**MBRL 2409**	**MBRL 2410**	**MBRL 2411**	**MBRL 2412**	**MBRL 2461**
**MBRL 2159**	0 / 0	0 / 0	2374 / 2375	2374 / 2374	2374 / 2375	2374 / 2375	2374 / 2375	2376 / 2377
**MBRL 2160**	0 / 0	0 / 0	2375 / 2375	2375 / 2374	2375 / 2375	2375 / 2375	2375 / 2375	2377 / 2377
**MBRL 2378**	2374 / 2375	2375 / 2375	0 / 0	45 / 45	45 / 45	45 / 45	46 / 46	45 / 45
**MBRL 2409**	2374 / 2374	2375 / 2374	45 / 45	0 / 0	0 / 0	1 / 1	1 / 1	42 / 42
**MBRL 2410**	2374 / 2375	2375 / 2375	45 / 45	0 / 0	0 / 0	1 / 1	1 / 1	42 / 42
**MBRL 2411**	2374 / 2375	2375 / 2375	45 / 45	1 / 1	1 / 1	0 / 0	2 / 2	42 / 42
**MBRL 2412**	2374 / 2375	2375 / 2375	46 / 46	1 / 1	1 / 1	2 / 2	0 / 0	43 / 43
**MBRL 2461**	2376 / 2377	2377 / 2377	45 / 45	42 / 42	42 / 42	42 / 42	43 / 43	0 / 0

≤10 related (green), 11-30 possibly related (yellow), ≥31 unrelated (red)

Supplemental Table 8: *Enterobacter cloacae* complex Allelic Differences Between Isolates

Manual WGS Method (columns); **Automated WGS Method** (rows). Each cell shows two values split by a diagonal: upper right / lower left.

**Isolate**	**MBRL 2471**	**MBRL 2472**	**MBRL 2473**	**MBRL 2474**
**MBRL 2471**	0 / 0	2356 / 2355	2356 / 2355	0 / 0
**MBRL 2472**	2356 / 2355	0 / 0	0 / 0	2356 / 2355
**MBRL 2473**	2356 / 2355	0 / 0	0 / 0	2356 / 2355
**MBRL 2474**	0 / 0	2356 / 2355	2356 / 2355	0 / 0

≤15 related (green), 16-50 possibly related (yellow), ≥51 unrelated (red)

Supplemental Table 9: *Campylobacter jejuni/coli* Allelic Differences Between Isolates

Manual WGS Method (columns); **Automated WGS Method** (rows)

Isolate	1	3	5	11	13	19	21	25	27	29	41	51	52	71	84	96
**1**	0 / 0	560 / 560	594 / 596	26 / 27	23 / 23	578 / 577	588 / 588	576 / 578	578 / 577	580 / 581	578 / 578	582 / 581	578 / 578	580 / 599	601 / 601	577 / 581
**3**	560 / 560	0 / 0	585 / 587	561 / 561	560 / 561	450 / 449	602 / 602	581 / 583	582 / 581	267 / 266	450 / 450	269 / 267	450 / 450	584 / 569	584 / 584	582 / 598
**5**	594 / 596	585 / 587	0 / 0	592 / 594	592 / 595	562 / 563	594 / 596	570 / 574	575 / 577	576 / 579	562 / 564	578 / 579	562 / 564	580 / 549	532 / 534	576 / 597
**11**	26 / 27	561 / 561	592 / 594	0 / 0	23 / 24	579 / 578	587 / 587	575 / 577	577 / 576	584 / 585	579 / 579	586 / 585	579 / 579	579 / 598	600 / 600	576 / 581
**13**	23 / 23	560 / 561	592 / 595	23 / 24	0 / 0	575 / 575	586 / 587	574 / 577	576 / 576	584 / 586	575 / 576	586 / 586	575 / 576	578 / 598	599 / 600	575 / 579
**19**	578 / 577	450 / 449	562 / 563	579 / 578	575 / 575	0 / 0	599 / 598	580 / 581	585 / 583	419 / 419	1 / 1	420 / 419	2 / 1	587 / 561	574 / 573	585 / 596
**21**	588 / 588	602 / 602	594 / 596	587 / 587	586 / 587	599 / 598	0 / 0	577 / 579	577 / 576	597 / 598	599 / 599	600 / 599	599 / 599	580 / 602	602 / 602	576 / 587
**25**	576 / 578	581 / 583	570 / 574	575 / 577	574 / 577	580 / 581	577 / 579	0 / 0	379 / 278	579 / 582	580 / 582	581 / 582	580 / 582	407 / 580	579 / 581	374 / 575
**27**	578 / 577	582 / 581	575 / 577	577 / 576	576 / 576	585 / 583	577 / 576	379 / 378	0 / 0	582 / 582	585 / 584	584 / 582	585 / 584	353 / 581	581 / 580	278 / 574
**29**	580 / 581	267 / 266	576 / 579	584 / 585	584 / 586	419 / 419	597 / 598	579 / 582	582 / 582	0 / 0	419 / 420	7 / 6	419 / 420	583 / 576	584 / 585	582 / 598
**41**	578 / 578	450 / 450	562 / 564	579 / 579	575 / 576	1 / 1	599 / 599	580 / 582	585 / 584	419 / 420	0 / 0	420 / 420	1 / 1	587 / 562	574 / 574	585 / 597
**51**	582 / 581	269 / 267	578 / 579	586 / 585	586 / 586	420 / 419	600 / 599	581 / 582	584 / 582	7 / 6	420 / 420	0 / 0	420 / 420	585 / 576	585 / 584	584 / 598
**52**	578 / 578	450 / 450	562 / 564	579 / 579	575 / 576	2 / 1	599 / 599	580 / 582	585 / 584	419 / 420	1 / 1	420 / 420	0 / 0	587 / 562	574 / 574	585 / 597
**71**	580 / 599	584 / 569	580 / 549	579 / 598	578 / 598	587 / 561	580 / 602	407 / 580	353 / 581	583 / 576	587 / 562	585 / 576	587 / 562	0 / 0	583 / 278	354 / 603
**84**	601 / 601	584 / 584	532 / 534	600 / 600	599 / 600	574 / 573	602 / 602	579 / 581	581 / 580	584 / 585	574 / 574	585 / 584	574 / 574	583 / 278	0 / 0	582 / 602
**96**	577 / 581	582 / 598	576 / 597	576 / 581	575 / 579	585 / 596	576 / 587	374 / 575	278 / 574	582 / 598	585 / 597	584 / 598	585 / 597	354 / 603	582 / 602	0 / 0

≤10 related (green), 11-60 possibly related (yellow), ≥61 unrelated (red)

Supplemental Table 10: *Enterococcus faecium* Allelic Differences Between Isolates

Manual WGS Method

Automated WGS Method (rows)

Isolate	MBRL 2116	MBRL 2117	MBRL 2118	MBRL 2119	MBRL 2124	MBRL 2125	MBRL 2126	MBRL 2128	MBRL 2129	MBRL 2130	MBRL 2135	MBRL 2143
**MBRL 2116**	0 / 0	213 / 213	213 / 213	15 / 15	19 / 19	316 / 317	181 / 181	226 / 226	225 / 226	226 / 227	246 / 265	265 / 265
**MBRL 2117**	213 / 213	0 / 0	1 / 1	202 / 202	206 / 206	194 / 194	85 / 85	274 / 274	274 / 274	274 / 275	61 / 73	73 / 73
**MBRL 2118**	213 / 213	0 / 1	0 / 0	202 / 202	206 / 206	194 / 194	84 / 84	275 / 275	275 / 275	275 / 276	60 / 72	72 / 72
**MBRL 2119**	15 / 15	202 / 202	202 / 202	0 / 0	6 / 6	311 / 311	172 / 172	218 / 218	217 / 218	218 / 219	237 / 256	256 / 256
**MBRL 2124**	19 / 19	206 / 206	206 / 206	6 / 6	0 / 0	315 / 315	176 / 176	220 / 221	219 / 221	221 / 222	241 / 260	260 / 260
**MBRL 2125**	316 / 317	194 / 194	194 / 194	311 / 311	315 / 315	0 / 0	192 / 192	347 / 346	346 / 246	347 / 348	234 / 252	252 / 252
**MBRL 2126**	181 / 181	84 / 85	84 / 84	172 / 172	176 / 176	192 / 192	0 / 0	290 / 290	290 / 290	290 / 291	124 / 141	141 / 141
**MBRL 2128**	226 / 226	275 / 274	275 / 275	218 / 218	220 / 221	347 / 346	290 / 290	0 / 0	0 / 0	1 / 2	308 / 325	325 / 325
**MBRL 2129**	225 / 226	275 / 274	275 / 275	217 / 218	219 / 221	346 / 346	290 / 290	0 / 0	0 / 0	1 / 2	308 / 325	325 / 325
**MBRL 2130**	226 / 227	275 / 275	275 / 276	218 / 219	221 / 222	347 / 348	290 / 291	1 / 2	1 / 2	0 / 0	307 / 326	325 / 326
**MBRL 2135**	246 / 265	60 / 73	60 / 72	237 / 256	241 / 260	234 / 252	124 / 141	308 / 325	308 / 325	307 / 326	0 / 0	0 / 0
**MBRL 2143**	265 / 265	72 / 73	72 / 72	256 / 256	260 / 260	252 / 252	141 / 141	325 / 325	325 / 325	325 / 326	0 / 0	0 / 0

≤7 related (green), 8-30 possibly related (yellow), ≥31 unrelated (red)

Supplemental Table 11: *Enterococcus faecalis* Allelic Differences Between Isolates

Manual WGS Method

Automated WGS Method

**Isolate**	**MBRL 2112**	**MBRL 2114**	**MBRL 2120**	**MBRL 2467**	**MBRL 2468**	**MBRL 2469**	**MBRL 2470**
**MBRL 2112**	0<br>0	48<br>48	49<br>49	1723<br>1719	1614<br>1613	1614<br>1613	1614<br>1613
**MBRL 2114**	48<br>48	0<br>0	4<br>4	1722<br>1718	1610<br>1609	1610<br>1609	1610<br>1609
**MBRL 2120**	49<br>49	4<br>0	0<br>0	1722<br>1718	1610<br>1609	1610<br>1609	1610<br>1609
**MBRL 2467**	1723<br>1719	1722<br>1718	1722<br>1718	0<br>0	1627<br>1623	1627<br>1622	1627<br>1623
**MBRL 2468**	1614<br>1613	1610<br>1609	1610<br>1609	1627<br>1623	0<br>0	0<br>0	0<br>1
**MBRL 2469**	1614<br>1613	1610<br>1609	1610<br>1609	1627<br>1622	0<br>0	0<br>0	0<br>0
**MBRL 2470**	1614<br>1613	1610<br>1609	1610<br>1609	1627<br>1623	0<br>1	0<br>0	0<br>0

≤7 related (green), 8-30 possibly related (yellow), ≥31 unrelated (red)

Supplemental Table 12: *Streptococcus pyogenes* Allelic Differences Between Isolates

Manual WGS Method (columns); **Automated WGS Method** (rows)

Isolate	MBRL 1452	MBRL 1455	MBRL 1456	MBRL 1458	MBRL 1466	MBRL 1472	MBRL 1473	MBRL 1475	MBRL 1477	MBRL 1478	MBRL 1482	MBRL 1484
**MBRL 1452**	0 / 0	45 / 45	988 / 988	988 / 988	32 / 32	29 / 29	32 / 32	1010 / 1011	24 / 24	1009 / 1011	965 / 965	997 / 1016
**MBRL 1455**	45 / 45	0 / 0	986 / 986	986 / 986	41 / 41	38 / 38	45 / 45	1005 / 1005	49 / 49	1004 / 1006	959 / 959	995 / 1014
**MBRL 1456**	988 / 988	986 / 986	0 / 0	5 / 5	986 / 986	981 / 986	985 / 988	1006 / 1006	986 / 986	1004 / 1006	966 / 966	980 / 997
**MBRL 1458**	988 / 988	986 / 986	5 / 5	0 / 0	986 / 986	981 / 986	985 / 988	1006 / 1006	986 / 986	1004 / 1006	966 / 966	980 / 997
**MBRL 1466**	32 / 32	41 / 41	986 / 986	986 / 986	0 / 0	3 / 3	33 / 33	1007 / 1007	37 / 37	1006 / 1008	962 / 962	994 / 1013
**MBRL 1472**	29 / 29	38 / 38	981 / 986	981 / 986	3 / 3	0 / 0	30 / 30	1001 / 1007	34 / 34	1002 / 1008	955 / 961	988 / 1013
**MBRL 1473**	32 / 32	45 / 45	985 / 988	985 / 988	33 / 33	30 / 30	0 / 0	1005 / 1009	36 / 36	1005 / 1010	958 / 962	994 / 1016
**MBRL 1475**	1010 / 1010	1005 / 1005	1006 / 1006	1006 / 1006	1007 / 1007	1001 / 1007	1005 / 1009	0 / 0	1008 / 1008	16 / 16	979 / 979	985 / 1003
**MBRL 1477**	24 / 24	49 / 49	986 / 986	986 / 986	37 / 37	34 / 34	36 / 36	1008 / 1008	0 / 0	1007 / 1009	962 / 962	997 / 1015
**MBRL 1478**	1009 / 1011	1004 / 1006	1004 / 1006	1004 / 1006	1006 / 1008	1002 / 1008	1005 / 1010	16 / 16	1007 / 1009	0 / 0	978 / 980	983 / 1003
**MBRL 1482**	965 / 965	959 / 959	966 / 966	966 / 966	962 / 962	955 / 961	958 / 962	979 / 979	962 / 962	978 / 980	0 / 0	964 / 982
**MBRL 1484**	997 / 1016	995 / 1014	980 / 997	980 / 997	994 / 1013	988 / 1013	994 / 1016	985 / 1003	997 / 1015	983 / 1003	964 / 982	0 / 0

≤20 related (green), 21-100 possibly related (yellow), ≥101 unrelated (red)

Supplemental Table 13: *Serratia marcescens* Allelic Differences Between Isolates

Manual WGS Method

Automated WGS Method

Isolate	MBRL 2209	MBRL 2212	MBRL 2219	MBRL 2221	MBRL 2383	MBRL 2386	MBRL 2391	MBRL 2395
**MBRL 2209**	0 / 0	0 / 0	3142 / 3143	3148 / 3149	3147 / 3149	3101 / 3103	3132 / 3132	3132 / 3133
**MBRL 2212**	0 / 0	0 / 0	3143 / 3143	3149 / 3149	3148 / 3149	3102 / 3103	3133 / 3132	3133 / 3133
**MBRL 2219**	3142 / 3143	3143 / 3143	0 / 0	3135 / 3135	3134 / 3135	3082 / 3082	3128 / 3127	3128 / 3128
**MBRL 2221**	3148 / 3149	3149 / 3149	3135 / 3135	0 / 0	0 / 0	3098 / 3099	3120 / 3119	3120 / 3120
**MBRL 2383**	3147 / 3149	3148 / 3149	3134 / 3135	0 / 0	0 / 0	3097 / 3099	3119 / 3119	3119 / 3120
**MBRL 2386**	3101 / 3103	3102 / 3103	3082 / 3082	3098 / 3099	3097 / 3099	0 / 0	3087 / 3088	3087 / 3088
**MBRL 2391**	3132 / 3132	3133 / 3132	3128 / 3127	3120 / 3119	3119 / 3119	3087 / 3088	0 / 0	0 / 0
**MBRL 2395**	3132 / 3133	3133 / 3133	3128 / 3128	3120 / 3120	3119 / 3120	3087 / 3088	0 / 0	0 / 0

≤12 related (green), 13-100 possibly related (yellow), ≥101 unrelated (red)

Supplemental Table 14: *Pseudomonas aeruginosa* Allelic Differences Between Isolates

Manual WGS Method

Automated WGS Method

Isolate	MBRL 2368	MBRL 2369	MBRL 2423	MBRL 2424	MBRL 2425	MBRL 2427	MBRL 2428	MBRL 2431	MBRL 2436	MBRL 2439
**MBRL 2368**	0 / 0	3764 / 3761	3734 / 3734	3274 / 3280	3742 / 3741	3469 / 3473	3665 / 3669	3472 / 3474	3712 / 3711	3733 / 3735
**MBRL 269**	3764 / 3761	0 / 0	3527 / 3526	3763 / 3765	3535 / 3532	3689 / 3690	3464 / 3465	3691 / 3691	3506 / 3503	3527 / 3526
**MBRL 2423**	3734 / 3734	3527 / 3526	0 / 0	3742 / 3745	62 / 62	3673 / 3677	66 / 65	3675 / 3676	80 / 80	6 / 6
**MBRL 2424**	3274 / 3280	3763 / 3765	3742 / 3745	0 / 0	3746 / 3752	3466 / 3474	3673 / 3681	3468 / 3475	3714 / 3720	3740 / 3747
**MBRL 2425**	3742 / 3741	3535 / 3532	62 / 62	3746 / 3752	0 / 0	3678 / 3684	110 / 110	3680 / 3683	68 / 68	62 / 62
**MBRL 2427**	3469 / 3473	3689 / 3690	3673 / 3677	3466 / 3474	3678 / 3684	0 / 0	3577 / 3583	2 / 2	3648 / 3653	3672 / 3677
**MBRL 2428**	3665 / 3669	3464 / 3465	66 / 65	3673 / 3681	110 / 110	3577 / 3583	0 / 0	3576 / 3583	121 / 121	64 / 64
**MBRL 2431**	3472 / 3474	3691 / 3691	3675 / 3676	3468 / 3475	3680 / 3683	2 / 2	3576 / 3583	0 / 0	3650 / 3653	3672 / 3677
**MBRL 2436**	3712 / 3711	3506 / 3503	80 / 80	3714 / 3720	68 / 68	3648 / 3653	121 / 121	3650 / 3653	0 / 0	79 / 79
**MBRL 2439**	3733 / 3735	3527 / 3526	6 / 6	3740 / 3747	62 / 62	3672 / 3677	64 / 64	3672 / 3677	79 / 79	0 / 0

≤6 related (green), 7-100 possibly related (yellow), ≥101 unrelated (red)

Supplemental Table 15: *Streptococcus agalactiae* Allelic Differences Between Isolates

Manual WGS Method

Automated WGS Method

**Isolate**	**MBRL 2758**	**MBRL 2759**	**MBRL 2760**	**MBRL 2761**	**MBRL 2762**	**MBRL 2763**	**MBRL 2764**	**MBRL 2765**	**MBRL 2814**
**MBRL 2758**	0 / 0	0 / 0	866 / 870	0 / 0	884 / 887	0 / 0	0 / 0	0 / 0	1069 / 1072
**MBRL 2759**	0 / 0	0 / 0	868 / 870	0 / 0	885 / 887	0 / 0	0 / 0	0 / 0	1069 / 1072
**MBRL 2760**	866 / 870	868 / 870	0 / 0	865 / 869	272 / 272	868 / 870	868 / 870	867 / 870	1082 / 1085
**MBRL 2761**	0 / 0	0 / 0	865 / 869	0 / 0	884 / 886	0 / 0	0 / 0	0 / 0	1068 / 1071
**MBRL 2762**	884 / 887	885 / 887	272 / 272	884 / 886	0 / 0	886 / 887	886 / 887	885 / 887	1083 / 1085
**MBRL 2763**	0 / 0	0 / 0	868 / 870	0 / 0	886 / 887	0 / 0	0 / 0	0 / 0	1070 / 1072
**MBRL 2764**	0 / 0	0 / 0	868 / 870	0 / 0	886 / 887	0 / 0	0 / 0	0 / 0	1070 / 1072
**MBRL 2765**	0 / 0	0 / 0	867 / 870	0 / 0	885 / 887	0 / 0	0 / 0	0 / 0	1069 / 1072
**MBRL 2814**	1069 / 1072	1069 / 1072	1082 / 1085	1068 / 1071	1083 / 1085	1070 / 1072	1070 / 1072	1069 / 1072	0 / 0

≤20 related (green), 21-100 possibly related (yellow), ≥101 unrelated (red)

Supplemental Table 16: *Staphylococcus lugdunensis* Allelic Differences Between Isolates

Manual WGS Method

Automated WGS Method

**Isolate**	**MBRL 2772**	**MBRL 2773**	**MBRL 2774**	**MBRL 2775**	**MBRL 2776**	**MBRL 2799**	**MBRL 2800**	**MBRL 2801**	**MBRL 2802**	**MBRL 2803**	**MBRL 2804**	**MBRL 2810**
**MBRL 2772**	0<br>0	1572<br>1574	1547<br>1547	1531<br>1540	1551<br>1552	1558<br>1558	1552<br>1555	1549<br>1555	1554<br>1553	1552<br>1556	1553<br>1571	1555<br>1555
**MBRL 2773**	1572<br>1574	0<br>0	1566<br>1569	1574<br>1584	1562<br>1565	1569<br>1572	1566<br>1572	1558<br>1568	1563<br>1565	1563<br>1569	1573<br>1593	1570<br>1572
**MBRL 2774**	1547<br>1547	1566<br>1569	0<br>0	1325<br>1336	1343<br>1346	249<br>249	272<br>273	248<br>250	218<br>218	266<br>266	1371<br>1390	273<br>273
**MBRL 2775**	1531<br>1540	1574<br>1584	1325<br>1336	0<br>0	1372<br>1380	1334<br>1342	1337<br>1348	1331<br>1343	1329<br>1336	1334<br>1343	1445<br>1468	1340<br>1348
**MBRL 2776**	1551<br>1552	1562<br>1565	1343<br>1346	1372<br>1380	0<br>0	1343<br>1345	1342<br>1347	1336<br>1344	1340<br>1341	1345<br>1350	1173<br>1190	1344<br>1346
**MBRL 2799**	1558<br>1558	1569<br>1572	249<br>249	1334<br>1342	1343<br>1345	0<br>0	312<br>313	147<br>148	161<br>161	305<br>305	1375<br>1395	313<br>313
**MBRL 2800**	1552<br>1555	1566<br>1572	272<br>273	1337<br>1348	1342<br>1347	312<br>313	0<br>0	309<br>310	276<br>277	181<br>182	1376<br>1397	3<br>3
**MBRL 2801**	1549<br>1555	1558<br>1568	248<br>250	1331<br>1343	1336<br>1344	147<br>148	309<br>310	0<br>0	159<br>160	305<br>305	1364<br>1385	310<br>310
**MBRL 2802**	1554<br>1553	1563<br>1565	218<br>218	1329<br>1336	1340<br>1341	161<br>161	276<br>277	159<br>160	0<br>0	273<br>272	1360<br>1378	277<br>277
**MBRL 2803**	1552<br>1556	1563<br>1569	266<br>266	1334<br>1343	1345<br>1350	305<br>305	181<br>182	305<br>305	273<br>272	0<br>0	1376<br>1397	182<br>182
**MBRL 2804**	1553<br>1571	1573<br>1593	1371<br>1390	1445<br>1468	1173<br>1190	1375<br>1395	1376<br>1397	1364<br>1385	1360<br>1378	1376<br>1397	0<br>0	1377<br>1396
**MBRL 2810**	1555<br>1555	1570<br>1572	273<br>273	1340<br>1348	1344<br>1346	313<br>313	3<br>3	310<br>310	277<br>277	182<br>182	1377<br>1369	0<br>0

≤8 related (green), 9-29 possibly related (yellow), ≥30 unrelated (red)

Supplemental Table 17: *Staphylococcus epidermidis* Allelic Differences Between Isolates

Manual WGS Method

Automated WGS Method

	**MBRL 2782**	**MBRL 2783**	**MBRL 2784**	**MBRL 2785**	**MBRL 2786**	**MBRL 2787**	**MBRL 2788**	**MBRL 2789**	**MBRL 2790**	**MBRL 2792**	**MBRL 2793**	**MBRL 2794**
**MBRL 2782**	0 / 0	787 / 795	774 / 783	758 / 766	754 / 764	795 / 440	757 / 804	791 / 804	771 / 804	792 / 804	791 / 804	790 / 804
**MBRL 2783**	787 / 795	0 / 0	4 / 5	929 / 930	928 / 933	63 / 22	52 / 60	907 / 909	883 / 909	908 / 909	905 / 909	905 / 909
**MBRL 2784**	774 / 783	4 / 5	0 / 0	915 / 919	915 / 922	64 / 23	53 / 62	897 / 900	874 / 900	897 / 900	895 / 900	894 / 900
**MBRL 2785**	758 / 766	929 / 930	915 / 919	0 / 0	46 / 46	932 / 570	883 / 933	986 / 986	960 / 986	987 / 986	983 / 986	984 / 986
**MBRL 2786**	754 / 764	928 / 933	915 / 922	46 / 46	0 / 0	931 / 570	884 / 935	980 / 985	957 / 985	980 / 985	978 / 985	979 / 985
**MBRL 2787**	795 / 440	63 / 22	64 / 23	932 / 570	931 / 570	0 / 0	9 / 8	910 / 621	885 / 621	910 / 621	907 / 621	908 / 621
**MBRL 2788**	757 / 803	52 / 60	53 / 62	883 / 933	884 / 935	9 / 8	0 / 0	855 / 911	839 / 911	855 / 911	853 / 911	854 / 911
**MBRL 2789**	791 / 804	907 / 909	897 / 900	986 / 986	980 / 985	910 / 621	855 / 911	0 / 0	1 / 1	1 / 1	1 / 1	0 / 0
**MBRL 2790**	771 / 804	883 / 909	874 / 900	960 / 986	957 / 985	885 / 621	839 / 911	1 / 1	0 / 0	1 / 1	1 / 1	1 / 1
**MBRL 2792**	792 / 804	908 / 909	897 / 900	987 / 986	980 / 985	910 / 621	855 / 911	1 / 1	1 / 1	0 / 0	0 / 0	1 / 1
**MBRL 2793**	791 / 804	905 / 909	895 / 900	983 / 986	978 / 985	907 / 621	853 / 911	1 / 1	1 / 1	0 / 0	0 / 0	1 / 1
**MBRL 2794**	790 / 804	905 / 909	894 / 900	984 / 986	979 / 985	908 / 621	854 / 911	0 / 0	1 / 1	1 / 1	1 / 1	0 / 0

≤8 related (green), 9-29 possibly related (yellow), ≥30 unrelated (red)

Supplemental Table 18: *Cutibacterium acnes* Allelic Differences Between Isolates

Manual WGS Method

Automated WGS Method

**Isolate**	**MBRL 2834**	**MBRL 2835**	**MBRL 2836**	**MBRL 2837**	**MBRL 2839**	**MBRL 2842**	**MBRL 2843**	**MBRL 2847**	**MBRL 2848**	**MBRL 2850**	**MBRL 2851**	**MBRL 2858**
**MBRL 2834**	0 / 0	1103 / 1103	1103 / 1103	1148 / 1148	308 / 308	1590 / 1591	1591 / 1591	1606 / 1606	1606 / 1606	284 / 284	283 / 283	237 / 237
**MBRL 2835**	1103 / 1103	0 / 0	0 / 0	1087 / 1087	1094 / 1095	1571 / 1573	1572 / 1573	1582 / 1583	1582 / 1583	1113 / 1113	1112 / 1112	1090 / 1090
**MBRL 2836**	1103 / 1103	0 / 0	0 / 0	1087 / 1087	1094 / 1095	1572 / 1573	1573 / 1573	1583 / 1583	1583 / 1583	1113 / 1113	1112 / 1112	1090 / 1090
**MBRL 2837**	1148 / 1148	1087 / 1087	1087 / 1087	0 / 0	1148 / 1148	1507 / 1509	1508 / 1509	1503 / 1504	1503 / 1504	1162 / 1162	1162 / 1162	1134 / 1134
**MBRL 2839**	308 / 308	1094 / 1095	1094 / 1095	1148 / 1148	0 / 0	1584 / 1587	1585 / 1587	1599 / 1601	1599 / 1601	339 / 339	338 / 338	306 / 306
**MBRL 2842**	1590 / 1591	1571 / 1573	1572 / 1573	1507 / 1509	1584 / 1587	0 / 0	0 / 0	917 / 918	917 / 918	1590 / 1591	1590 / 1591	1580 / 1581
**MBRL 2843**	1591 / 1591	1572 / 1573	1573 / 1573	1508 / 1509	1585 / 1587	0 / 0	0 / 0	918 / 918	918 / 918	1591 / 1591	1591 / 1591	1581 / 1581
**MBRL 2847**	1606 / 1606	1582 / 1583	1583 / 1583	1503 / 1504	1599 / 1601	917 / 918	918 / 918	0 / 0	0 / 0	1603 / 1603	1603 / 1603	1595 / 1595
**MBRL 2848**	1606 / 1606	1582 / 1583	1583 / 1583	1503 / 1504	1599 / 1601	917 / 918	918 / 918	0 / 0	0 / 0	1603 / 1603	1603 / 1603	1595 / 1595
**MBRL 2850**	284 / 284	1113 / 1113	1113 / 1113	1162 / 1162	339 / 339	1590 / 1591	1591 / 1591	1603 / 1603	1603 / 1603	0 / 0	1 / 1	268 / 268
**MBRL 2851**	283 / 283	1112 / 1112	1112 / 1112	1162 / 1162	338 / 338	1590 / 1591	1591 / 1591	1603 / 1603	1603 / 1603	1 / 1	0 / 0	267 / 267
**MBRL 2858**	237 / 237	1090 / 1090	1090 / 1090	1134 / 1134	306 / 306	1580 / 1581	1581 / 1581	1595 / 1595	1595 / 1595	268 / 268	267 / 267	0 / 0

≤5 related (green), 6-50 possibly related (yellow), ≥51 unrelated (red)
